# A prognostic signature of Glutathione metabolism-associated long non-coding RNAs for lung adenocarcinoma with immune microenvironment insights

**DOI:** 10.3389/fimmu.2025.1477437

**Published:** 2025-02-10

**Authors:** Junxi Hu, Shuyu Tian, Qingwen Liu, Jiaqi Hou, Jun wu, Xiaolin Wang, Yusheng Shu

**Affiliations:** ^1^ Clinical Medical College, Yangzhou University, Yangzhou, China; ^2^ Department of Thoracic Surgery, Northern Jiangsu People’s Hospital, Yangzhou, China; ^3^ Dalian Medical University, Dalian, China

**Keywords:** lung adenocarcinoma, Glutathione metabolism, lncRNA, prognostic prediction, immune microenvironment

## Abstract

**Background:**

Glutathione (GSH) metabolism supports tumor redox balance and drug resistance, while long non-coding RNAs (lncRNAs) influence lung adenocarcinoma (LUAD) progression. This study developed a prognostic model using GSH-related lncRNAs to predict LUAD outcomes and assess tumor immunity.

**Methods:**

This study analyzed survival data from The Cancer Genome Atlas (TCGA) and identified GSH metabolism-related lncRNAs using Pearson correlation. A prognostic model was built with Cox and Least Absolute Shrinkage and Selection Operator (LASSO) methods and validated by Kaplan-Meier analysis, Receiver Operating Characteristic (ROC) curves, and Principal Component Analysis (PCA). Functional analysis revealed immune infiltration and drug sensitivity differences. Quantitative PCR and experimental studies confirmed the role of lnc-AL162632.3 in LUAD.

**Results:**

Our model included a total of nine lncRNAs, namely AL162632.3, AL360270.1, LINC00707, DEPDC1-AS1, GSEC, LINC01711, AL078590.2, AC026355.2, and AL096701.4. The model effectively forecasted patient survival, and the nomogram, incorporating additional clinical risk factors, satisfied clinical needs adequately. Patient stratification based on model scores revealed significant disparities in immune cell composition, functionality, and mutations between groups. Additionally, variations were noted in the IC50 values for key lung cancer medications such as Cisplatin, Docetaxel, and Paclitaxel. *In vitro* cell experiment results showed that AL162632.3 was markedly upregulated, while AC026355.2 tended to be downregulated across these cell lines. Ultimately, suppressing lnc-AL162632.3 markedly reduced the growth, mobility, and invasiveness of lung cancer cells.

**Conclusion:**

This study identified GSH metabolism-related lncRNAs as key prognostic factors in LUAD and developed a model for risk stratification. High-risk patients showed increased tumor mutation burden (TMB) and stemness, emphasizing the potential of personalized immunotherapy to improve survival outcomes.

## 1 Introduction

Lung cancer is among the most prevalent and lethal cancers worldwide, accounting for a significant proportion of all cancer-related deaths (1). Non-small cell lung cancer (NSCLC) comprises the majority of lung cancer cases, with LUAD as the primary subtype, and is more frequently observed in non-smokers and women (2). Despite the numerous existing treatment options, only about 20% of lung adenocarcinoma patients benefit from them, and the majority of patients still experience poor survival outcomes (3). Conventional treatments have limited effects on advanced LUAD, and although targeted therapies and immunotherapy are effective, resistance and tumor heterogeneity still significantly impact treatment outcomes and patient survival rates (4). Recent advances in molecular biology and genomics have revealed complex mechanisms of tumor development, increasingly highlighting the importance of molecular markers in the diagnosis, prognostic assessment, and treatment decision-making (5). The lncRNA, as an emerging molecular marker, exhibits unique functions in regulating gene expression and affecting the tumor microenvironment (6). Exploring new lncRNA molecular markers and related prognostic models can facilitate early cancer screening, precise diagnosis, and the formulation of personalized treatment plans, thereby improving therapeutic effectiveness.

lncRNAs are RNA molecules exceeding 200 nucleotides in length and do not code for proteins (7). Although lncRNAs do not encode proteins, they are critical in gene expression regulation, chromatin modification, and post-transcriptional control. Recently, research on lncRNAs has greatly expanded their applications in the biomedical field, particularly in cancer research (8). For example, MALAT1 (Metastasis Associated Lung Adenocarcinoma Transcript 1) is highly expressed in lung cancer and is closely associated with poor prognosis, promoting tumor metastasis by regulating gene transcription and RNA splicing (9). In breast cancer, high HOTAIR expression promotes chromatin changes, enhancing tumor invasion and metastasis, and correlates with lower survival rates (10). Additionally, H19, a significant lncRNA, regulates multiple signaling pathways in liver and colorectal cancer, affecting tumor cell proliferation and survival (11). Importantly, lncRNAs not only regulate the onset and progression of tumors but also serve as potential therapeutic targets and prognostic markers. For instance, studies have shown that lncRNA-ATB can alter the growth characteristics of liver cancer cells by enhancing the TGF-β signal to promote their metastasis (12). Currently, the specific roles of lncRNAs in the tumor microenvironment and their potential clinical applications still require further exploration.

GSH is a crucial intracellular antioxidant composed of glutamic acid, cysteine, and glycine (13). GSH plays a significant role in maintaining cellular redox balance by reducing hydrogen peroxide and lipid peroxides, thus protecting cells from oxidative stress damage (14). In tumor cells, GSH metabolism pathways are often remodeled to cope with oxidative stress and metabolic demands brought about by rapid proliferation (15). Tumor cells resist the toxicity of chemotherapy drugs by enhancing GSH synthesis or reducing its consumption, leading to chemotherapy resistance (16). Furthermore, it has been found that inhibiting GSH synthesis can enhance the efficacy of immunotherapy, boosting the anti-tumor immune response (17). Investigating the metabolic functions of GSH in tumors aids in the creation of novel therapeutic approaches for tackling tumor resistance and immune escape, ultimately enhancing patient treatment outcomes and survival rates (18).

Through the analysis of TCGA dataset, this research has identified long lncRNAs related to GSH metabolism and has developed a new type of risk assessment model accordingly. We developed and validated a nomogram model that integrates features of GSH metabolism-related lncRNAs with clinical factors. Additionally, based on functional analysis of model differential genes, we also explored whether the model could preliminarily predict drug treatment outcomes for LUAD patients. Finally, we performed preliminary validation of the lncRNAs in the model in both normal and tumor cell lines.

## 2 Materials and methods

### 2.1 Data acquisition

By May 9, 2024, our research had retrieved 600 files from the TCGA official site (https://cancergenome.nih.gov/), encompassing RNA-seq expression matrices and clinical data for 517 LUAD patients. This study utilized Active Perl software for preprocessing the raw files, creating a consolidated table of gene and clinical information. Using the metadata gene annotation file downloaded from the official website, this study converted Ensembl IDs to common official gene names using Active Perl. Before analyzing the expression data, all data were normalized using log2 transformation. Somatic mutation information for all LUAD patients was downloaded from the TCGA official website. After excluding 10 patients with incomplete clinical information, further analysis included 507 patients. This study used the createDataPortion package to randomly split patients into training (355 individuals) and test (152 individuals) groups. Additionally, the grouped data passed clinical statistical difference testing. Based on the annotation files from the Ensembl human genome browser GRCh38, this study performed iterative processing on all genes using Active Perl, extracting the expression matrices for mRNA and lncRNA. Integrating published papers and data from the GSEA official site, this study identified 87 crucial genes. Since all data in this study came from publicly accessible databases and followed TCGA database publication guidelines, ethics committee approval was omitted. This study adhered to the 2013 revised Declaration of Helsinki.

### 2.2 Identification of lncRNAs related to GSH metabolism

Based on the research by Shi et al. (19) and MSigDB, we identified 78 genes associated with GSH metabolism. From the obtained gene list, we extracted expression data for GSH-related genes. The limma software package was utilized for co-expression analysis. Using filters with a Pearson correlation coefficient greater than 0.4 and a *p*-value below 0.001, we identified 1748 lncRNAs associated with GSH metabolism. These thresholds were chosen to balance biological relevance, statistical significance, and clinical translational feasibility. Based on the identified lncRNA gene names, we extracted the corresponding expression matrix for GSH metabolism-related lncRNAs.

### 2.3 Construction and validation of a prognostic model for GSH-related lncRNA characteristics

Employing the Survival software, COX univariate regression analysis was conducted on lncRNA expression and survival data for the training cohort, with a filter threshold of p < 0.001. Subsequently, feature lncRNAs in the prognostic model were selected using a combined LASSO-COX model (20). Additionally, the independent correlation of the key lncRNAs selected in the model must meet the requirements of multiple linear stepwise regression analysis. Concurrently, the minimum Akaike Information Criterion (AIC) is calculated to obtain optimal simulation effects (21). Ultimately, the prognostic model includes 9 lncRNAs, with the risk scoring formula as:


$$\text{Risk}\;\text{score}\;=\;\sum_{\text{i}}^{\text{n}}\text{α}\text{i}\;\times \;\text{β}\text{i}$$

where α i represents the regression coefficient of the ith lncRNA obtained from the multivariate Cox regression analysis, and β i represents the expression value of each GSH metabolism-related lncRNAs.These coefficients are fixed values derived from the training dataset.The expression levels of the selected lncRNAs, which vary with each patient sample, were then multiplied by their respective coefficients to compute the individualized Risk score. Using the “survival” and “survminer” packages, KM curves for OS and PFS were plotted, with statistical differences assessed via the log-rank test. The “timeROC” package was utilized for receiver operating characteristic (ROC) analysis, computing the AUC value. The C-index value was derived through the Hmisc package in conjunction with patient survival information analysis. The “scatterplot3d” package was used for PCA principal component analysis and visualization of model-related lncRNAs, all genes, and GSH-related genes and lncRNAs.

### 2.4 Construction and calibration of predictive nomograms

In this study, we used the median risk score as the cutoff value for grouping, evenly distributing patients into high-risk and low-risk groups. By integrating routine clinical data with model risk assessments, this study utilizes the rms, regplot, and Survival packages to construct clinically relevant nomograms. These nomograms aim to accurately predict patients’ overall survival (OS) rates. Furthermore, we meticulously generated the corresponding calibration curves to validate the predictive accuracy.

### 2.5 Functional enrichment analysis

Using the limma software package, we identified differentially expressed genes between high and low risk groups of LUAD patients (log2FC>1,FDR<0.05). According to the descending order of p-values, we selected the top ten significant and meaningful results. These results were visualized using different packages like ggplot2, circlize, and ComplexHeatmap (21, 22). The GSEA software package was used for gene set enrichment analysis, comparing pathway gene sets in the KEGG database to evaluate the association of lncRNAs involved in model construction with these gene sets. Finally, we visualized the top five results using the clusterProfiler (23) and enrichplot packages.

### 2.6 Analysis of tumor mutational burden and tumor immune microenvironment

Through the CIBERSORT package, the immune infiltration results in tumor samples of LUAD patients were obtained. The study comprehensively assessed how model risk scores correlate with various immune cell types using multiple algorithms (24–30). The GSVA package evaluated the correlation between 13 immune function genes and LUAD transcriptome expression (31). The Maftools software package (32) is used to process and analyze the proportions of missense mutations, nonsense mutations, frameshift deletions, frameshift insertions, inframe deletions, and multiple mutations in high-frequency mutated genes and tumor-related genes.

### 2.7 Prediction of immunotherapy efficacy and potential chemotherapeutic drug screening

The limma package integrated the immunotherapy data with patient risk group information, and the ggpubr package generated violin plots for cohorts. Screening of potential chemotherapeutic drugs is conducted using the oncoPredict package (33). Obtain the GDSC2 training dataset (https://www.cancerrxgene.org/) containing 198 chemotherapy drugs from the oncoRespond section on the OSF site. Conduct drug sensitivity analysis using the calcPhenotype function to calculate IC50. Set the significance filtering threshold to a *p*-value less than 0.001.

### 2.8 cell line culture

HBE cells and NSCLC cell lines were acquired from the Chinese Cell Resource Center. Cells were cultured in complete medium with 10% fetal bovine serum (FBS, Gibco brand), under incubator conditions of 5% CO2, 37°C, and 95% humidity.

### 2.9 Cell transfection

RNAi reagents and transfection aids were supplied by genepharma, with detailed interference sequences available in **Supplementary Table 1**. Cells were plated a day before transfection, ensuring that cell density reached approximately 50%-60% confluency on the day of transfection. RNAi reagents were preincubated with transfection enhancers and subsequently allowed to equilibrate at room temperature for 20 minutes to optimize complex formation. During the resting period, medium exchange was conducted, and the settled transfection complex was introduced to the culture plates, with timely replenishment or replacement of the medium depending on cell growth conditions. Transfection efficiency was assessed 48 hours after the procedure.

### 2.10 Real-time quantitative PCR

Total RNA was extracted from cells using TRIzol reagent as per the protocol outlined in the manual (Vazyme, Cat No. R701-01). The RNA concentration and purity were then assessed using the Nanodrop 2000 spectrophotometer. By adhering to the cDNA synthesis kit’s instructions (Vazyme, Cat No. Q141-02/03), reverse-transcribed cDNA was generated through RT-PCR amplification. The quantitative results of gene expression were collected and analyzed on the StepOne Plus system. The data were analyzed using the 2^(-ΔΔCt) method. GAPDH was used as an internal reference gene to normalize the target genes.

### 2.11 colony formation assays

Cells in good condition 48 hours post-transfection were selected for counting. Both experimental and control plates were prefilled with serum-free medium. Based on the calculations, 500 cells were evenly seeded per well and incubated for two weeks. After culturing, cells were washed twice with PBS and fixed in 4% paraformaldehyde for 10 minutes. After a PBS wash, cells were stained with crystal violet for 10 minutes, rinsed under running water for 5 minutes, and the plates were dried and photographed.

### 2.12 CCK8 proliferation assays

Digest and count cells in good condition and in the logarithmic growth phase after transfection, adjusting the cell density to 5×10³ cells/ml with blank medium. Add 200 µl of the adjusted cell suspension to each well of a 96-well plate, and measure the optical density (OD) at 24, 48, 72, and 96 hours post cell adherence. Subsequently, 10 µl of CCK8 reagent was meticulously added to each well, followed by an incubation period of 2 hours. Absorbance was then precisely measured at 450 nm using a spectrophotometer.

### 2.13 Cell invasion assay

Prior to the experiment, evenly distribute the matrix gel at the bottom of the Transwell chambers and incubate until the gel completely solidifies. On the experiment day, select well-conditioned and logarithmically growing post-transfection cells for trypsin digestion and counting. Resuspend the cells in serum-free medium, adjusting the density to 2.5×10^5 cells/ml. Extract the Transwell chambers, aspirate excess medium or liquid from the upper compartment, and add 200 µl of adjusted cell suspension to it, along with 500 µl of complete medium to the lower compartment. Continue culturing the Transwell chambers in the incubator for 48 hours, then proceed with further processing. Remove the old medium, wash the chambers twice with PBS, and fix the cells in 4% paraformaldehyde for 10 minutes. After removing the fixative, apply 0.1% crystal violet stain for 10 minutes. Next, remove the staining solution and wash the Transwell chambers three times with deionized water to eliminate excess dye. Lastly, use a damp cotton ball to gently wipe the upper surface of the chamber, removing cells that have not penetrated the matrix gel. Allow the chambers to air dry at room temperature, then examine and photograph the results under a microscope.

### 2.14 Wound healing test

Prior to the experiment, each well of the culture plate is prefilled with 2 ml of blank medium. Digest and count cells in good condition and in the logarithmic phase after transfection, resuspending them in blank medium. Add 400,000 cells evenly to each well. Once cells adhere, remove them using a 200 µl pipette tip and wash once with PBS. Add blank medium and incubate for 48 hours. Remove the plate, wash with PBS, and use a microscope to photograph and document the scratch healing process.

### 2.15 Xenograft model

Ten genetically defined, 5-week-old female nude mice were procured from the Jiangsu Jicui official website and randomly assigned into two groups. Prior to the experiment, target cells and empty vector control cells, both in optimal condition and the logarithmic growth phase, were precultured. Cells were trypsinized, resuspended in sterile Phosphate-Buffered Saline (PBS), and the concentration was adjusted to 4×10^3 cells/µl. Each mouse received a subcutaneous injection of 200 µl of this suspension into the right axillary region. Tumor dimensions were measured every three days with calipers. After one month, the mice were euthanized, and the tumors were excised, weighed, and measured for volume. The study was approved by the Yangzhou University Animal Experiment Ethics Review Committee, adhering strictly to ethical guidelines.

### 2.16 Statistical analysis

Statistical analysis was conducted in R (version 4.3.0). For evaluating the differences between pairs of groups, the Wilcoxon rank-sum test was employed. In contrast, differences across multiple groups were determined using the Kruskal-Wallis test. Results were deemed statistically significant at a *p*-value below 0.05.

## 3 Results

### 3.1 Identification of GSH metabolism-related lncRNAs

**Figure 1:**
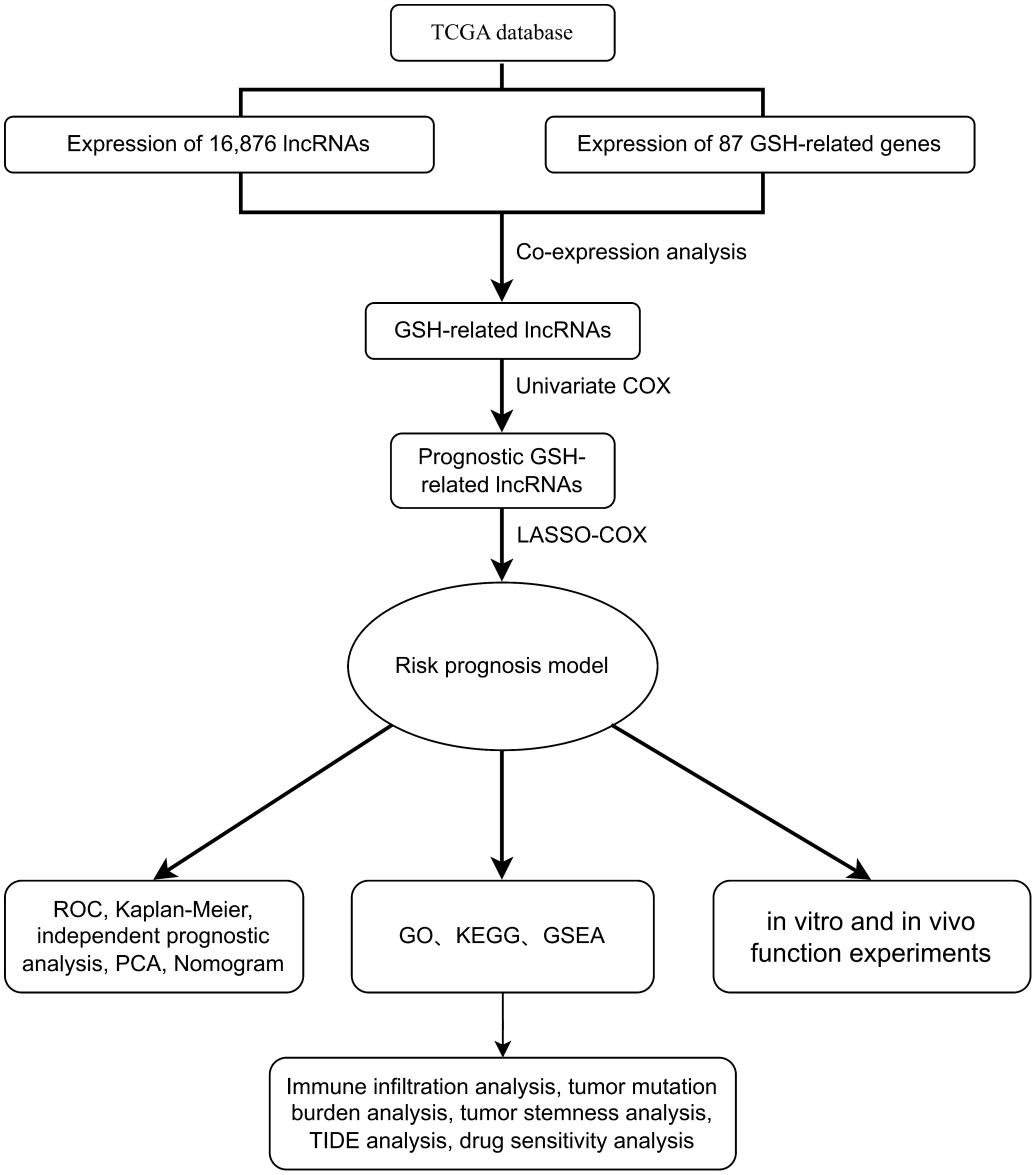
Flow diagram.

**Figure 2:**
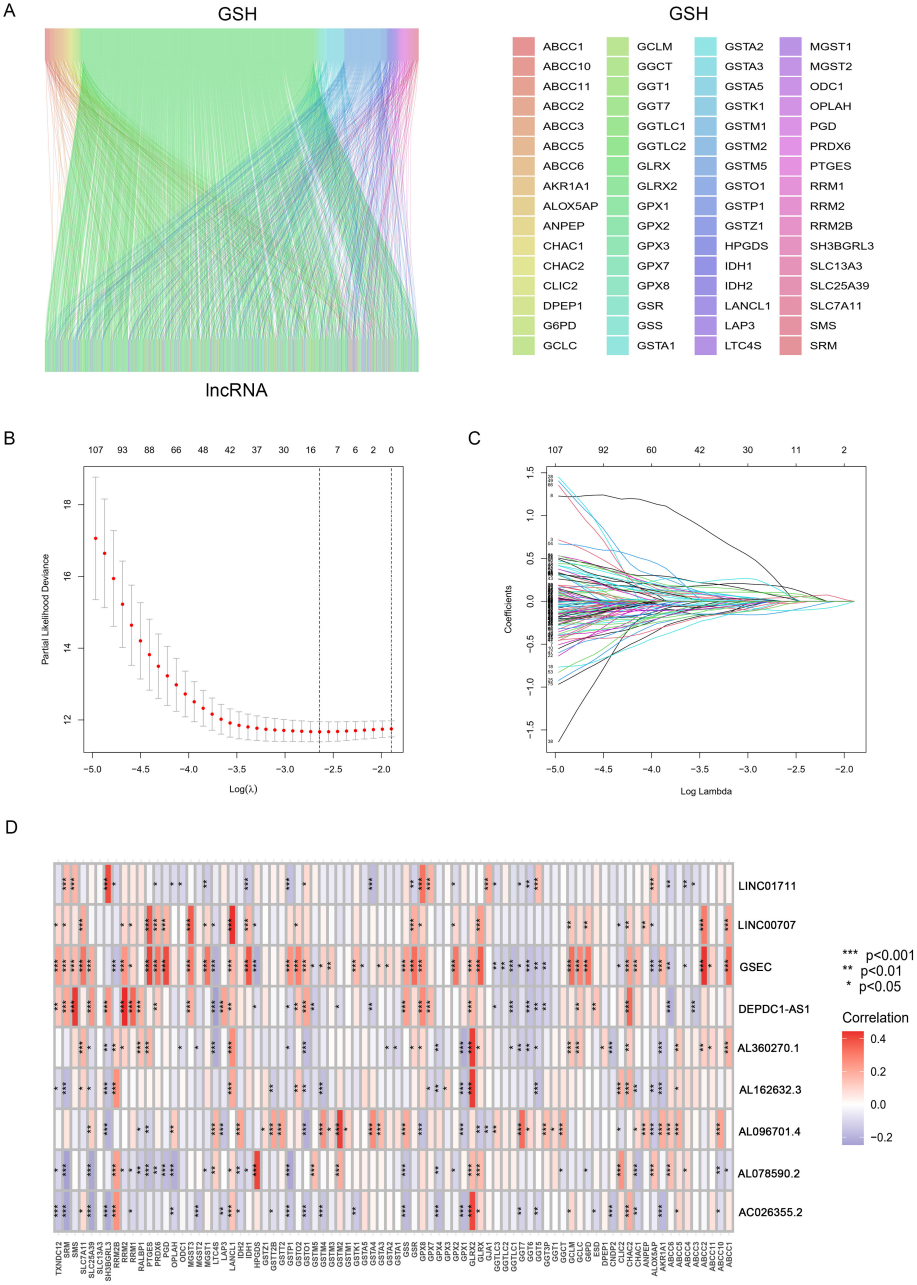
Identification and Prognostic Model Construction of GSH Metabolism-related lncRNAs. **(A)** The Sankey diagram shows the co-expressed GSH metabolism-related genes and lncRNAs. **(B)** ten-fold cross-validation. **(C)** LASSO coefficient curves. **(D)** Correlation heatmap between lncRNAs and gene sets.

**Figure 1** showed the flowchart of this study. This study included samples from 541 cancer patients, identifying 16,876 lncRNAs from 59,427 genes in the transcripts. The GSH-related gene set used in this study included 87 genes such as ABCC1, ABCC4, ABCC5. A comprehensive analysis revealed 1,748 lncRNAs associated with GSH metabolism, characterized by a correlation coefficient exceeding 0.4 and a *p*-value below 0.001 (**Figure 2A**).

### 3.2 Development and assessment of the prognostic GSH-based risk model

**Figure 3:**
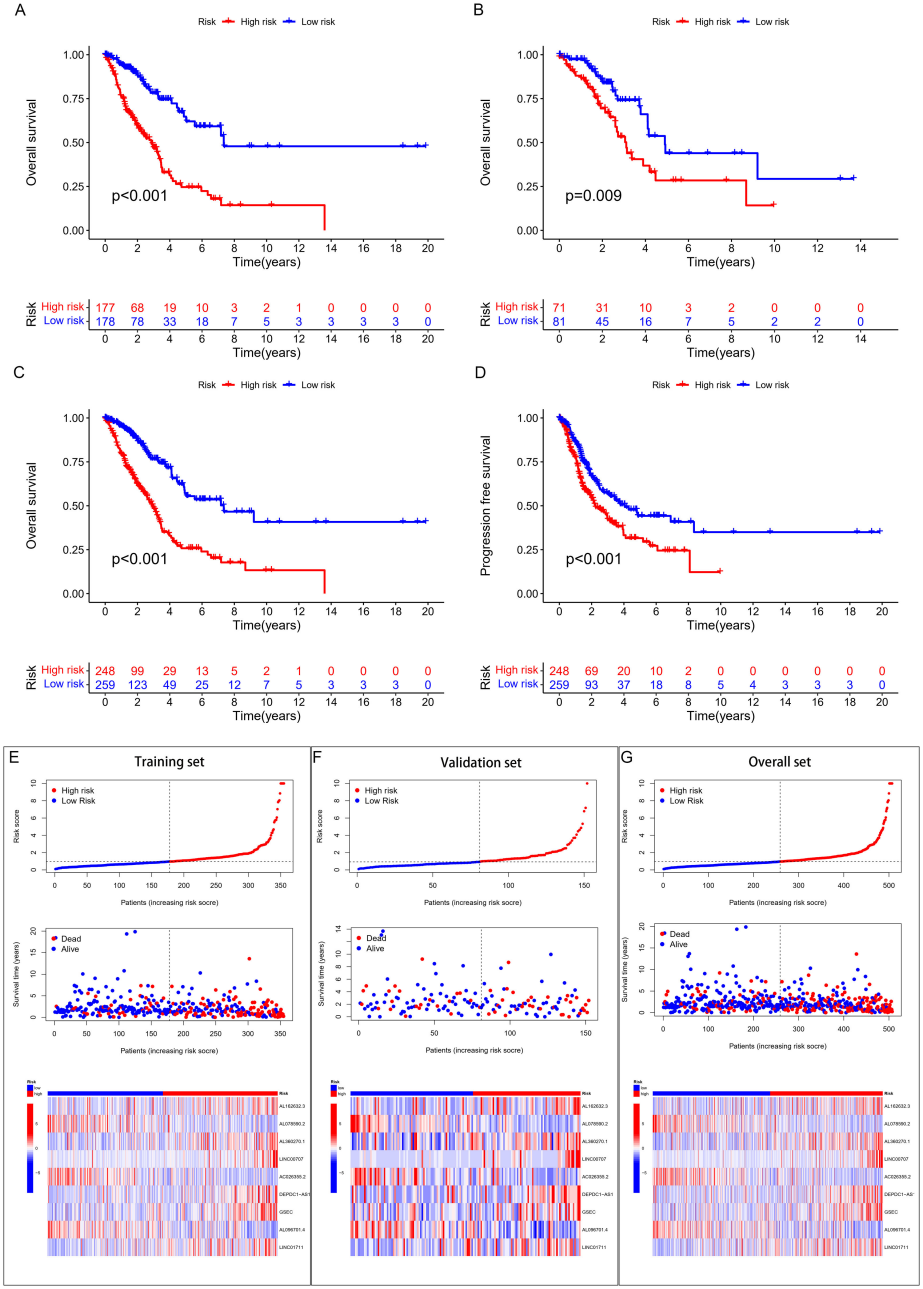
Survival analysis and validation. **(A)** K-M curves for OS in the training set, **(B)** validation set, **(C)** overall cohort. **(D)** K-M curves for PFS in the overall cohort. **(E)** (training set), **(F)** (validation set), **(G)** (overall set) risk score plots, survival status graphs, and heatmaps.

This study included 507 samples for the construction of a GSH-related lncRNA risk model. Using the createDataPartition package, we randomly assigned all samples into a training set (355 persons) and a testing set (152 persons), without statistical differences in clinical data between the two groups (**Supplementary Table 2**). Through LASSO-COX analysis (P<0.05), we identified 9 lncRNAs significantly associated with prognosis (**Figures 2B, C**; **Supplementary Table 3**). Risk scores for patients in both the training and testing groups were calculated using the formula, and patients were divided into high and low risk groups based on the median value (**Supplementary Table 4**). The correlation heatmap in **Figure 2D** showed the relationship between the GSH metabolism gene set and the model-building lncRNAs. The K-M analysis demonstrated that high-risk patients have significantly lower overall survival (OS) than low-risk patients in both the training and testing groups (**Figures 3A–C**). The PFS analysis results were consistent with the aforementioned OS results, P<0.05 (**Figure 3D**). The risk curves indicated a positive correlation between rising risk scores and increasing patient mortality. The correlation heatmap showed that the expression of lncRNAs such as AL162632.3, AL360270.1, LINC00707, DEPDC1-AS1, GSEC, and LINC01711 increases with rising risk scores, while the expression of AL078590.2, AC026355.2, and AL096701.4 decreases (**Figures 3E–G**).

### 3.3 Validate the independence of the constructed model in LUAD

**Figure 4:**
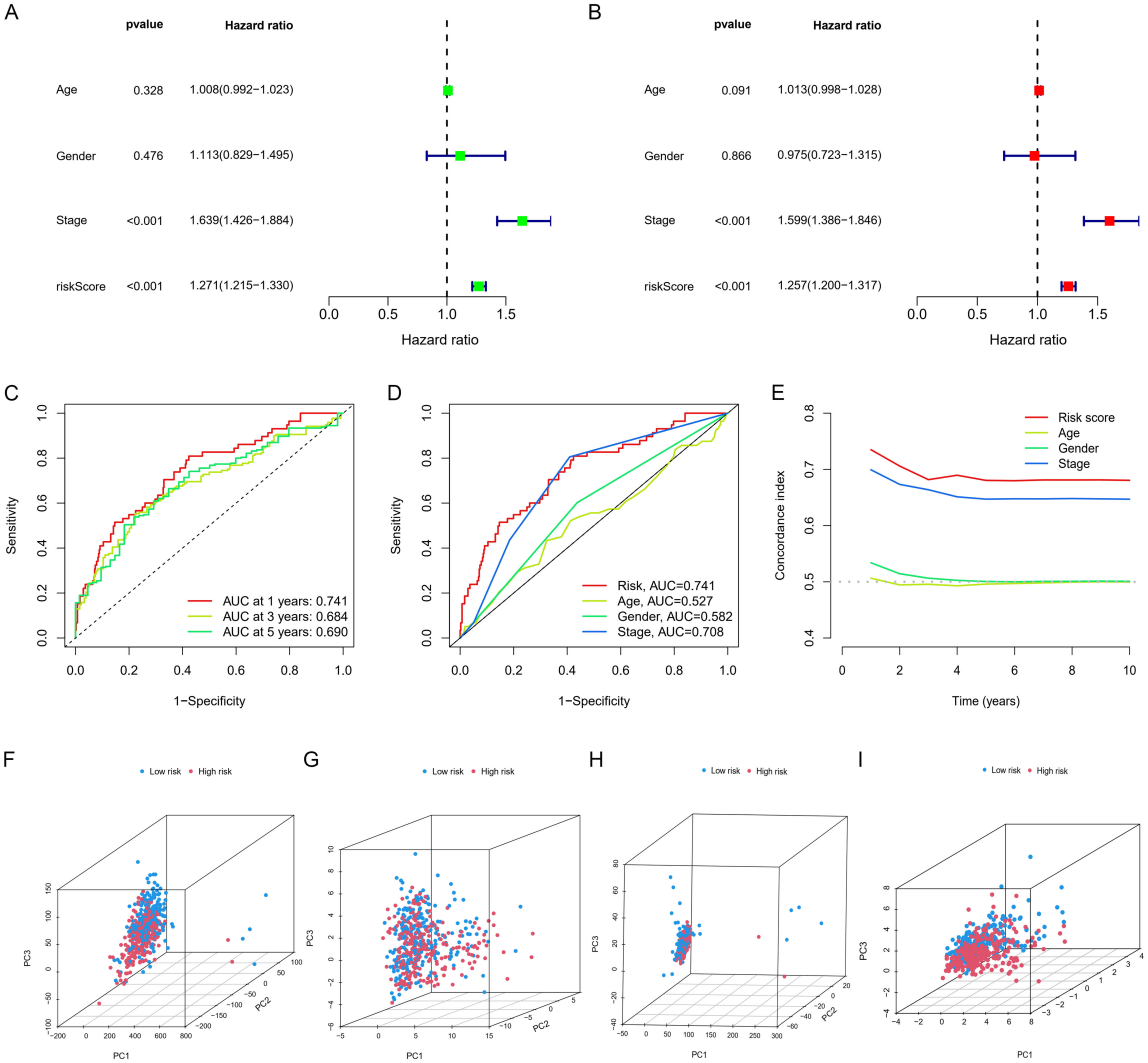
Independent prognostic analysis and PCA analysis. **(A, B)** Cox regression analysis. **(C)** Time-dependent ROC curves for overall survival (OS). **(D)** ROC curves and **(E)** C-index for risk scores and other clinical risk factors. **(F)** PCA for the whole genome, **(I)** GSH metabolism genes, **(G)** all GSH metabolism-related lncRNAs, and **(H)** model-related lncRNAs.

**Figure 5:**
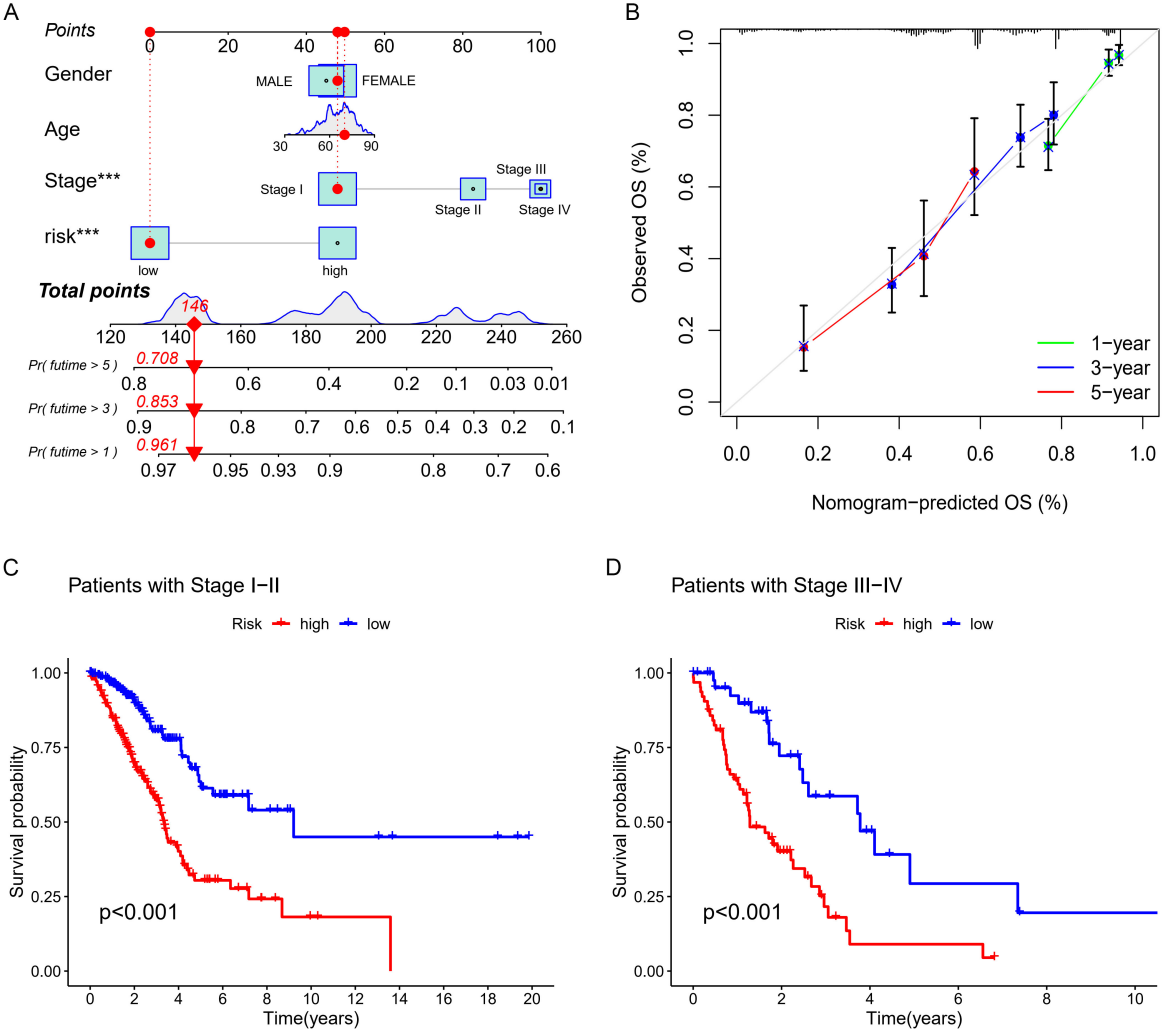
Nomogram Development and Validation **(A)** Nomogram. **(B)** Corresponding calibration curve. **(C, D)** K-M analysis of OS.

Independent prognostic evaluation consistently showed that the model yielded hazard ratios (HRs) exceeding 1, with highly significant *p*-values observed across univariate and multivariate Cox regression analyses (**Figures 4A, B**). We conducted ROC curve analyses to explore the model’s predictive capability on patient prognosis (**Figure 4C**). The prognostic model demonstrated the highest AUC and C-index values compared to clinical factors (e.g., age and stage) (**Figures 4D, E**). To investigate the model’s applicability across different cancer stages, we divided patients into early and late stages and conducted OS analysis; the results demonstrated that, in both early and late stages, the high and low-risk scores clearly distinguished between patients’ survival durations (**Figures 5C, D**).

### 3.4 PCA and nomogram

PCA presented in **Figures 4F–I** showed that the lncRNA model we developed could best distinguish patients. **Figures 5A, B** showed the clinical nomogram, where each clinical factor is scored, and the sum of all factors’ scores was the total composite score. According to the scale for the composite score, one can estimate the survival probabilities for 1 year, 3 years, and 5 years for patients. For instance, a patient with a total score of 146 exhibited survival probabilities of 0.961 at 1 year, 0.853 at 3 years, and 0.708 at 5 years, respectively.

### 3.5 Functional enrichment analysis

**Figure 6:**
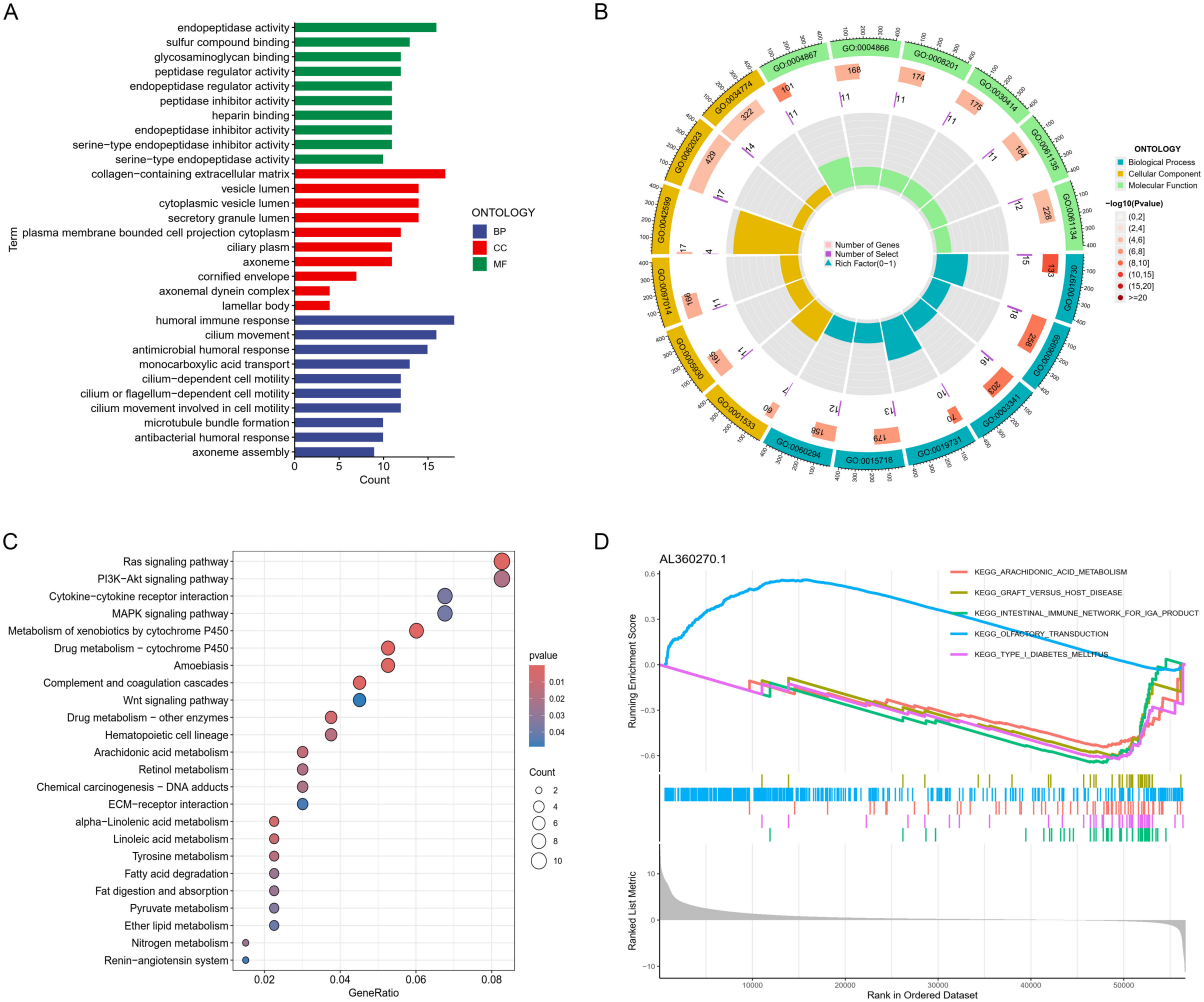
Gene function enrichment analysis. **(A)** Bar graphs and **(B)** chord diagrams displaying significant GO enrichment outcomes. **(C)** Bubble charts illustrating significant KEGG enrichment findings. **(D)** GSVA analysis for lncRNA (AL360270.1).

We conducted a detailed investigation into the prognostic influence of critical lncRNAs on LUAD survival by differentially analyzing high and low risk groups (logFC > 1, FDR < 0.05), identifying 447 significant genes (**Supplementary Table 5**). GO functional analysis indicated that the differentially expressed genes primarily cluster in immune cell infiltration (like chemotaxis induction, migration of myeloid cells), immune activation (such as antimicrobial humoral response (GO:0019730), humoral immune response (GO:0006959), and antimicrobial peptide-driven humoral immunity (GO:0061844)), extracellular matrix (ECM) remodeling (including collagen-rich ECM, cytoplasmic vesicles, lamellar bodies), and cytokine-cytokine receptor interactions (such as regulation of endopeptidase activity) (**Figures 6A, B**).The results of the KEGG pathway analysis, represented through bubble charts and histograms, demonstrated an enrichment of these different genes in pathways that regulate immune cells, including the ras signaling pathway, the pi3k-akt signaling pathway, and cytokine-cytokine receptor interactions (**Figure 6C**). GSEA analyses were conducted for each gene within the model, such as AL360270.1, which showed that most lncRNAs are linked to pathways involved in cancer, immunity, and metabolism (**Figure 6D**).

### 3.6 Evaluation of tumor-immune landscape and analysis of immune related function

**Figure 7:**
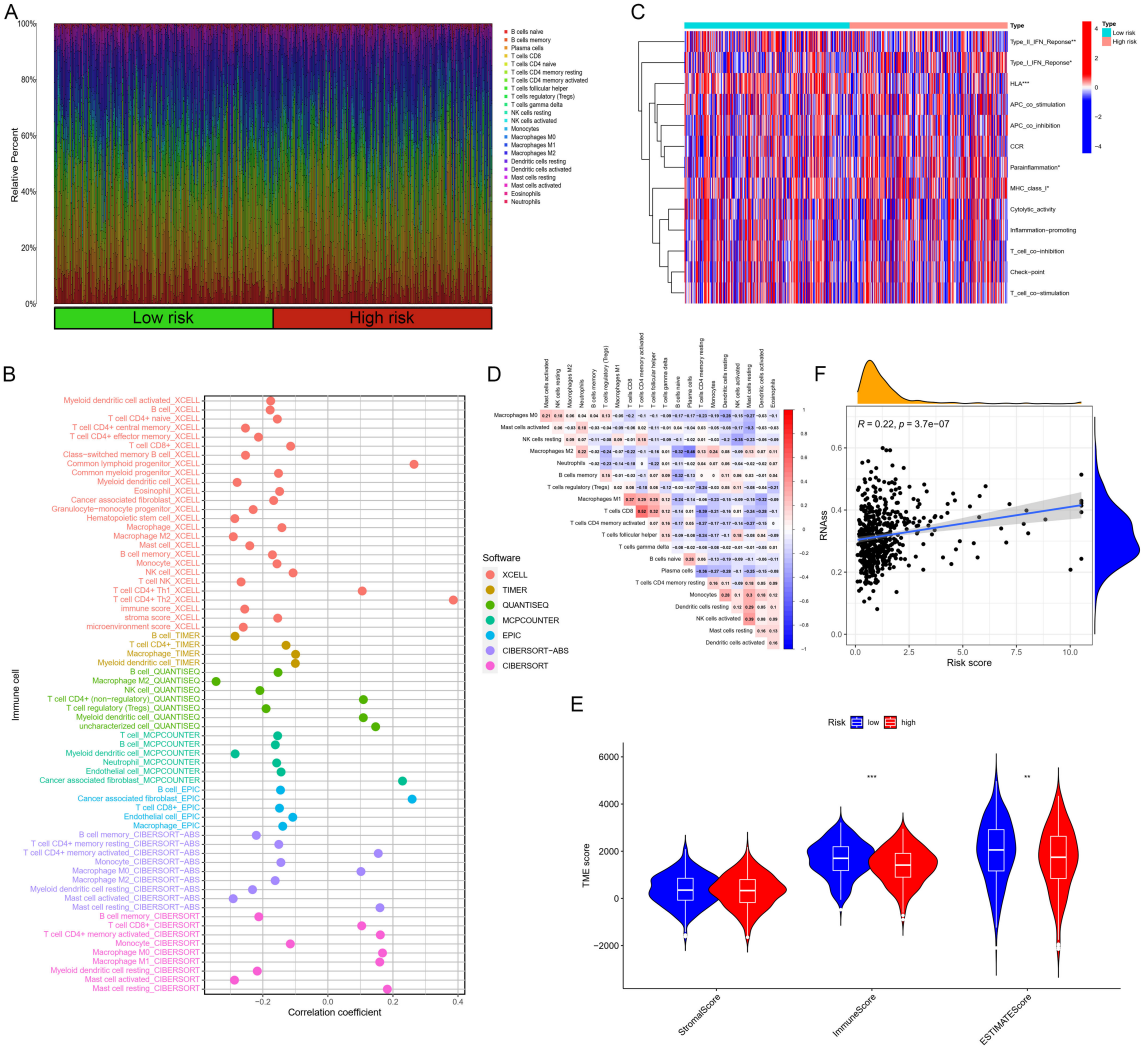
Immunological relevance analysis. **(A)** Stacked bar charts showing the composition ratios of classic immune cells in LUAD patient samples. **(B)** Bubble charts showing immune cell infiltration. **(C)** Analysis of the correlation between risk scores and immune pathways. **(D)** Correlation heatmap. **(E)** Violin plots showing the stroma, immune, and estimated scores. **(F)** Correlation analysis between risk scores and tumor stemness.

**Figure 7A** showed the proportions of typical immune cells in LUAD patient samples from the TCGA database. **Figure 7E** demonstrated that while there were no significant differences in stromal cell scores, patients classified as high-risk exhibited notably lower immune cell scores within TME and overall estimation scores compared to those at low risk. We employed tools like XCELL, TIMER, QUANTISEQ, and MCPCOUNTER for correlation analysis, as depicted in the bubble chart of **Figure 7B**. We further explored the interactions between these immune cells. **Figure 7D** depicted the correlations between immune cells in the tumors of these patients. **Figure 7C** showed the heatmap of correlations between LUAD samples and immune functions, indicating significant differences between the high and low risk groups in classical immune functions such as Type II IFN Response, Type I IFN Response, HLA, etc.

### 3.7 TMB

**Figure 8:**
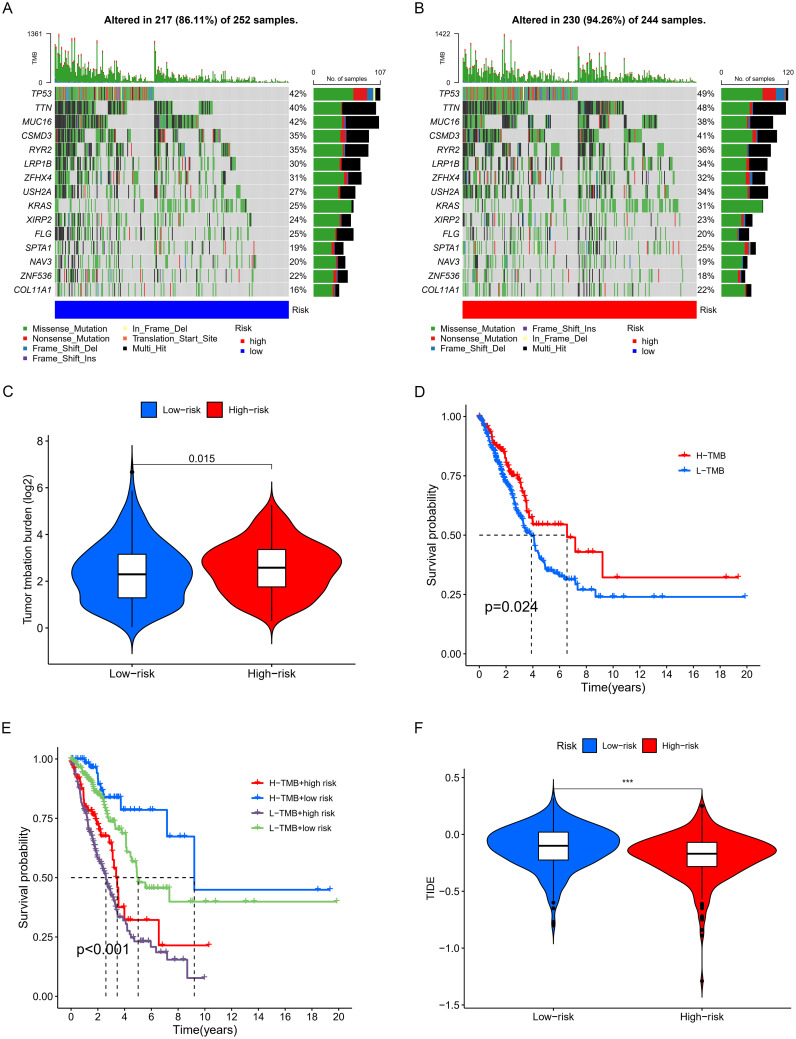
Examination of TMB. **(A, B)** Waterfall plots displaying notable gene mutations. **(C)** Comparative analysis of TMB. **(D)** K-M curve analysis evaluating the effect of high and low TMB on overall survival (OS). **(E)** K-M curve analysis of patient overall survival (OS) according to TMB and risk scores. **(F)** Analysis of the relationship between risk scores and immunotherapy responses. *** p < 0.001.

Tumor mutational burden analysis revealed higher mutation rates in key genes including TP53, KRAS, and COL11A1 in the high-risk group. Waterfall and violin plots demonstrated that the tumor mutational burden in the high-risk group is significantly higher than that in the low-risk group (**Figures 8A–C**). Kaplan-Meier analysis showed significantly longer survival in patients with high TMB compared to those with low burden (**Figure 8D**). Finally, we divided all patients into four groups based on risk scores and levels of tumor mutational burden to analyze differences in survival. The results showed that high TMB and low-risk groups had the longest survival time, while those in the low TMB and high-risk groups had the shortest survival time, with statistically significant differences in survival among the four groups (**Figure 8E**).

### 3.8 Immunotherapy for risk signature and prediction of potential drugs.

**Figure 9:**
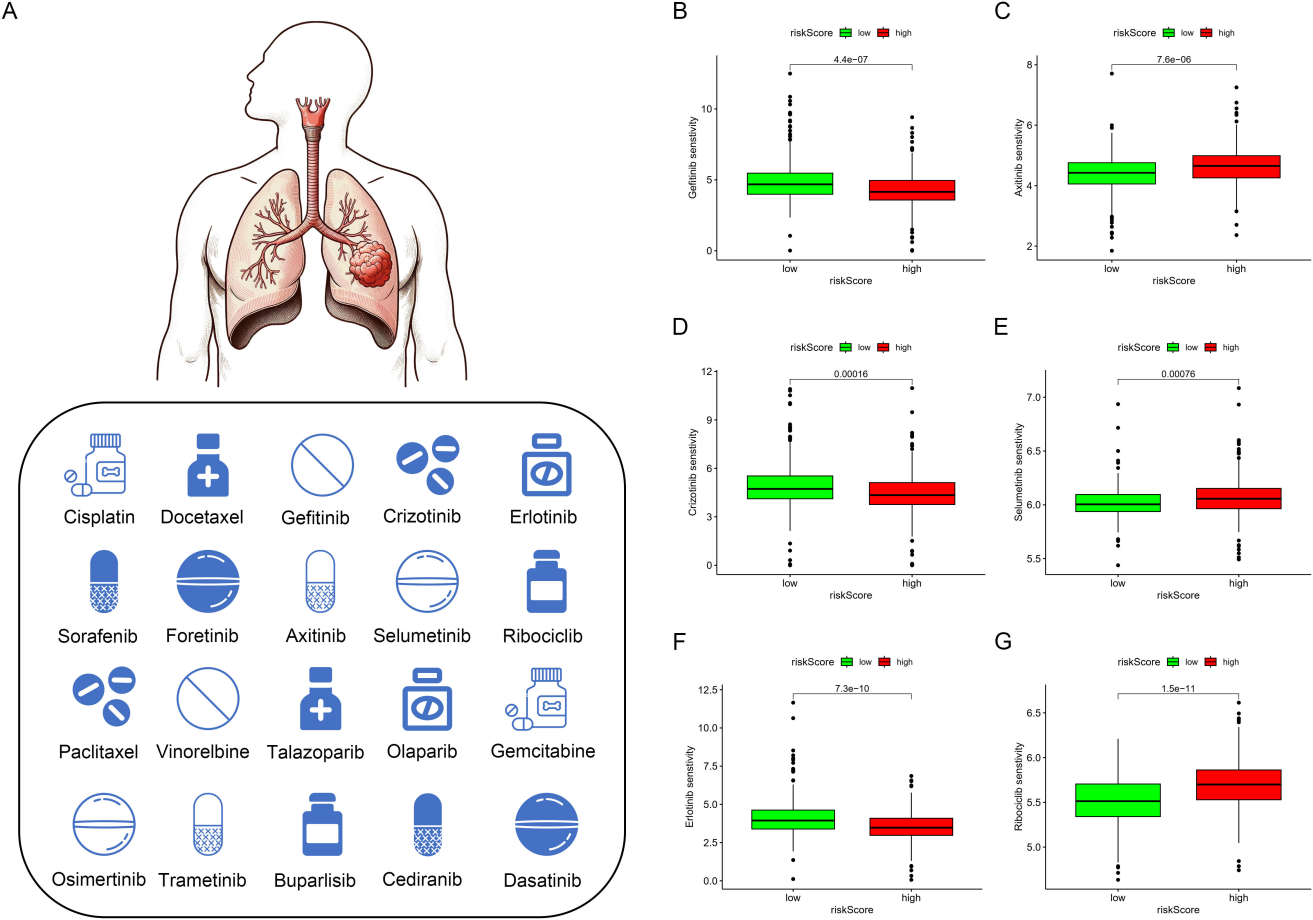
Drug sensitivity analysis. **(A–G)** Currently Approved and Investigational Sensitive Drugs for Lung Cancer Based on Model Predictions.

Based on the discovery that risk scores were closely related to immunity, we further used existing TIDE data to predict patient drug response. The findings reveal markedly reduced TIDE scores in the high-risk cohort relative to the comparison group (**Figure 8F**). Furthermore, analysis of tumor stem cell indices demonstrated a positive correlation with risk scores, indicating increased tumor stemness (**Figure 7F**). Subsequently, we used the oncoPredict package to screen for potential sensitivity drugs. The results indicated that drugs like Cisplatin, Docetaxel, Gemcitabine, Vinorelbine, and Paclitaxel significantly differed in IC50 values (**Figure 9**). Detailed results were presented in the **Supplementary Files**.

### 3.9 Expression confirmed through *in vitro* experiments

**Figure 10:**
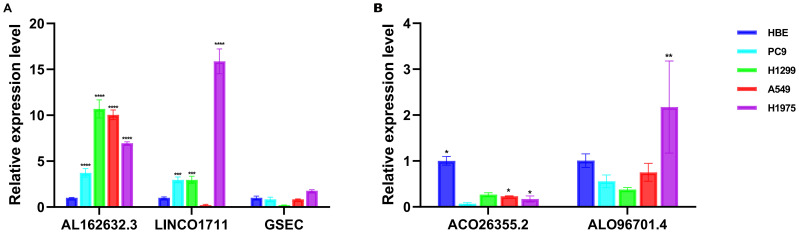
Validation of the expression of lncRNAs *in vitro* cell experiments. Analysis of expression levels of lncRNAs associated with **(A)** risk factors and **(B)** protective factors in HBE, PC9, H1299, A549, and H1975. * p < 0.05, ** p < 0.01, *** p < 0.001, **** p < 0.0001.

We used qPCR to verify the expression levels of lncRNAs with independent prognostic significance to validate the importance of lncRNAs in the model. Compared to the normal cell line HBE, the expression of AL162632.3 significantly increased in the selected cancer cell lines PC9, H1299, A549, and H1975, particularly in H1299 cells. Expression of LINC01711 rose in lung cancer cell lines including PC9, H1299, and H1975 but fell in A549.GSEC showed no significant changes in expression in these cancer cell lines (**Figure 10A**). AC026355.2 was significantly downregulated in all four cancer cell lines. Conversely, expression of AL096701.4 declined in the PC9, H1299, and A549 cell lines, while it increased in H1975 (**Figure 10B**).

### 3.10 Decreased lnc-AL162632.3 expression inhibited LUAD proliferation, migration and invasion

**Figure 11:**
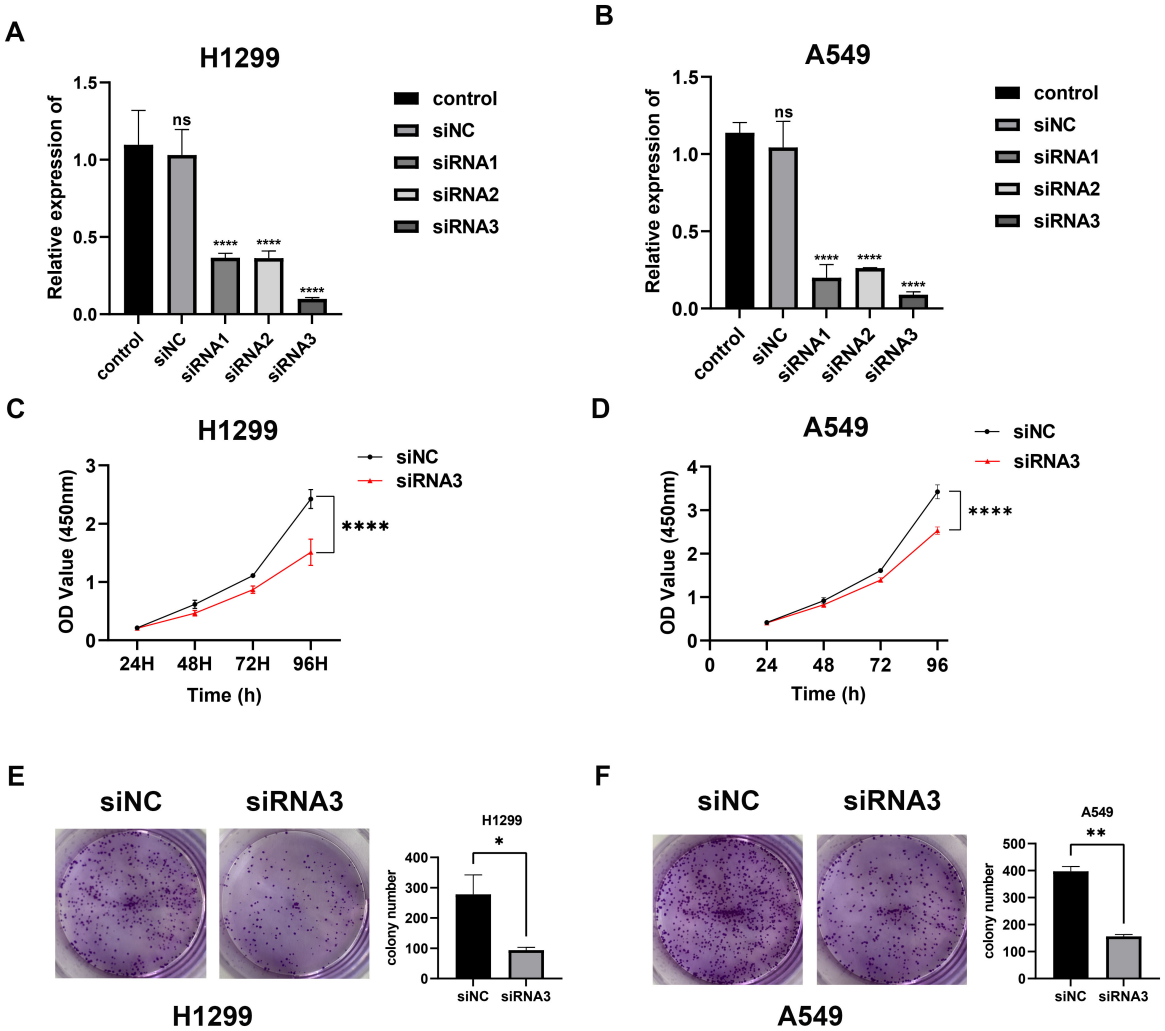
Downregulation of lnc-AL162632.3 inhibits LUAD growth. **(A, B)** Verification of interference efficiency. **(C, D)** CCK8 proliferation curves. **(E, F)** Colony formation assay. * p < 0.05, ** p < 0.01, *** p < 0.001, **** p < 0.0001.

**Figure 12:**
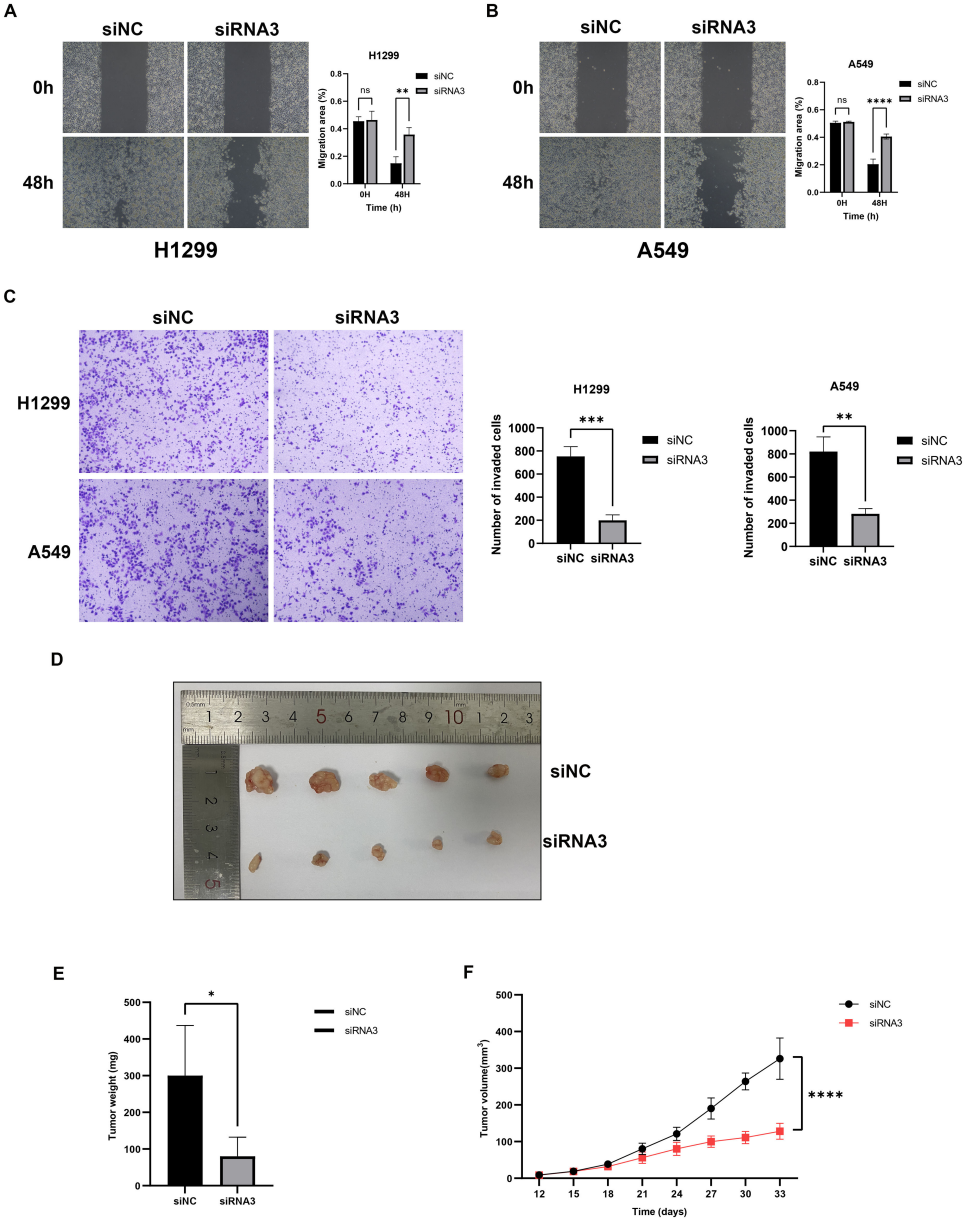
Downregulation of lnc-AL162632.3 inhibits migration and invasion in LUAD. **(A, B)** Wound healing assay. **(C)** Invasion assay. **(D-F)** Xenograft tumor model in nude mice. * p < 0.05, ** p < 0.01, *** p < 0.001, **** p < 0.0001.

Using RNAi technology, we effectively knocked down the highly expressed lnc-AL162632.3 in H1299 and A549 lung cancer cell lines. The RNAi efficiency was validated through qPCR, identifying RNA3 as the most effective. Consequently, we selected siRNA3 for all subsequent experiments. CCK8 proliferation and colony formation assays showed that knocking down lnc-AL162632.3 significantly reduced cell growth compared to the control group. Wound healing and invasion assays further demonstrated that suppression of lnc-AL162632.3 markedly weakened the motility of the cancer cells (**Figure 11**). *In vivo*, tumors in the lnc-AL162632.3 knockdown mice were noticeably smaller and lighter than the controls (**Figure 12**).

## 4 Discussion

Lung cancer is one of the most common malignancies worldwide and is a leading cause of cancer-related deaths (1). In China, LUAD represents the predominant subtype of non-small cell lung cancer (34). The majority of patients are diagnosed at advanced stages, at which point surgery is often no longer viable. Despite some effectiveness of targeted and immunotherapies, the issue of resistance persists, resulting in poor long-term survival rates. Hence, the investigation and detection of molecular markers are crucial for identifying patients suitable for specific therapies and monitoring resistance, thereby enhancing treatment strategies and prognoses. GSH metabolism is pivotal in the onset, progression, and immune response of tumors (35, 36). GSH regulates cell proliferation, apoptosis, and immune function (37). Novel therapeutic approaches targeting GSH metabolism could enhance treatment efficacy and address drug resistance. LncRNAs have been reported to play an irreplaceable role in many cancers, particularly in lung cancer research, with examples including MALAT1 and LINC00707 (9, 36). This study aims to investigate and validate GSH metabolism-related lncRNAs in LUAD, to assess their role in prognosis and immune response, and to enhance personalized treatment outcomes.

Initially, lncRNAs related to GSH metabolism were identified through co-expression analysis, and prognostically significant lncRNAs were selected using LASSO-Cox analysis. The criteria for lncRNA selection (Pearson correlation > 0.4, p < 0.001) were established to balance biological relevance, statistical significance, and clinical feasibility. This ensured the identification of highly relevant lncRNA features, enhancing model interpretability and predictive accuracy and avoiding unnecessary complexity. Six risk factors (AL162632.3, AL360270.1, LINC00707, DEPDC1-AS1, GSEC, and LINC01711) and three protective factors (AL078590.2, AC026355.2, and AL096701.4) were included. LINC00707 has been shown to play significant biological roles in various cancers by interacting with Smad proteins to regulate TGFβ signaling and promote cancer cell invasion (38). Its oncogenic function is further supported by studies in breast and gastric cancers, where elevated LINC00707 expression is associated with reduced patient survival (39). DEPDC1-AS1, an antisense RNA of DEPDC1, promotes proliferation and migration of human gastric cancer cells HGC-27 via the R-F11R pathway (40). LINC01711 can also promote hepatic fibrosis cell proliferation and migration by regulating XYLT1 (41). Moreover, this study is the first to report that AL162632.3, AL360270.1, GSEC, AL078590.2, AC026355.2, and AL096701.4 may be related to the prognosis of LUAD. Although not previously associated with tumor prognosis, these lncRNAs provide new insights into LUAD pathogenesis and warrant further investigation.

Samples were randomly divided into training and validation sets at a 7:3 ratio, and patients were stratified into high- and low-risk groups using the median risk score. Kaplan-Meier analysis showed significantly worse prognosis for the high-risk group, which was consistent across different cohorts, with DFS and risk curves aligning with OS results. The model outperformed clinical factors in predicting LUAD prognosis, as confirmed by ROC and C-index analyses. PCA demonstrated that the lncRNAs in the model effectively differentiated risk groups. The nomogram integrating clinical features enabled personalized prognostic stratification for post-surgical LUAD patients. High-risk individuals may benefit from additional treatments, such as immunotherapy or adjuvant chemotherapy. Enhanced follow-up strategies could further aid in early recurrence detection.

Functional analysis of differentially expressed genes revealed enrichment in immune-related pathways, including cytokine-cytokine receptor interactions and hematopoietic lineage. These findings highlight their critical role in immune regulation. CIBERSORT analysis showed increased immunosuppressive Tregs and M2 macrophages in the high-risk group, alongside reduced CD8+ T cells and M1 macrophages, indicating an immunosuppressive microenvironment conducive to tumor progression. Furthermore, the high-risk group exhibited impaired immune functions, including reduced Type II interferon responses. In contrast, the low-risk group demonstrated enhanced MHC class I expression and cytotoxic activity, underscoring the importance of immune modulation in prognosis and therapy.

Mutation analysis revealed significantly higher TMB in the high-risk group, primarily driven by mutations in key genes such as TP53, KRAS, and MUC16 (42–44). High TMB correlated with better survival rates (p=0.024), but patients in the high-risk group with low TMB had the worst outcomes, which is likely attributable to reduced immunogenicity and increased tumor aggressiveness. Conversely, patients with high TMB and low-risk scores had the best survival, suggesting a favorable response to immunotherapy. Tumor stemness analysis showed that higher RNAss correlated with increased risk scores (R = 0.22, p = 3.7e−07). This finding indicates that high-risk tumors possess stronger stemness traits linked to aggressiveness and treatment resistance. Clinically, targeting tumor stemness could improve patient outcomes. TIDE analysis revealed that low-risk tumors, despite their better prognosis, exhibited strong immune evasion features, highlighting the importance of developing personalized immunotherapy strategies. Chemotherapy sensitivity analysis showed reduced efficacy of standard agents in the high-risk group but increased sensitivity to targeted therapies like Selumetinib, Ribociclib, and Axitinib. These findings highlight the reliance of high-risk tumors on specific pathways, providing guidance for future therapeutic strategies.

Additionally, we conducted independent prognostic analyses on 9 lncRNAs involved in the model construction and identified 5 statistically significant lncRNAs: 3 risk factors (AL162632.3, LINC01711, and GSEC) and 2 protective factors (AC026355.2 and AL096701.4). ts showed close alignment with prior bioinformatics analysis. In particular, AL162632.3 was upregulated in all selected lung cancer cell lines, with the most notable expression in the H1299 cell line. The high expression of AL162632.3 may promote malignant behaviors in lung cancer cells. In contrast, AC026355.2 showed marked downregulation in the selected lung cancer cell lines, suggesting its potential anticancer activity. The downregulation of AC026355.2 may lead to a weakened response to growth-inhibiting signals in lung cancer cells, thereby aiding the progression of lung cancer. Hence, reinstating the expression of AC026355.2 could suppress lung cancer cells, providing a potential therapeutic target for lung cancer.

RT-qPCR analysis confirmed significant overexpression of lnc-AL162632.3 in lung cancer cell lines. To investigate its biological role, we transiently knocked down lnc-AL162632.3 in H1299 and A549 cells using RNAi technology. Knockdown significantly reduced cell proliferation, as shown by CCK8 and colony formation assays, and impaired migration and invasion in wound healing and invasion assays. *In vivo*, subcutaneous tumor models in nude mice revealed that tumors in the knockdown group were significantly smaller in volume and weight compared to controls. These findings indicate that lnc-AL162632.3 overexpression promotes lung cancer progression. To elucidate its regulatory mechanisms, future research will employ transcriptome sequencing to identify potential targets, such as GSH metabolism-related enzymes (e.g., GCLC, GSS) or signaling pathways influencing the tumor immune microenvironment. Additionally, RNA immunoprecipitation (RIP), chromatin immunoprecipitation (ChIP), and luciferase reporter assays will be used to validate these regulatory mechanisms.

This study highlights the role of GSH metabolism-related lncRNAs in LUAD while acknowledging certain limitations. The mechanisms by which lncRNAs regulate GSH metabolism remain unclear. The lack of suitable lncRNA probes has limited external validation, which we plan to address with RNA-seq in clinical samples. While LASSO-Cox was used for feature selection, future studies with larger samples should compare other methods like random forests. The nomogram predicts LUAD prognosis effectively but faces challenges such as individual variability in treatment response and lncRNA detection feasibility in clinical settings.

## 5 Conclusions

This study demonstrated the prognostic importance of GSH metabolism-related lncRNAs in lung adenocarcinoma and developed a risk stratification model, which was further integrated into a nomogram for enhanced clinical applicability. High-risk patients were characterized by increased TMB and stemness, suggesting a strong link between gene mutations and patient outcomes. These findings underscore the potential of the model and nomogram to guide personalized immunotherapy strategies and improve survival in lung adenocarcinoma.

## Data Availability

The original contributions presented in the study are included in the article/**Supplementary Material**. Further inquiries can be directed to the corresponding author/s.
