# Supplementary material for: A prognostic signature of Glutathione metabolism-associated long non-coding RNAs for lung adenocarcinoma with immune microenvironment insights
Source: Front Immunol. 2025 Feb 10;16:1477437. doi: 10.3389/fimmu.2025.1477437 (PMC11847877; doi:10.3389/fimmu.2025.1477437)
Supplement: Supplementary file 1 [file Table1.docx]

Supplementary Table 1 The sequences of primer, siRNA

primer

| AL162632.3-1F | AGTTTGGGCTCCCTGGAAAG |
| --- | --- |
| AL162632.3-1R | ACCACAGACATAGCCAACCC |
| AL078590.2-1F | CTTCTCCACAACCTGGCCTA |
| AL078590.2-1R | AGTCAGTGCTCCTTTGGACC |
| AC026355.2-1F | GGCCATTGCATCAACAGCAG |
| AC026355.2-1R | TGGACAGGGACCATTGTGAC |
| AL360270.1-1F | ATGTCTGGATATCTGGCCACTC |
| AL360270.1-1R | AGCAGGGGAAACAGCAAACTTA |
| AL096701.4-1F | GTAACTGCATCCTGGGGAGG |
| AL096701.4-1R | GTATCGCTTCACACCAACGC |
| LINC00707-1F | CCTTCGGCCCATTTCTCACT |
| LINC00707-1R | ATCACGGTGGCAGTATGGTG |
| DEPDC1.AS1-1F | GGAGAGGCATTCCTGTAGGC |
| DEPDC1.AS1-1R | TCAGAAGCCCAGTGTCCAAG |
| GSEC-1F | TGCTGAGAAAACCCACCCTG |
| GSEC-1R | GGTTGGGTGAGTCATGGTCC |
| LINC01711-1F | GGTCTGGAGCCGTTTCTCTC |
| LINC01711-1R | ATCCATCCTTGACCCTCGGA |

siRNA

| lnc-AL162632.3 siNC | Sense: 5’-GTATTCATTGCAGATTCATGT-3’ |
| --- | --- |
|  | Anti-sense:5’-TTCGGCAGCTATCGAGCCAC-3’ |
| lnc-AL162632.3 siRNA1 | Sense:5’- GCAGCTTCCTCACAGGCAATT -3’ |
|  | Anti-sense:5’-TTCTCCGAACAGCTCACGTTT-3’ |
| lnc-AL162632.3 siRNA2 | Sense: 5’- GGCAGCTGGAGCACAGATACC-3’ |
|  | Anti-sense: 5’- TATCTGTGCAGCTCTCGCCTG-3’ |
| lnc-AL162632.3 siRNA3 | Sense：5′- GGACAGCTCCAGCTAAGTG -3′, |
|  | Anti-sense： 5′-TACCAGCTTTCGATCGTTT-3′ |

Supplementary Table 2 Demographic and clinical characteristics between the training and testing cohort

| Covariates | Type | Total | Test | Train | Pvalue |
| --- | --- | --- | --- | --- | --- |
| Age | <=65 | 239 (47.14%) | 67 (44.08%) | 172 (48.45%) | 0.4159 |
|  | >65 | 258 (50.89%) | 82 (53.95%) | 176 (49.58%) |  |
|  | unknown | 10 (1.97%) | 3 (1.97%) | 7 (1.97%) |  |
| Gender | FEMALE | 272 (53.65%) | 89 (58.55%) | 183 (51.55%) | 0.1765 |
|  | MALE | 235 (46.35%) | 63 (41.45%) | 172 (48.45%) |  |
| Stage | Stage I | 272 (53.65%) | 87 (57.24%) | 185 (52.11%) | 0.649 |
|  | Stage II | 120 (23.67%) | 31 (20.39%) | 89 (25.07%) |  |
|  | Stage III | 81 (15.98%) | 25 (16.45%) | 56 (15.77%) |  |
|  | Stage IV | 26 (5.13%) | 7 (4.61%) | 19 (5.35%) |  |
|  | unknown | 8 (1.58%) | 2 (1.32%) | 6 (1.69%) |  |
| T | T1 | 169 (33.33%) | 57 (37.5%) | 112 (31.55%) | 0.1894 |
|  | T2 | 271 (53.45%) | 78 (51.32%) | 193 (54.37%) |  |
|  | T3 | 45 (8.88%) | 14 (9.21%) | 31 (8.73%) |  |
|  | T4 | 19 (3.75%) | 2 (1.32%) | 17 (4.79%) |  |
|  | unknown | 3 (0.59%) | 1 (0.66%) | 2 (0.56%) |  |
| M | M0 | 338 (66.67%) | 100 (65.79%) | 238 (67.04%) | 1 |
|  | M1 | 25 (4.93%) | 7 (4.61%) | 18 (5.07%) |  |
|  | unknown | 144 (28.4%) | 45 (29.61%) | 99 (27.89%) |  |
|  | N0 | 327 (64.5%) | 95 (62.5%) | 232 (65.35%) | 0.8199 |
| N | N1 | 95 (18.74%) | 26 (17.11%) | 69 (19.44%) |  |
|  | N2 | 71 (14%) | 23 (15.13%) | 48 (13.52%) |  |
|  | N3 | 2 (0.39%) | 1 (0.66%) | 1 (0.28%) |  |
|  | unknown | 12 (2.37%) | 7 (4.61%) | 5 (1.41%) |  |

Supplementary Table 3 The GSH metabolism-related lncRNAs risk signature

| lncRNAs | coef |
| --- | --- |
| AL162632.3 | 1.251134 |
| AL078590.2 | -0.60198 |
| AL360270.1 | 0.27111 |
| LINC00707 | 0.24617 |
| AC026355.2 | -0.34015 |
| DEPDC1-AS1 | 0.70375 |
| GSEC | 0.24977 |
| AL096701.4 | -0.42888 |
| LINC01711 | 0.26956 |

Supplementary Table 4 Risk score for patients in the training cohort

| TCGA id | AL162632.3 | AL078590.2 | AL360270.1 | LINC00707 | AC026355.2 | DEPDC1-AS1 | GSEC | AL096701.4 | LINC01711 | Score | risk |
| --- | --- | --- | --- | --- | --- | --- | --- | --- | --- | --- | --- |
| 35-5375 | 0.10 | 0.17 | 0.14 | 0.02 | 1.37 | 0.13 | 2.89 | 1.22 | 0.44 | 0.94 | low |
| 55-A4DF | 0.46 | 0.20 | 0.61 | 0.07 | 1.12 | 0.24 | 3.31 | 1.60 | 0.48 | 1.88 | high |
| 95-8039 | 0.40 | 0.93 | 0.76 | 0.30 | 1.41 | 0.15 | 1.84 | 1.01 | 1.17 | 1.12 | high |
| MP-A4T4 | 0.29 | 0.44 | 0.81 | 0.08 | 1.08 | 0.13 | 1.59 | 1.87 | 0.90 | 0.84 | low |
| 62-A471 | 0.06 | 0.15 | 0.27 | 3.77 | 2.00 | 0.08 | 4.15 | 1.15 | 0.85 | 2.92 | high |
| L9-A5IP | 0.00 | 0.28 | 1.78 | 3.02 | 1.11 | 0.00 | 5.09 | 0.44 | 1.80 | 8.85 | high |
| 50-5936 | 0.31 | 0.16 | 1.73 | 2.21 | 0.74 | 0.00 | 3.35 | 1.03 | 1.31 | 5.69 | high |
| 44-7659 | 0.57 | 0.41 | 1.23 | 0.07 | 2.85 | 0.00 | 0.89 | 2.00 | 0.44 | 0.48 | low |
| L9-A50W | 0.10 | 0.37 | 1.76 | 0.02 | 2.21 | 0.13 | 1.49 | 2.23 | 0.87 | 0.50 | low |
| 75-7025 | 0.30 | 0.80 | 0.80 | 0.64 | 1.77 | 0.00 | 1.66 | 2.03 | 0.48 | 0.48 | low |
| 05-4424 | 0.15 | 0.37 | 1.04 | 0.01 | 2.82 | 0.00 | 1.85 | 1.08 | 0.63 | 0.54 | low |
| 62-A46P | 0.08 | 0.22 | 1.05 | 0.04 | 0.57 | 0.00 | 1.77 | 1.93 | 0.75 | 0.84 | low |
| 86-8073 | 0.05 | 0.62 | 3.46 | 0.64 | 2.75 | 0.06 | 3.09 | 1.05 | 0.85 | 1.45 | high |
| 69-7765 | 0.38 | 0.46 | 2.62 | 0.04 | 1.80 | 0.09 | 1.55 | 1.20 | 0.81 | 1.47 | high |
| 44-5645 | 0.81 | 1.20 | 1.81 | 0.96 | 5.23 | 0.11 | 2.26 | 1.89 | 0.33 | 0.40 | low |
| 55-6984 | 0.16 | 0.17 | 1.04 | 0.14 | 0.40 | 0.00 | 0.98 | 0.80 | 0.90 | 1.42 | high |
| 69-7764 | 0.61 | 0.32 | 1.00 | 0.07 | 0.85 | 0.20 | 1.34 | 0.82 | 0.13 | 1.94 | high |
| NJ-A4YI | 0.00 | 0.26 | 1.95 | 0.00 | 0.97 | 0.13 | 1.84 | 1.62 | 1.88 | 1.40 | high |
| 86-A4P7 | 0.17 | 0.88 | 0.84 | 0.05 | 2.32 | 0.00 | 1.34 | 1.69 | 0.10 | 0.27 | low |
| 55-8620 | 0.08 | 0.25 | 0.83 | 0.01 | 0.20 | 0.10 | 2.14 | 3.75 | 0.44 | 0.43 | low |
| 97-8177 | 0.22 | 0.84 | 0.81 | 0.05 | 2.04 | 0.10 | 1.94 | 1.15 | 1.10 | 0.67 | low |
| 55-A492 | 0.20 | 0.66 | 0.54 | 0.91 | 3.83 | 0.00 | 2.10 | 2.29 | 0.00 | 0.20 | low |
| 78-7633 | 0.42 | 0.30 | 0.37 | 0.83 | 0.89 | 0.10 | 1.75 | 0.70 | 0.07 | 1.65 | high |
| 78-7220 | 0.08 | 0.55 | 2.52 | 0.00 | 0.93 | 0.28 | 2.38 | 0.77 | 0.71 | 2.05 | high |
| 53-7624 | 0.21 | 0.10 | 2.44 | 0.76 | 0.19 | 0.34 | 4.25 | 1.28 | 0.52 | 6.06 | high |
| 62-A46R | 0.63 | 1.16 | 1.78 | 0.04 | 0.16 | 0.22 | 1.53 | 1.72 | 1.00 | 1.71 | high |
| 91-8496 | 0.08 | 0.88 | 0.43 | 0.05 | 2.23 | 0.10 | 1.68 | 1.16 | 0.00 | 0.32 | low |
| 55-8092 | 0.34 | 0.25 | 1.47 | 0.31 | 0.90 | 0.08 | 2.69 | 1.67 | 0.77 | 1.81 | high |
| NJ-A4YQ | 0.22 | 0.50 | 2.49 | 0.13 | 2.53 | 0.15 | 3.77 | 1.72 | 0.87 | 1.35 | high |
| 91-6831 | 0.16 | 0.25 | 1.24 | 0.08 | 1.11 | 0.55 | 1.57 | 3.19 | 1.07 | 0.72 | low |
| 91-A4BD | 0.00 | 0.98 | 0.80 | 0.03 | 1.03 | 0.00 | 0.58 | 2.17 | 0.12 | 0.21 | low |
| 69-8253 | 0.07 | 0.38 | 1.89 | 0.14 | 1.62 | 0.00 | 1.39 | 0.97 | 0.00 | 0.75 | low |
| 78-8640 | 0.00 | 0.12 | 2.29 | 0.01 | 3.46 | 0.09 | 2.97 | 1.46 | 0.50 | 0.68 | low |
| 97-8174 | 0.11 | 0.97 | 0.80 | 0.07 | 1.24 | 0.05 | 1.64 | 1.51 | 0.84 | 0.50 | low |
| 55-6987 | 0.10 | 0.25 | 1.00 | 0.09 | 2.93 | 0.00 | 1.77 | 0.96 | 0.27 | 0.50 | low |
| 49-6744 | 0.12 | 0.56 | 0.77 | 0.11 | 1.64 | 0.00 | 1.28 | 1.13 | 0.78 | 0.59 | low |
| 55-8096 | 0.28 | 0.50 | 1.55 | 0.11 | 0.49 | 0.00 | 1.71 | 1.92 | 2.24 | 1.61 | high |
| J2-8192 | 0.64 | 1.44 | 1.44 | 0.06 | 2.37 | 0.16 | 1.80 | 1.65 | 1.32 | 0.72 | low |
| 44-7671 | 0.25 | 0.38 | 0.52 | 0.00 | 0.61 | 0.00 | 1.92 | 0.67 | 0.80 | 1.43 | high |
| 93-A4JN | 0.16 | 0.61 | 0.58 | 0.62 | 0.00 | 0.07 | 2.60 | 2.61 | 2.29 | 1.32 | high |
| 49-AAR9 | 0.38 | 0.10 | 2.33 | 0.65 | 0.00 | 0.18 | 2.23 | 0.36 | 1.51 | 7.97 | high |
| 86-A4P8 | 0.00 | 1.97 | 0.61 | 0.06 | 0.90 | 0.00 | 1.66 | 1.93 | 0.10 | 0.17 | low |
| 78-7158 | 0.23 | 0.48 | 0.98 | 0.01 | 1.80 | 0.22 | 2.30 | 1.94 | 0.44 | 0.67 | low |
| 80-5611 | 0.17 | 1.07 | 1.03 | 0.01 | 1.81 | 0.40 | 2.95 | 2.37 | 0.94 | 0.55 | low |
| 44-4112 | 0.48 | 0.90 | 1.92 | 0.25 | 5.81 | 0.06 | 1.63 | 0.56 | 0.50 | 0.35 | low |
| 71-6725 | 0.29 | 0.30 | 1.71 | 0.00 | 3.44 | 0.00 | 1.48 | 1.46 | 0.41 | 0.48 | low |
| 44-2656 | 0.75 | 1.93 | 2.35 | 0.18 | 4.73 | 0.23 | 2.40 | 1.00 | 0.55 | 0.48 | low |
| 05-5715 | 0.00 | 0.58 | 1.14 | 0.03 | 1.14 | 0.36 | 1.52 | 0.62 | 1.23 | 1.23 | high |
| 64-5815 | 0.00 | 0.57 | 0.99 | 0.11 | 1.67 | 0.00 | 1.54 | 0.34 | 1.82 | 1.05 | high |
| 55-7816 | 0.16 | 0.25 | 0.64 | 0.69 | 1.70 | 0.00 | 1.78 | 0.95 | 0.72 | 0.97 | high |
| 95-7948 | 0.19 | 0.28 | 0.70 | 0.15 | 3.11 | 0.00 | 1.43 | 3.15 | 0.72 | 0.20 | low |
| 05-4432 | 0.00 | 0.40 | 1.45 | 0.11 | 0.75 | 0.17 | 1.44 | 0.94 | 1.29 | 1.34 | high |
| 78-7143 | 0.26 | 0.49 | 1.86 | 0.55 | 1.31 | 0.25 | 2.74 | 1.30 | 0.80 | 1.96 | high |
| 73-4668 | 0.22 | 0.40 | 1.58 | 0.02 | 0.30 | 0.15 | 1.89 | 1.59 | 0.76 | 1.49 | high |
| 91-8499 | 0.00 | 0.92 | 0.35 | 0.03 | 0.00 | 0.43 | 2.26 | 2.36 | 0.54 | 0.60 | low |
| 86-6851 | 0.14 | 0.48 | 0.93 | 0.08 | 0.00 | 0.00 | 1.67 | 1.20 | 0.58 | 1.17 | high |
| 44-6779 | 0.23 | 0.31 | 2.05 | 1.59 | 1.64 | 0.63 | 4.09 | 1.75 | 0.51 | 3.56 | high |
| 78-7537 | 0.11 | 0.34 | 1.25 | 0.35 | 1.26 | 0.00 | 1.19 | 0.99 | 0.66 | 0.92 | low |
| 05-4434 | 0.55 | 1.16 | 0.92 | 1.11 | 1.06 | 0.36 | 4.01 | 1.23 | 0.91 | 2.87 | high |
| 67-3773 | 0.00 | 1.34 | 0.92 | 0.00 | 3.80 | 0.00 | 1.26 | 1.26 | 0.54 | 0.14 | low |
| 55-6983 | 0.23 | 0.21 | 1.00 | 0.05 | 0.98 | 0.30 | 3.13 | 1.28 | 0.53 | 1.88 | high |
| 05-5425 | 0.07 | 0.58 | 0.66 | 0.25 | 0.97 | 0.09 | 1.91 | 0.87 | 0.32 | 0.85 | low |
| 97-A4M2 | 0.05 | 0.95 | 0.64 | 0.12 | 2.56 | 0.07 | 1.86 | 1.93 | 0.25 | 0.22 | low |
| 50-5941 | 0.08 | 0.51 | 0.74 | 0.01 | 0.56 | 0.20 | 1.41 | 1.11 | 0.25 | 0.83 | low |
| 78-7539 | 0.25 | 0.41 | 0.40 | 0.01 | 1.25 | 0.00 | 1.29 | 0.99 | 0.21 | 0.69 | low |
| 67-3771 | 0.08 | 0.27 | 1.87 | 0.21 | 2.87 | 0.20 | 2.95 | 2.60 | 0.47 | 0.52 | low |
| 55-8299 | 0.11 | 0.63 | 1.13 | 1.16 | 1.36 | 0.14 | 3.78 | 1.41 | 1.36 | 1.88 | high |
| 86-A4JF | 0.16 | 0.27 | 0.51 | 0.00 | 2.79 | 0.07 | 1.14 | 1.36 | 0.09 | 0.34 | low |
| 05-4402 | 0.22 | 0.40 | 0.98 | 0.15 | 1.81 | 0.15 | 2.07 | 0.90 | 0.71 | 1.09 | high |
| 55-7914 | 0.06 | 0.34 | 1.58 | 0.04 | 0.99 | 0.16 | 2.39 | 1.11 | 1.82 | 1.87 | high |
| 49-4490 | 0.28 | 0.39 | 0.84 | 0.12 | 1.00 | 0.00 | 1.97 | 1.14 | 1.06 | 1.29 | high |
| 64-1681 | 0.29 | 0.52 | 1.21 | 0.02 | 0.98 | 0.06 | 1.57 | 1.24 | 0.34 | 0.98 | high |
| 99-AA5R | 0.06 | 1.09 | 0.58 | 0.07 | 1.36 | 0.00 | 1.70 | 2.18 | 0.42 | 0.26 | low |
| 49-4486 | 0.57 | 0.28 | 0.51 | 1.87 | 2.91 | 0.00 | 2.26 | 0.74 | 0.00 | 1.40 | high |
| 91-A4BC | 0.05 | 0.21 | 0.51 | 0.28 | 0.76 | 0.00 | 2.80 | 1.93 | 0.68 | 0.88 | low |
| 86-8672 | 0.39 | 0.15 | 1.72 | 0.30 | 0.60 | 0.32 | 4.42 | 0.66 | 1.31 | 7.84 | high |
| 50-5066 | 0.29 | 0.47 | 1.00 | 0.04 | 1.73 | 0.35 | 2.93 | 1.56 | 1.67 | 1.58 | high |
| 55-7727 | 0.44 | 0.71 | 1.27 | 0.02 | 3.69 | 0.00 | 1.42 | 1.81 | 0.00 | 0.28 | low |
| 50-5942 | 0.35 | 0.67 | 1.24 | 0.00 | 0.38 | 0.10 | 1.01 | 1.56 | 0.00 | 0.84 | low |
| 69-A59K | 0.15 | 0.70 | 0.90 | 0.11 | 2.35 | 0.37 | 1.63 | 1.41 | 0.64 | 0.55 | low |
| 44-2666 | 0.88 | 1.26 | 1.51 | 0.13 | 2.90 | 0.00 | 3.02 | 1.88 | 0.12 | 0.75 | low |
| 50-5944 | 0.24 | 0.49 | 1.33 | 0.01 | 0.87 | 0.11 | 1.52 | 1.83 | 0.26 | 0.78 | low |
| 55-6975 | 0.20 | 0.35 | 1.72 | 3.24 | 0.71 | 0.48 | 3.32 | 0.51 | 1.78 | 11.37 | high |
| 49-6767 | 0.09 | 0.21 | 1.68 | 0.01 | 0.43 | 0.79 | 2.40 | 0.86 | 2.74 | 5.79 | high |
| 44-A47G | 0.17 | 1.01 | 0.97 | 0.09 | 1.55 | 0.00 | 1.56 | 1.20 | 0.35 | 0.48 | low |
| NJ-A4YG | 0.31 | 1.00 | 0.45 | 0.15 | 1.38 | 0.09 | 1.39 | 1.81 | 1.71 | 0.61 | low |
| 55-8507 | 0.07 | 0.46 | 1.48 | 0.00 | 1.39 | 0.10 | 2.32 | 1.80 | 1.33 | 0.91 | low |
| 05-4433 | 0.10 | 1.32 | 1.06 | 0.10 | 0.48 | 0.00 | 1.13 | 0.85 | 0.00 | 0.51 | low |
| 55-A4DG | 0.62 | 0.63 | 1.08 | 0.06 | 1.70 | 0.00 | 2.65 | 2.18 | 0.38 | 0.90 | low |
| 49-4494 | 0.33 | 0.17 | 0.57 | 0.01 | 1.07 | 0.17 | 1.51 | 1.00 | 0.60 | 1.29 | high |
| NJ-A7XG | 0.29 | 0.38 | 0.57 | 0.05 | 2.55 | 0.00 | 2.19 | 2.48 | 0.20 | 0.33 | low |
| 49-AARO | 0.46 | 0.88 | 1.43 | 0.06 | 2.05 | 0.09 | 2.13 | 1.99 | 3.01 | 1.26 | high |
| 44-6774 | 0.00 | 0.36 | 0.62 | 0.06 | 1.86 | 0.00 | 1.73 | 0.57 | 1.86 | 0.96 | low |
| 49-AAR4 | 0.00 | 0.54 | 0.84 | 0.12 | 2.28 | 0.15 | 2.62 | 1.68 | 1.65 | 0.65 | low |
| 91-6848 | 0.00 | 0.47 | 0.54 | 0.32 | 0.30 | 0.40 | 2.29 | 0.82 | 2.16 | 2.36 | high |
| 44-2668 | 0.12 | 0.35 | 1.33 | 0.38 | 3.07 | 0.43 | 2.96 | 1.06 | 0.97 | 1.14 | high |
| 55-8091 | 0.05 | 0.69 | 0.84 | 0.11 | 1.23 | 0.12 | 1.32 | 1.61 | 1.86 | 0.69 | low |
| 50-6673 | 0.00 | 0.44 | 1.33 | 0.40 | 0.50 | 0.26 | 1.24 | 0.36 | 0.74 | 1.64 | high |
| 55-6642 | 0.11 | 0.29 | 0.44 | 0.18 | 0.82 | 0.00 | 1.39 | 0.53 | 0.90 | 1.15 | high |
| 50-6591 | 0.19 | 0.50 | 0.92 | 0.02 | 0.26 | 0.25 | 2.18 | 0.62 | 1.43 | 2.41 | high |
| 78-7166 | 0.19 | 0.10 | 0.73 | 0.25 | 0.47 | 0.04 | 3.53 | 1.26 | 0.71 | 2.14 | high |
| 05-4427 | 0.17 | 0.63 | 1.39 | 0.04 | 1.34 | 0.22 | 1.67 | 1.10 | 0.00 | 0.81 | low |
| 73-4676 | 0.14 | 0.54 | 0.61 | 0.00 | 3.70 | 0.18 | 3.68 | 1.28 | 0.00 | 0.44 | low |
| 49-4507 | 0.53 | 0.17 | 1.24 | 1.95 | 1.00 | 0.76 | 4.53 | 0.99 | 1.40 | 13.27 | high |
| 78-7148 | 0.13 | 0.18 | 1.31 | 0.66 | 0.59 | 0.08 | 3.04 | 0.42 | 0.21 | 2.70 | high |
| 93-A4JQ | 0.60 | 0.50 | 1.18 | 0.08 | 2.63 | 0.27 | 1.93 | 1.85 | 2.37 | 1.42 | high |
| 55-6969 | 0.00 | 0.37 | 0.69 | 0.16 | 0.38 | 0.04 | 1.26 | 1.60 | 1.19 | 0.80 | low |
| 93-7347 | 0.05 | 0.70 | 1.07 | 0.15 | 2.04 | 0.06 | 1.99 | 0.96 | 1.14 | 0.70 | low |
| 69-7978 | 0.09 | 1.11 | 0.98 | 0.11 | 2.53 | 0.22 | 1.71 | 0.91 | 1.14 | 0.50 | low |
| NJ-A4YF | 0.05 | 0.10 | 0.67 | 0.05 | 2.00 | 0.07 | 0.56 | 2.18 | 0.83 | 0.34 | low |
| 44-7672 | 0.22 | 0.31 | 0.47 | 0.08 | 1.46 | 0.13 | 1.12 | 0.76 | 1.39 | 1.09 | high |
| 78-7540 | 0.00 | 0.45 | 1.84 | 0.01 | 0.37 | 0.05 | 0.48 | 0.31 | 0.12 | 1.10 | high |
| 64-1677 | 0.28 | 0.24 | 0.26 | 0.03 | 3.72 | 0.24 | 0.99 | 1.51 | 0.67 | 0.33 | low |
| 05-4403 | 0.03 | 0.65 | 0.84 | 0.08 | 0.37 | 0.15 | 1.15 | 0.43 | 0.91 | 1.17 | high |
| 44-6148 | 0.07 | 0.94 | 1.09 | 0.30 | 0.36 | 0.18 | 2.06 | 1.80 | 0.00 | 0.65 | low |
| 55-6980 | 0.21 | 0.63 | 0.78 | 0.21 | 1.87 | 0.27 | 1.62 | 1.13 | 1.78 | 1.04 | high |
| 55-8511 | 0.00 | 0.36 | 0.88 | 0.11 | 1.66 | 0.00 | 2.45 | 1.19 | 1.26 | 0.87 | low |
| 78-7167 | 0.47 | 0.34 | 1.29 | 0.02 | 3.17 | 0.11 | 1.93 | 1.26 | 0.49 | 0.77 | low |
| 55-8615 | 0.06 | 0.13 | 1.10 | 0.00 | 0.81 | 0.00 | 1.68 | 1.55 | 0.99 | 0.98 | high |
| 55-A494 | 0.31 | 0.10 | 1.13 | 0.02 | 0.84 | 0.25 | 2.35 | 1.69 | 0.21 | 1.46 | high |
| 78-7147 | 0.21 | 0.29 | 1.37 | 0.32 | 0.40 | 0.49 | 2.34 | 1.78 | 0.47 | 1.86 | high |
| 97-A4M0 | 0.56 | 0.68 | 0.83 | 0.11 | 1.43 | 0.00 | 2.45 | 1.32 | 0.21 | 1.09 | high |
| 64-1679 | 0.04 | 0.56 | 1.76 | 0.02 | 1.19 | 0.06 | 1.95 | 0.43 | 1.65 | 1.67 | high |
| 64-1680 | 0.09 | 0.56 | 1.10 | 0.00 | 2.81 | 0.12 | 1.66 | 0.67 | 0.00 | 0.47 | low |
| 86-8669 | 0.16 | 0.36 | 0.58 | 0.07 | 1.32 | 0.11 | 1.41 | 1.36 | 0.27 | 0.65 | low |
| 78-7145 | 0.31 | 0.33 | 1.59 | 0.00 | 1.74 | 0.48 | 1.42 | 0.29 | 1.08 | 2.26 | high |
| 86-8075 | 0.13 | 0.44 | 1.30 | 0.18 | 1.86 | 0.11 | 2.05 | 0.61 | 1.89 | 1.54 | high |
| L9-A743 | 0.05 | 0.78 | 0.93 | 0.05 | 1.19 | 0.54 | 1.67 | 1.29 | 0.78 | 0.86 | low |
| 86-8054 | 0.00 | 0.19 | 1.31 | 0.01 | 0.62 | 0.27 | 1.11 | 0.91 | 0.41 | 1.16 | high |
| 62-A470 | 0.00 | 0.50 | 1.52 | 0.09 | 0.85 | 0.17 | 2.02 | 1.39 | 0.22 | 0.87 | low |
| 86-A456 | 0.08 | 1.29 | 1.26 | 0.04 | 1.90 | 0.10 | 2.16 | 1.60 | 0.00 | 0.33 | low |
| MP-A4SW | 0.10 | 0.64 | 0.76 | 0.03 | 1.97 | 0.13 | 1.80 | 1.60 | 0.71 | 0.48 | low |
| 44-3919 | 0.08 | 0.59 | 0.88 | 0.02 | 1.83 | 0.05 | 1.73 | 1.12 | 0.79 | 0.60 | low |
| 69-7973 | 0.42 | 0.27 | 1.10 | 0.03 | 0.60 | 0.21 | 2.45 | 1.44 | 0.50 | 1.96 | high |
| 86-8671 | 0.20 | 0.60 | 0.77 | 0.05 | 1.52 | 0.00 | 1.61 | 1.18 | 0.92 | 0.71 | low |
| 67-3772 | 0.18 | 0.75 | 0.76 | 0.10 | 2.09 | 0.00 | 1.58 | 0.76 | 0.89 | 0.63 | low |
| 35-4123 | 0.33 | 0.39 | 0.75 | 0.03 | 1.01 | 0.56 | 3.35 | 0.42 | 1.34 | 4.00 | high |
| 49-6761 | 0.08 | 0.15 | 1.78 | 0.01 | 1.49 | 0.11 | 1.39 | 0.53 | 0.68 | 1.35 | high |
| 44-A4SU | 0.14 | 0.27 | 1.02 | 0.08 | 1.61 | 0.29 | 1.93 | 2.95 | 1.71 | 0.65 | low |
| 05-4396 | 0.12 | 0.24 | 1.02 | 0.02 | 1.11 | 0.00 | 3.58 | 1.06 | 0.36 | 1.46 | high |
| 86-8358 | 0.00 | 0.36 | 0.67 | 0.04 | 0.28 | 0.39 | 2.43 | 2.54 | 1.37 | 0.97 | low |
| MN-A4N1 | 0.05 | 0.23 | 1.69 | 0.04 | 1.03 | 0.06 | 1.67 | 1.88 | 2.08 | 1.21 | high |
| 55-8301 | 0.12 | 0.29 | 0.72 | 0.12 | 1.71 | 0.05 | 1.45 | 1.31 | 0.53 | 0.63 | low |
| 49-4506 | 0.14 | 0.08 | 1.19 | 0.42 | 0.29 | 0.11 | 3.55 | 0.65 | 0.35 | 3.21 | high |
| 75-5146 | 0.09 | 0.92 | 0.57 | 0.10 | 1.94 | 0.22 | 2.19 | 1.21 | 0.00 | 0.45 | low |
| 62-A46O | 0.08 | 0.05 | 2.32 | 0.28 | 0.37 | 0.10 | 4.78 | 1.67 | 0.34 | 3.32 | high |
| 97-8552 | 0.07 | 1.02 | 0.49 | 0.08 | 1.15 | 0.00 | 1.41 | 2.09 | 0.22 | 0.27 | low |
| MP-A5C7 | 0.24 | 0.21 | 1.41 | 0.25 | 4.37 | 0.07 | 2.34 | 1.52 | 0.08 | 0.39 | low |
| 86-8074 | 0.60 | 0.58 | 2.46 | 0.02 | 2.23 | 0.00 | 2.18 | 1.02 | 2.94 | 3.16 | high |
| 97-7937 | 0.10 | 0.16 | 1.78 | 0.03 | 2.77 | 0.00 | 1.99 | 1.64 | 1.57 | 0.76 | low |
| 97-A4M7 | 0.05 | 0.87 | 0.47 | 1.55 | 1.05 | 0.06 | 1.70 | 2.30 | 0.77 | 0.50 | low |
| 05-4420 | 0.22 | 0.20 | 1.39 | 0.02 | 0.61 | 0.18 | 2.68 | 1.38 | 0.28 | 1.73 | high |
| 69-7979 | 0.08 | 0.19 | 1.04 | 0.03 | 1.22 | 0.11 | 2.07 | 1.90 | 0.54 | 0.76 | low |
| 78-7163 | 0.05 | 0.18 | 0.36 | 0.11 | 1.55 | 0.00 | 2.26 | 0.88 | 0.08 | 0.73 | low |
| 86-7701 | 0.30 | 0.50 | 1.46 | 0.05 | 0.51 | 0.27 | 1.55 | 1.37 | 0.93 | 1.62 | high |
| 38-4629 | 0.16 | 0.44 | 0.73 | 0.05 | 0.18 | 0.36 | 2.59 | 0.66 | 1.55 | 2.86 | high |
| 50-5931 | 0.10 | 0.31 | 1.50 | 1.74 | 0.27 | 0.66 | 3.09 | 3.89 | 1.52 | 1.82 | high |
| 55-7725 | 0.12 | 0.73 | 1.99 | 0.19 | 0.00 | 0.00 | 1.61 | 1.68 | 0.00 | 0.91 | low |
| MP-A4SY | 0.31 | 0.79 | 0.72 | 0.06 | 2.89 | 0.17 | 2.01 | 1.46 | 2.14 | 0.70 | low |
| 73-4659 | 0.14 | 0.93 | 0.42 | 0.03 | 1.42 | 0.21 | 2.16 | 1.26 | 0.56 | 0.60 | low |
| 69-7980 | 0.05 | 0.24 | 0.83 | 0.05 | 1.33 | 0.12 | 1.82 | 0.97 | 1.13 | 1.05 | high |
| 73-7498 | 0.35 | 0.79 | 0.83 | 0.03 | 3.04 | 0.00 | 1.77 | 1.69 | 0.19 | 0.32 | low |
| 86-8585 | 0.52 | 0.69 | 1.96 | 0.05 | 3.90 | 0.19 | 3.33 | 1.54 | 0.79 | 0.90 | low |
| 78-7542 | 0.48 | 0.23 | 1.62 | 0.01 | 0.50 | 0.96 | 1.41 | 0.59 | 3.41 | 10.57 | high |
| 44-6776 | 0.00 | 0.58 | 1.60 | 0.23 | 2.27 | 0.00 | 1.27 | 1.57 | 0.00 | 0.35 | low |
| 38-4630 | 0.71 | 1.08 | 0.47 | 0.04 | 0.10 | 1.17 | 2.42 | 1.93 | 0.82 | 2.96 | high |
| 05-4418 | 0.17 | 0.25 | 1.03 | 1.24 | 0.42 | 0.15 | 3.54 | 0.58 | 0.50 | 3.69 | high |
| 55-8085 | 0.26 | 0.28 | 0.97 | 0.08 | 1.71 | 0.24 | 1.73 | 2.18 | 0.51 | 0.67 | low |
| 55-7995 | 0.31 | 0.26 | 0.81 | 0.09 | 4.91 | 0.06 | 1.88 | 1.40 | 0.44 | 0.29 | low |
| 55-7284 | 0.09 | 1.47 | 1.93 | 0.04 | 0.12 | 0.12 | 1.24 | 0.73 | 1.05 | 1.01 | high |
| 55-6979 | 0.08 | 0.36 | 1.51 | 0.03 | 2.58 | 0.37 | 2.03 | 0.61 | 1.15 | 1.19 | high |
| 55-8204 | 0.04 | 0.26 | 0.61 | 0.41 | 0.32 | 0.21 | 1.72 | 0.87 | 1.10 | 1.62 | high |
| 62-8398 | 0.31 | 0.12 | 1.77 | 1.12 | 1.96 | 0.33 | 3.92 | 0.92 | 0.34 | 3.49 | high |
| MP-A4TI | 0.33 | 0.84 | 0.78 | 0.11 | 2.24 | 0.27 | 2.32 | 1.34 | 3.40 | 1.52 | high |
| 67-4679 | 0.14 | 1.89 | 0.63 | 0.00 | 2.81 | 0.09 | 1.06 | 1.88 | 0.00 | 0.10 | low |
| 05-4390 | 0.16 | 0.49 | 0.87 | 0.03 | 0.58 | 0.11 | 1.14 | 0.92 | 0.85 | 1.08 | high |
| 55-5899 | 0.43 | 1.25 | 0.88 | 0.00 | 0.21 | 0.10 | 1.63 | 1.26 | 0.57 | 0.97 | high |
| MP-A4TK | 0.31 | 0.77 | 1.00 | 0.07 | 2.64 | 0.00 | 1.74 | 1.73 | 2.99 | 0.78 | low |
| MN-A4N4 | 0.06 | 0.39 | 2.44 | 0.05 | 3.55 | 0.00 | 3.24 | 1.03 | 0.96 | 0.87 | low |
| 73-4666 | 0.30 | 0.47 | 0.92 | 0.05 | 1.41 | 0.46 | 3.12 | 0.89 | 0.31 | 1.83 | high |
| L4-A4E6 | 0.00 | 1.79 | 0.53 | 0.00 | 1.20 | 0.00 | 1.68 | 1.28 | 0.10 | 0.22 | low |
| 55-8207 | 0.37 | 1.02 | 1.69 | 0.02 | 1.71 | 0.17 | 1.58 | 1.23 | 0.78 | 0.88 | low |
| 78-7536 | 0.57 | 0.07 | 1.48 | 0.01 | 1.41 | 0.15 | 2.28 | 1.55 | 0.31 | 1.87 | high |
| 05-4389 | 0.21 | 0.62 | 0.45 | 0.03 | 1.91 | 0.11 | 2.43 | 1.15 | 0.14 | 0.63 | low |
| 97-7547 | 0.00 | 0.27 | 0.94 | 0.25 | 0.53 | 0.00 | 2.82 | 1.24 | 0.09 | 1.11 | high |
| 62-8394 | 0.64 | 0.30 | 1.25 | 0.03 | 0.67 | 0.15 | 2.59 | 1.08 | 0.19 | 2.75 | high |
| 86-8668 | 0.20 | 0.55 | 1.50 | 0.07 | 0.27 | 0.00 | 1.41 | 1.82 | 1.01 | 1.03 | high |
| 50-5939 | 0.72 | 0.22 | 1.84 | 2.44 | 0.78 | 0.00 | 2.92 | 0.16 | 0.41 | 10.02 | high |
| 38-7271 | 0.22 | 0.44 | 1.03 | 0.03 | 1.06 | 0.10 | 1.37 | 1.24 | 0.13 | 0.81 | low |
| 49-AARQ | 0.00 | 1.93 | 0.26 | 0.06 | 2.26 | 0.58 | 2.08 | 3.03 | 0.62 | 0.12 | low |
| 49-AAR3 | 0.16 | 0.37 | 0.98 | 0.55 | 1.92 | 0.11 | 3.21 | 1.63 | 2.13 | 1.51 | high |
| 44-A479 | 0.00 | 0.21 | 1.34 | 0.11 | 0.15 | 0.07 | 1.45 | 2.04 | 1.16 | 1.00 | high |
| 49-4514 | 0.40 | 0.11 | 0.60 | 0.21 | 0.76 | 0.07 | 1.75 | 0.90 | 0.08 | 1.56 | high |
| 62-A46S | 0.20 | 0.53 | 0.51 | 0.14 | 1.27 | 0.00 | 2.38 | 1.15 | 0.31 | 0.81 | low |
| 55-A490 | 0.10 | 0.11 | 0.57 | 0.02 | 0.94 | 0.00 | 1.42 | 0.43 | 0.70 | 1.21 | high |
| 97-7938 | 0.09 | 0.47 | 2.06 | 0.02 | 1.60 | 0.00 | 1.96 | 0.70 | 0.42 | 1.09 | high |
| 55-8097 | 0.00 | 0.97 | 1.34 | 0.00 | 1.54 | 0.00 | 1.72 | 2.18 | 0.49 | 0.31 | low |
| 55-8208 | 0.04 | 0.79 | 0.63 | 0.12 | 1.72 | 0.05 | 1.59 | 1.63 | 0.76 | 0.39 | low |
| 69-7974 | 0.21 | 0.85 | 1.72 | 0.05 | 2.10 | 0.22 | 1.72 | 1.05 | 0.72 | 0.80 | low |
| 55-6968 | 0.38 | 0.36 | 1.73 | 0.07 | 0.19 | 0.26 | 2.20 | 1.01 | 0.94 | 3.18 | high |
| 44-3917 | 0.83 | 0.31 | 2.59 | 0.26 | 5.04 | 0.57 | 2.31 | 1.30 | 0.24 | 1.38 | high |
| 05-4415 | 0.48 | 0.28 | 2.49 | 5.62 | 0.35 | 0.41 | 2.64 | 0.64 | 0.42 | 22.01 | high |
| 44-6145 | 0.15 | 0.24 | 0.58 | 0.86 | 1.72 | 0.00 | 2.77 | 0.60 | 0.46 | 1.38 | high |
| 44-7667 | 0.32 | 0.12 | 0.99 | 0.00 | 0.26 | 0.45 | 1.61 | 2.11 | 0.44 | 1.44 | high |
| 78-7150 | 0.14 | 0.05 | 2.36 | 2.65 | 0.50 | 0.18 | 4.16 | 0.88 | 1.38 | 10.52 | high |
| 95-7947 | 0.37 | 0.40 | 1.40 | 0.11 | 0.38 | 0.19 | 2.21 | 1.47 | 0.17 | 1.71 | high |
| 86-7711 | 0.00 | 0.77 | 2.03 | 0.03 | 0.97 | 0.13 | 2.72 | 0.50 | 0.27 | 1.39 | high |
| 55-8621 | 0.12 | 0.97 | 0.47 | 0.06 | 0.88 | 0.08 | 1.44 | 1.63 | 1.06 | 0.52 | low |
| L9-A8F4 | 0.00 | 1.20 | 1.17 | 0.16 | 1.79 | 0.06 | 2.57 | 1.81 | 0.91 | 0.41 | low |
| 86-7953 | 0.10 | 0.42 | 2.31 | 0.01 | 1.85 | 0.25 | 1.28 | 0.62 | 1.03 | 1.36 | high |
| 38-4628 | 0.23 | 0.56 | 1.03 | 0.00 | 0.56 | 0.09 | 1.38 | 0.18 | 0.41 | 1.51 | high |
| 55-A48Y | 0.39 | 0.41 | 1.05 | 0.29 | 3.67 | 0.00 | 2.02 | 0.45 | 1.63 | 1.03 | high |
| MP-A4T9 | 0.12 | 0.89 | 1.00 | 0.05 | 2.13 | 0.31 | 2.63 | 1.85 | 4.32 | 1.42 | high |
| 91-6835 | 0.23 | 1.08 | 1.31 | 0.14 | 2.27 | 0.00 | 1.98 | 1.96 | 0.13 | 0.32 | low |
| 50-6590 | 0.37 | 0.41 | 1.14 | 0.67 | 0.86 | 0.15 | 1.76 | 0.95 | 3.67 | 4.30 | high |
| 95-7039 | 0.10 | 0.32 | 1.35 | 0.02 | 1.65 | 0.24 | 2.16 | 1.50 | 0.87 | 0.97 | low |
| 55-8206 | 0.16 | 1.17 | 1.09 | 0.20 | 0.22 | 0.00 | 1.43 | 1.91 | 0.77 | 0.57 | low |
| 44-7661 | 0.20 | 0.55 | 1.30 | 0.03 | 1.33 | 0.31 | 1.79 | 1.01 | 1.84 | 1.61 | high |
| 75-6206 | 0.37 | 0.47 | 0.77 | 0.55 | 1.14 | 0.00 | 1.35 | 0.86 | 0.17 | 1.08 | high |
| 55-8616 | 0.19 | 0.42 | 0.97 | 0.02 | 1.81 | 0.00 | 1.63 | 1.38 | 0.49 | 0.62 | low |
| 97-A4M3 | 0.18 | 0.34 | 0.73 | 0.41 | 2.09 | 0.08 | 2.28 | 0.41 | 0.85 | 1.24 | high |
| 97-8172 | 0.31 | 1.27 | 1.19 | 0.07 | 0.28 | 0.10 | 1.89 | 1.90 | 0.39 | 0.69 | low |
| 55-7907 | 0.18 | 0.43 | 1.81 | 0.03 | 1.95 | 0.23 | 1.48 | 0.88 | 1.14 | 1.20 | high |
| MP-A4SV | 0.66 | 0.48 | 0.79 | 0.18 | 2.37 | 0.08 | 2.12 | 1.69 | 0.74 | 0.98 | high |
| 55-7726 | 0.00 | 0.38 | 1.28 | 0.16 | 1.18 | 0.26 | 2.26 | 1.51 | 2.97 | 1.79 | high |
| 97-A4M6 | 0.07 | 0.42 | 0.41 | 0.65 | 2.30 | 0.52 | 1.83 | 2.12 | 1.23 | 0.60 | low |
| 86-7955 | 0.45 | 0.07 | 1.87 | 0.08 | 1.18 | 0.10 | 3.42 | 2.51 | 0.31 | 1.67 | high |
| L9-A443 | 0.50 | 0.45 | 1.88 | 0.01 | 1.72 | 0.00 | 1.80 | 2.10 | 1.85 | 1.30 | high |
| 53-7813 | 0.15 | 0.11 | 0.51 | 0.19 | 1.06 | 0.00 | 1.57 | 0.96 | 0.36 | 0.96 | low |
| 86-8056 | 0.23 | 1.18 | 1.49 | 0.11 | 2.03 | 0.06 | 1.81 | 1.77 | 0.30 | 0.39 | low |
| 78-8655 | 0.27 | 0.73 | 0.68 | 0.02 | 3.02 | 0.00 | 2.54 | 1.87 | 1.61 | 0.48 | low |
| 86-8055 | 0.38 | 0.51 | 1.17 | 0.04 | 1.78 | 0.23 | 2.13 | 1.21 | 2.14 | 1.79 | high |
| 05-4405 | 0.00 | 0.16 | 1.98 | 0.05 | 1.32 | 0.00 | 2.19 | 1.47 | 1.76 | 1.38 | high |
| 99-7458 | 0.28 | 0.56 | 1.06 | 0.03 | 0.58 | 0.19 | 2.05 | 1.18 | 0.93 | 1.54 | high |
| 55-1594 | 0.32 | 0.16 | 1.38 | 0.04 | 0.43 | 0.47 | 0.67 | 1.87 | 0.51 | 1.36 | high |
| 44-7669 | 0.10 | 0.48 | 0.98 | 0.05 | 0.26 | 0.19 | 2.50 | 1.72 | 0.39 | 1.09 | high |
| 86-6562 | 0.30 | 0.62 | 1.78 | 0.03 | 0.57 | 0.00 | 1.70 | 0.98 | 1.18 | 1.74 | high |
| 05-4398 | 0.08 | 0.54 | 1.21 | 0.02 | 1.67 | 0.04 | 2.79 | 1.76 | 1.47 | 0.85 | low |
| 78-7161 | 0.45 | 0.22 | 0.69 | 0.10 | 1.43 | 0.00 | 2.69 | 1.58 | 0.86 | 1.37 | high |
| 50-5933 | 0.32 | 0.70 | 0.99 | 0.04 | 1.25 | 0.40 | 2.55 | 0.30 | 1.76 | 2.81 | high |
| 55-7728 | 0.03 | 1.51 | 0.50 | 0.05 | 0.17 | 0.04 | 1.00 | 1.19 | 0.42 | 0.38 | low |
| 55-8087 | 0.18 | 0.69 | 1.73 | 0.04 | 1.20 | 0.00 | 1.92 | 2.10 | 0.54 | 0.63 | low |
| 91-7771 | 0.27 | 0.44 | 0.82 | 0.39 | 0.88 | 0.00 | 1.68 | 1.27 | 0.59 | 1.05 | high |
| 67-6216 | 0.14 | 0.78 | 1.01 | 0.22 | 2.17 | 0.00 | 2.06 | 1.47 | 0.12 | 0.43 | low |
| 95-A4VP | 0.00 | 0.25 | 0.53 | 0.02 | 1.52 | 0.23 | 1.34 | 2.25 | 0.80 | 0.43 | low |
| 73-4675 | 0.11 | 0.56 | 0.49 | 1.39 | 0.44 | 0.05 | 2.22 | 1.66 | 1.85 | 1.54 | high |
| 64-1676 | 0.18 | 0.63 | 0.70 | 0.01 | 3.09 | 0.16 | 2.34 | 0.42 | 0.47 | 0.65 | low |
| 75-5147 | 0.32 | 0.82 | 0.69 | 0.03 | 1.99 | 0.55 | 2.91 | 1.22 | 0.59 | 1.11 | high |
| L9-A7SV | 0.27 | 0.27 | 1.29 | 0.02 | 0.25 | 0.06 | 2.23 | 2.91 | 0.43 | 0.85 | low |
| 78-7160 | 0.17 | 0.90 | 1.56 | 0.83 | 1.53 | 0.22 | 3.91 | 0.55 | 0.27 | 1.97 | high |
| 55-1595 | 0.12 | 0.51 | 0.41 | 0.03 | 1.74 | 0.05 | 1.05 | 1.79 | 0.69 | 0.37 | low |
| 78-8648 | 0.07 | 0.89 | 0.14 | 0.14 | 0.49 | 0.00 | 1.38 | 0.71 | 1.44 | 0.84 | low |
| 55-A57B | 0.16 | 0.63 | 2.11 | 0.03 | 1.69 | 0.20 | 1.66 | 1.32 | 1.24 | 1.08 | high |
| 55-6986 | 0.19 | 0.42 | 0.88 | 0.02 | 1.58 | 0.00 | 1.31 | 1.05 | 0.16 | 0.64 | low |
| 91-6849 | 0.00 | 0.50 | 0.92 | 0.13 | 2.48 | 0.00 | 0.81 | 1.38 | 0.00 | 0.27 | low |
| 55-7903 | 0.46 | 0.36 | 1.50 | 0.01 | 0.47 | 0.00 | 1.61 | 1.41 | 0.31 | 1.52 | high |
| 86-8280 | 0.18 | 0.55 | 0.73 | 0.05 | 1.86 | 0.12 | 1.64 | 2.08 | 0.60 | 0.44 | low |
| 91-6828 | 0.35 | 0.88 | 0.59 | 0.06 | 0.32 | 0.31 | 1.52 | 1.95 | 0.20 | 0.75 | low |
| 50-5932 | 0.29 | 0.49 | 1.17 | 0.04 | 0.32 | 0.16 | 1.57 | 0.33 | 0.00 | 1.80 | high |
| NJ-A55O | 0.13 | 1.30 | 1.31 | 0.30 | 1.75 | 0.00 | 2.22 | 1.37 | 0.11 | 0.42 | low |
| 05-5423 | 0.38 | 1.58 | 0.76 | 0.00 | 3.10 | 0.11 | 2.11 | 1.63 | 0.85 | 0.28 | low |
| 05-5420 | 0.00 | 0.68 | 0.55 | 0.06 | 1.26 | 0.08 | 1.74 | 0.63 | 0.10 | 0.61 | low |
| 62-A46Y | 0.39 | 0.71 | 1.37 | 0.08 | 0.58 | 0.00 | 2.12 | 1.57 | 0.20 | 1.10 | high |
| 05-4384 | 0.34 | 0.92 | 0.76 | 0.67 | 4.55 | 0.12 | 2.86 | 1.76 | 0.00 | 0.27 | low |
| 97-A4M1 | 0.06 | 0.75 | 1.03 | 0.04 | 0.95 | 0.00 | 1.40 | 2.91 | 0.51 | 0.29 | low |
| 55-8619 | 0.00 | 0.60 | 0.53 | 0.60 | 0.53 | 0.00 | 1.52 | 0.98 | 0.09 | 0.71 | low |
| MN-A4N5 | 0.35 | 0.31 | 2.18 | 0.03 | 2.00 | 0.20 | 1.83 | 1.70 | 0.94 | 1.25 | high |
| 95-A4VK | 0.46 | 0.44 | 1.50 | 0.24 | 0.18 | 0.00 | 2.95 | 2.09 | 0.48 | 1.87 | high |
| 75-7027 | 0.07 | 0.27 | 1.31 | 0.13 | 1.07 | 0.27 | 2.67 | 0.76 | 1.69 | 2.39 | high |
| 64-5779 | 0.06 | 0.20 | 1.11 | 0.08 | 1.76 | 0.08 | 1.13 | 1.23 | 0.75 | 0.69 | low |
| 44-6775 | 0.83 | 1.83 | 2.12 | 0.30 | 4.00 | 0.20 | 2.25 | 1.29 | 1.66 | 0.77 | low |
| 44-2665 | 0.61 | 1.80 | 1.32 | 0.27 | 4.78 | 0.49 | 3.34 | 1.47 | 2.16 | 0.62 | low |
| 86-8359 | 0.68 | 0.06 | 1.81 | 0.03 | 0.54 | 0.00 | 2.59 | 0.59 | 0.94 | 5.51 | high |
| 44-3396 | 0.02 | 0.59 | 0.75 | 0.11 | 1.21 | 0.12 | 2.08 | 1.26 | 0.85 | 0.75 | low |
| J2-A4AG | 0.00 | 0.56 | 1.05 | 0.17 | 1.69 | 0.00 | 1.24 | 0.72 | 0.83 | 0.65 | low |
| 78-7152 | 0.12 | 0.13 | 1.23 | 0.16 | 2.15 | 0.00 | 2.06 | 0.58 | 0.53 | 1.06 | high |
| 93-7348 | 0.06 | 0.35 | 0.75 | 0.13 | 0.46 | 0.00 | 1.52 | 1.75 | 1.50 | 0.92 | low |
| 64-5775 | 0.00 | 1.21 | 1.12 | 0.16 | 0.20 | 1.38 | 2.95 | 0.58 | 3.03 | 5.76 | high |
| 78-7149 | 0.21 | 0.36 | 1.50 | 0.66 | 3.04 | 0.14 | 1.94 | 1.32 | 0.25 | 0.66 | low |
| 44-2661 | 0.04 | 1.20 | 0.68 | 0.08 | 0.67 | 0.16 | 1.79 | 1.46 | 0.27 | 0.47 | low |
| 73-4658 | 0.08 | 0.66 | 1.67 | 0.01 | 0.20 | 0.00 | 1.81 | 1.00 | 0.78 | 1.29 | high |
| 55-8205 | 0.33 | 0.81 | 0.73 | 0.08 | 0.38 | 0.25 | 2.06 | 0.86 | 0.32 | 1.42 | high |
| 55-8203 | 0.06 | 0.45 | 0.39 | 0.05 | 0.25 | 0.08 | 1.53 | 1.07 | 0.79 | 0.97 | high |
| 78-8660 | 0.00 | 0.59 | 1.29 | 0.04 | 1.20 | 0.35 | 1.34 | 1.41 | 0.23 | 0.65 | low |
| 38-4631 | 0.08 | 0.18 | 1.38 | 0.09 | 0.93 | 0.36 | 2.68 | 0.49 | 0.79 | 2.52 | high |
| 64-5778 | 0.22 | 0.47 | 0.68 | 0.06 | 1.18 | 0.00 | 1.97 | 2.28 | 0.10 | 0.48 | low |
| 55-6543 | 0.08 | 0.55 | 0.95 | 0.01 | 1.23 | 0.19 | 1.72 | 0.93 | 0.45 | 0.84 | low |
| 62-8399 | 0.06 | 0.56 | 2.94 | 0.03 | 5.13 | 0.08 | 2.71 | 1.35 | 0.19 | 0.34 | low |
| 55-7283 | 0.27 | 0.48 | 0.54 | 0.02 | 1.42 | 0.00 | 1.73 | 0.56 | 0.00 | 0.85 | low |
| 50-6593 | 0.25 | 0.34 | 0.96 | 0.06 | 0.60 | 0.22 | 2.57 | 1.19 | 1.24 | 2.06 | high |
| 50-8459 | 0.06 | 1.72 | 0.71 | 0.23 | 0.43 | 0.08 | 1.60 | 1.44 | 4.11 | 1.01 | high |
| 49-AAQV | 0.34 | 0.25 | 1.01 | 0.22 | 2.43 | 0.27 | 2.35 | 1.61 | 0.89 | 1.04 | high |
| 38-4626 | 0.03 | 0.55 | 0.56 | 0.03 | 1.81 | 0.11 | 1.31 | 1.14 | 0.05 | 0.41 | low |
| 95-7562 | 0.36 | 0.11 | 1.12 | 0.01 | 0.44 | 0.62 | 2.03 | 1.13 | 0.22 | 2.71 | high |
| 71-8520 | 0.20 | 0.48 | 1.44 | 0.00 | 1.27 | 0.36 | 2.01 | 2.09 | 1.98 | 1.27 | high |
| 55-8614 | 0.17 | 0.34 | 1.01 | 0.03 | 1.13 | 0.21 | 2.13 | 1.95 | 2.07 | 1.27 | high |
| 55-7574 | 0.19 | 0.98 | 1.16 | 0.15 | 1.22 | 0.00 | 1.82 | 1.41 | 1.72 | 0.84 | low |
| 97-7941 | 0.17 | 0.80 | 0.71 | 0.93 | 0.82 | 0.06 | 1.67 | 1.35 | 0.33 | 0.80 | low |
| 44-7660 | 0.05 | 0.59 | 1.13 | 0.17 | 0.62 | 0.22 | 4.50 | 3.36 | 1.27 | 0.95 | low |
| 55-6971 | 0.17 | 0.41 | 0.75 | 0.10 | 0.60 | 0.00 | 2.01 | 1.44 | 0.39 | 0.91 | low |
| 78-7155 | 0.00 | 0.11 | 1.19 | 0.03 | 0.26 | 0.00 | 1.86 | 1.14 | 0.57 | 1.27 | high |
| MP-A4TE | 0.09 | 0.13 | 0.74 | 0.02 | 0.54 | 0.06 | 2.41 | 0.98 | 0.29 | 1.34 | high |
| 95-A4VN | 0.34 | 0.54 | 0.41 | 0.01 | 2.25 | 0.08 | 2.21 | 1.04 | 0.67 | 0.75 | low |
| 93-8067 | 0.48 | 0.07 | 1.14 | 0.07 | 0.67 | 0.10 | 1.68 | 0.63 | 0.95 | 2.92 | high |
| J2-8194 | 0.15 | 0.39 | 1.28 | 0.02 | 2.18 | 0.07 | 2.32 | 1.53 | 0.32 | 0.64 | low |
| 55-8510 | 0.00 | 0.71 | 0.97 | 0.03 | 1.92 | 0.00 | 2.40 | 1.52 | 1.14 | 0.54 | low |
| 44-5643 | 0.25 | 0.52 | 1.00 | 0.03 | 2.87 | 0.00 | 0.98 | 1.53 | 0.15 | 0.32 | low |
| 86-7713 | 0.32 | 0.15 | 1.03 | 0.01 | 0.43 | 0.06 | 1.15 | 1.47 | 0.21 | 1.15 | high |
| NJ-A55R | 0.16 | 1.28 | 0.98 | 0.09 | 2.45 | 0.12 | 3.02 | 2.31 | 0.95 | 0.34 | low |
| 50-5044 | 0.09 | 0.41 | 0.17 | 0.96 | 0.89 | 0.65 | 2.12 | 0.66 | 3.97 | 4.68 | high |
| 64-5774 | 0.06 | 0.09 | 1.01 | 0.02 | 0.90 | 0.49 | 1.09 | 2.26 | 0.86 | 0.83 | low |
| 55-6712 | 1.09 | 0.56 | 2.58 | 0.42 | 0.00 | 0.00 | 1.63 | 0.61 | 0.67 | 8.04 | high |
| 4B-A93V | 0.00 | 0.11 | 0.46 | 0.36 | 0.90 | 0.00 | 1.19 | 2.59 | 0.82 | 0.44 | low |
| 83-5908 | 0.23 | 0.36 | 1.83 | 0.15 | 1.37 | 0.75 | 1.97 | 1.05 | 1.65 | 2.98 | high |
| 44-A4SS | 0.15 | 0.84 | 1.54 | 0.12 | 2.71 | 0.19 | 2.28 | 0.68 | 1.45 | 0.94 | low |
| 93-A4JP | 0.17 | 1.04 | 1.03 | 0.08 | 0.84 | 0.07 | 2.64 | 1.93 | 0.19 | 0.58 | low |
| 55-6978 | 0.16 | 0.71 | 1.48 | 0.11 | 1.13 | 0.38 | 1.57 | 0.66 | 1.16 | 1.55 | high |
| 78-7146 | 0.51 | 0.13 | 0.94 | 0.07 | 2.68 | 0.63 | 3.72 | 0.48 | 1.84 | 4.55 | high |
| MP-A4TD | 0.05 | 0.60 | 0.61 | 0.14 | 2.47 | 0.06 | 2.22 | 1.36 | 1.72 | 0.59 | low |
| 50-5930 | 0.00 | 0.27 | 1.17 | 0.84 | 0.54 | 0.00 | 2.59 | 0.21 | 0.63 | 2.31 | high |
| 50-7109 | 0.12 | 0.35 | 1.02 | 0.03 | 1.59 | 0.15 | 1.52 | 1.19 | 1.20 | 0.92 | low |
| 05-5428 | 0.24 | 0.94 | 1.21 | 0.00 | 2.24 | 0.11 | 3.42 | 0.65 | 0.14 | 0.93 | low |
| NJ-A4YP | 0.27 | 0.14 | 2.51 | 0.39 | 0.71 | 0.11 | 1.99 | 1.63 | 1.73 | 2.90 | high |
| 49-AAR2 | 0.19 | 0.26 | 0.70 | 0.08 | 1.61 | 0.19 | 1.60 | 1.81 | 0.76 | 0.69 | low |
| 50-5045 | 0.16 | 0.83 | 0.84 | 0.05 | 0.65 | 0.11 | 2.29 | 0.63 | 1.62 | 1.59 | high |
| 44-8119 | 0.13 | 0.39 | 0.77 | 0.06 | 0.46 | 0.08 | 1.87 | 1.43 | 0.58 | 0.99 | high |
| 44-8117 | 0.19 | 0.35 | 1.83 | 0.01 | 1.80 | 0.00 | 1.41 | 1.95 | 1.58 | 0.81 | low |
| 05-4425 | 0.39 | 0.32 | 0.99 | 0.00 | 0.23 | 0.00 | 1.69 | 2.01 | 0.88 | 1.25 | high |
| 91-6836 | 0.07 | 0.10 | 1.56 | 0.11 | 0.36 | 0.18 | 1.61 | 0.74 | 0.52 | 1.89 | high |
| 49-4488 | 0.10 | 0.38 | 1.23 | 0.02 | 2.55 | 0.24 | 1.61 | 1.36 | 0.31 | 0.53 | low |
| 99-8032 | 0.18 | 0.35 | 0.81 | 0.06 | 0.53 | 0.10 | 1.83 | 1.35 | 1.41 | 1.39 | high |
| 44-7662 | 0.15 | 0.56 | 1.34 | 0.02 | 0.46 | 0.19 | 3.00 | 1.74 | 1.84 | 1.87 | high |
| 44-2655 | 0.43 | 0.61 | 0.58 | 0.40 | 4.33 | 0.00 | 3.11 | 1.03 | 0.21 | 0.48 | low |
| 86-8673 | 0.07 | 0.15 | 1.16 | 0.26 | 3.15 | 0.09 | 1.95 | 1.73 | 1.00 | 0.50 | low |
| MP-A4TF | 0.44 | 0.52 | 2.36 | 1.25 | 2.30 | 0.13 | 1.53 | 1.04 | 2.56 | 2.85 | high |
| 55-7911 | 0.20 | 0.46 | 0.87 | 0.02 | 4.26 | 0.34 | 1.85 | 2.13 | 0.82 | 0.27 | low |
| 05-4244 | 0.43 | 0.36 | 0.86 | 0.02 | 1.64 | 0.18 | 0.88 | 1.87 | 0.30 | 0.64 | low |
| 73-4677 | 0.25 | 0.54 | 0.67 | 0.23 | 2.23 | 0.00 | 1.45 | 0.57 | 0.00 | 0.62 | low |
| 05-4426 | 0.38 | 0.23 | 1.12 | 0.08 | 1.20 | 0.28 | 1.35 | 1.37 | 0.59 | 1.34 | high |
| 97-8176 | 0.08 | 0.44 | 0.85 | 1.06 | 2.71 | 0.28 | 4.46 | 1.57 | 0.55 | 1.13 | high |
| 05-4382 | 0.03 | 0.88 | 1.23 | 0.19 | 3.74 | 0.14 | 1.89 | 1.03 | 1.11 | 0.36 | low |
| J2-A4AE | 0.18 | 0.75 | 0.77 | 0.12 | 1.68 | 0.00 | 1.41 | 1.21 | 0.10 | 0.46 | low |
| 73-4670 | 0.26 | 0.15 | 1.45 | 0.25 | 1.02 | 0.08 | 4.74 | 0.89 | 1.20 | 4.29 | high |
| 55-8506 | 0.16 | 0.38 | 3.04 | 0.01 | 0.84 | 0.00 | 1.92 | 1.59 | 0.36 | 1.40 | high |
| 50-6597 | 0.08 | 0.20 | 1.34 | 0.00 | 0.22 | 0.47 | 1.77 | 0.41 | 0.14 | 2.31 | high |
| 44-3398 | 0.13 | 1.24 | 0.48 | 0.05 | 3.04 | 0.27 | 2.02 | 1.35 | 2.30 | 0.45 | low |
| 73-A9RS | 0.12 | 0.48 | 2.12 | 0.15 | 0.95 | 0.29 | 3.61 | 1.60 | 0.36 | 1.81 | high |
| 53-7626 | 0.12 | 0.75 | 1.39 | 0.16 | 3.04 | 0.00 | 1.53 | 3.42 | 0.19 | 0.13 | low |
| 55-8505 | 0.15 | 0.21 | 1.93 | 3.08 | 1.43 | 0.28 | 3.61 | 1.10 | 1.96 | 7.02 | high |
| 97-A4M5 | 0.00 | 1.07 | 0.93 | 0.31 | 0.40 | 0.14 | 1.72 | 1.24 | 0.78 | 0.73 | low |
| 75-5125 | 0.08 | 0.81 | 1.07 | 0.03 | 3.48 | 0.60 | 2.15 | 1.59 | 0.96 | 0.45 | low |
| 62-A46U | 0.06 | 0.72 | 1.06 | 0.08 | 2.59 | 0.23 | 1.30 | 1.28 | 0.54 | 0.40 | low |
| MP-A4TJ | 0.08 | 0.64 | 1.06 | 0.25 | 0.22 | 0.00 | 1.85 | 1.37 | 0.86 | 1.03 | high |
| 50-5055 | 0.00 | 0.56 | 0.53 | 0.26 | 1.16 | 0.12 | 1.81 | 1.20 | 1.19 | 0.77 | low |
| 91-6829 | 0.09 | 0.46 | 2.39 | 0.06 | 1.29 | 0.12 | 2.67 | 0.98 | 1.52 | 2.08 | high |
| 49-AARR | 0.06 | 0.54 | 0.81 | 0.19 | 0.79 | 0.00 | 1.40 | 2.22 | 0.10 | 0.41 | low |
| 78-8662 | 0.61 | 0.16 | 0.06 | 0.00 | 2.15 | 0.12 | 3.08 | 3.29 | 0.53 | 0.59 | low |
| 05-4430 | 0.12 | 0.61 | 1.38 | 0.01 | 1.50 | 0.16 | 1.99 | 1.36 | 0.97 | 0.88 | low |
| 44-6146 | 0.65 | 0.66 | 2.14 | 0.12 | 3.67 | 0.09 | 1.76 | 1.24 | 0.29 | 0.78 | low |
| 38-4627 | 0.14 | 0.35 | 0.63 | 0.11 | 0.94 | 0.06 | 2.53 | 0.64 | 1.76 | 1.91 | high |
| 50-6595 | 0.19 | 0.17 | 1.48 | 0.73 | 0.47 | 0.24 | 2.06 | 0.33 | 1.74 | 4.47 | high |
| 97-A4LX | 0.53 | 1.02 | 1.14 | 0.09 | 0.74 | 0.40 | 1.57 | 1.53 | 0.69 | 1.30 | high |
| 49-AARN | 0.35 | 0.37 | 0.96 | 0.20 | 0.46 | 0.00 | 1.67 | 1.93 | 1.91 | 1.49 | high |
| 78-7156 | 1.09 | 0.38 | 1.37 | 1.53 | 4.16 | 0.10 | 0.93 | 1.30 | 0.00 | 1.15 | high |
| 50-8457 | 0.00 | 0.72 | 0.80 | 0.05 | 1.47 | 0.00 | 1.14 | 1.00 | 0.48 | 0.46 | low |
| 50-5072 | 0.97 | 0.84 | 1.47 | 2.13 | 1.43 | 0.70 | 3.83 | 1.48 | 0.29 | 7.18 | high |
| 69-8255 | 0.05 | 0.11 | 1.82 | 0.14 | 0.83 | 0.13 | 3.32 | 1.51 | 0.09 | 1.62 | high |
| 86-A4D0 | 0.42 | 0.30 | 0.07 | 0.03 | 0.28 | 0.63 | 2.87 | 1.83 | 0.82 | 2.25 | high |
| 44-2662 | 0.19 | 1.00 | 1.74 | 0.57 | 5.62 | 0.60 | 2.30 | 1.55 | 0.67 | 0.30 | low |
| 99-8033 | 0.15 | 0.16 | 1.36 | 2.21 | 3.27 | 0.15 | 4.42 | 2.16 | 0.92 | 1.44 | high |
| 44-5644 | 0.09 | 0.06 | 1.42 | 0.01 | 1.12 | 0.11 | 0.97 | 1.66 | 0.69 | 0.82 | low |
| 38-4625 | 0.00 | 0.49 | 0.53 | 0.04 | 0.63 | 0.36 | 2.23 | 1.59 | 0.29 | 0.80 | low |
| 05-5429 | 0.09 | 0.36 | 0.79 | 0.02 | 0.79 | 0.52 | 1.03 | 0.90 | 0.15 | 1.06 | high |
| 93-A4JO | 0.00 | 0.27 | 0.79 | 0.06 | 0.99 | 0.00 | 2.39 | 2.51 | 0.38 | 0.49 | low |
| 44-A47A | 0.15 | 1.09 | 0.54 | 0.01 | 4.22 | 0.36 | 2.43 | 2.44 | 0.87 | 0.17 | low |
| 97-8179 | 0.38 | 0.39 | 1.15 | 0.02 | 2.84 | 0.00 | 2.38 | 1.84 | 0.86 | 0.64 | low |
| 55-8090 | 0.21 | 0.31 | 1.17 | 0.03 | 1.47 | 0.10 | 2.59 | 1.09 | 1.43 | 1.60 | high |
| MP-A4TC | 0.00 | 0.07 | 1.00 | 0.21 | 2.85 | 0.24 | 2.53 | 1.31 | 2.63 | 1.21 | high |
| 91-6847 | 0.11 | 0.08 | 0.96 | 0.01 | 0.14 | 0.32 | 3.13 | 2.88 | 2.79 | 2.13 | high |
| 05-4249 | 0.10 | 0.75 | 0.86 | 0.26 | 1.23 | 0.00 | 1.77 | 0.47 | 0.30 | 0.82 | low |
| 55-7570 | 0.06 | 0.22 | 1.07 | 0.03 | 0.55 | 0.29 | 3.25 | 1.68 | 0.99 | 1.73 | high |
| 50-5049 | 0.05 | 0.61 | 0.75 | 0.04 | 1.87 | 0.00 | 1.95 | 0.81 | 0.95 | 0.67 | low |
| 86-8674 | 0.21 | 0.03 | 1.37 | 0.06 | 4.78 | 0.00 | 3.37 | 1.74 | 1.04 | 0.50 | low |
| 53-A4EZ | 0.00 | 0.09 | 0.61 | 0.05 | 1.47 | 0.11 | 2.23 | 2.35 | 0.14 | 0.46 | low |
| 69-7761 | 0.06 | 0.44 | 1.41 | 0.17 | 0.16 | 0.08 | 0.88 | 0.76 | 0.98 | 1.38 | high |
| 50-6592 | 0.12 | 0.21 | 0.81 | 0.72 | 0.45 | 0.08 | 1.22 | 1.20 | 0.97 | 1.36 | high |
| 62-8395 | 0.44 | 0.45 | 1.83 | 0.35 | 1.83 | 0.00 | 1.53 | 1.45 | 0.43 | 1.05 | high |
| 05-4410 | 0.00 | 0.44 | 1.47 | 0.04 | 0.76 | 0.22 | 2.00 | 0.00 | 0.28 | 1.72 | high |
| 73-4662 | 0.27 | 0.67 | 1.02 | 0.05 | 1.01 | 0.00 | 1.12 | 1.34 | 0.34 | 0.67 | low |
| 44-6147 | 0.59 | 0.79 | 2.40 | 0.13 | 5.18 | 0.00 | 1.85 | 1.47 | 0.59 | 0.41 | low |
| 50-8460 | 0.17 | 0.92 | 0.46 | 0.05 | 1.03 | 0.08 | 1.22 | 1.86 | 0.10 | 0.35 | low |
| 49-AARE | 0.37 | 0.10 | 2.12 | 0.68 | 0.64 | 0.05 | 2.06 | 1.68 | 0.87 | 2.51 | high |
| 55-6985 | 0.37 | 0.34 | 1.26 | 0.03 | 0.40 | 0.29 | 1.95 | 0.52 | 1.05 | 3.16 | high |
| 49-4487 | 0.51 | 0.28 | 0.88 | 0.41 | 0.74 | 0.40 | 1.53 | 0.93 | 1.28 | 2.98 | high |
| 55-8514 | 0.14 | 0.40 | 0.59 | 0.01 | 2.00 | 0.00 | 1.76 | 2.46 | 0.50 | 0.32 | low |
| 55-7576 | 0.00 | 0.58 | 1.02 | 0.01 | 0.55 | 0.46 | 1.84 | 0.62 | 0.36 | 1.33 | high |
| 91-6830 | 0.49 | 0.58 | 0.97 | 2.76 | 1.80 | 0.28 | 4.01 | 1.42 | 1.07 | 4.08 | high |
| 55-7227 | 0.35 | 0.55 | 1.28 | 0.01 | 1.14 | 0.00 | 1.53 | 1.74 | 0.72 | 0.84 | low |
| MP-A4T6 | 0.15 | 0.67 | 1.82 | 0.03 | 1.66 | 0.00 | 1.91 | 2.30 | 0.25 | 0.45 | low |
| 99-8028 | 0.14 | 0.78 | 0.64 | 0.08 | 0.82 | 0.14 | 1.68 | 1.33 | 0.95 | 0.78 | low |
| 86-8278 | 0.18 | 0.77 | 0.67 | 0.14 | 3.26 | 0.23 | 1.93 | 1.73 | 2.42 | 0.52 | low |
| 55-7573 | 0.19 | 0.69 | 1.41 | 0.14 | 0.84 | 0.00 | 1.59 | 1.76 | 0.56 | 0.73 | low |
| 86-7714 | 0.14 | 0.51 | 1.10 | 0.00 | 1.24 | 0.00 | 1.02 | 1.07 | 0.58 | 0.69 | low |
| 99-8025 | 0.12 | 0.11 | 1.76 | 0.10 | 0.11 | 0.20 | 2.24 | 1.74 | 1.34 | 2.21 | high |
| 55-8094 | 0.12 | 0.08 | 2.18 | 0.00 | 1.03 | 0.00 | 1.31 | 1.56 | 0.00 | 0.93 | low |
| 80-5608 | 0.19 | 0.32 | 0.58 | 0.02 | 0.21 | 0.00 | 1.97 | 1.22 | 0.31 | 1.13 | high |
| 35-4122 | 0.15 | 0.47 | 1.02 | 0.03 | 0.70 | 0.33 | 1.42 | 0.07 | 0.78 | 1.92 | high |
| 55-8513 | 0.00 | 2.16 | 0.96 | 0.09 | 2.08 | 0.00 | 1.57 | 2.19 | 0.16 | 0.10 | low |
| 55-6982 | 0.67 | 0.39 | 1.57 | 0.07 | 2.39 | 0.25 | 1.48 | 0.86 | 2.15 | 2.49 | high |
| 86-8279 | 0.30 | 0.31 | 1.58 | 0.74 | 1.62 | 0.12 | 2.18 | 1.89 | 1.81 | 1.61 | high |
| 55-7815 | 0.00 | 0.30 | 1.64 | 0.13 | 1.61 | 0.16 | 0.95 | 0.43 | 1.74 | 1.37 | high |
| 78-7162 | 0.45 | 0.81 | 0.80 | 0.45 | 1.02 | 0.00 | 1.81 | 0.52 | 0.00 | 1.24 | high |
| NJ-A55A | 0.00 | 0.70 | 1.10 | 0.06 | 1.01 | 0.00 | 1.80 | 1.90 | 0.83 | 0.51 | low |
| 44-6777 | 0.00 | 0.81 | 0.89 | 0.04 | 1.50 | 0.00 | 1.60 | 0.50 | 0.80 | 0.66 | low |
| 05-4422 | 0.41 | 0.41 | 0.30 | 0.21 | 1.60 | 0.12 | 2.24 | 1.80 | 0.15 | 0.75 | low |
| 05-4417 | 0.14 | 0.37 | 1.24 | 0.04 | 0.36 | 0.00 | 1.59 | 0.57 | 1.82 | 2.11 | high |
| 97-8175 | 0.13 | 0.39 | 0.15 | 0.05 | 2.33 | 0.06 | 1.58 | 0.88 | 0.57 | 0.52 | low |
| 91-8497 | 0.08 | 0.69 | 0.50 | 0.03 | 0.82 | 0.00 | 1.33 | 1.70 | 0.24 | 0.43 | low |
| 38-4632 | 0.18 | 0.86 | 0.97 | 0.03 | 1.69 | 0.52 | 1.77 | 1.32 | 0.64 | 0.79 | low |
| 67-3770 | 0.37 | 1.16 | 0.32 | 0.02 | 3.25 | 0.13 | 1.83 | 1.61 | 0.00 | 0.23 | low |
| 55-6972 | 0.86 | 0.30 | 0.54 | 0.75 | 3.45 | 0.00 | 3.43 | 1.50 | 0.00 | 1.22 | high |
| MP-A4TA | 0.58 | 0.22 | 2.14 | 3.72 | 2.03 | 0.63 | 4.29 | 1.74 | 1.38 | 11.88 | high |
| 55-7994 | 0.15 | 0.60 | 1.02 | 0.09 | 4.01 | 0.42 | 2.23 | 1.12 | 0.39 | 0.44 | low |
| 55-7724 | 0.10 | 0.71 | 1.31 | 0.07 | 1.28 | 0.25 | 1.23 | 1.40 | 0.46 | 0.66 | low |
| 97-7546 | 0.13 | 0.66 | 1.35 | 0.06 | 2.69 | 0.09 | 1.24 | 1.11 | 0.22 | 0.41 | low |
| 78-7154 | 0.27 | 0.31 | 0.66 | 0.62 | 1.11 | 0.18 | 2.92 | 1.41 | 0.00 | 1.34 | high |
| 50-5068 | 0.24 | 0.70 | 0.59 | 0.00 | 2.88 | 0.11 | 3.18 | 1.97 | 0.49 | 0.43 | low |
| 05-4250 | 0.10 | 0.38 | 0.92 | 0.03 | 0.83 | 0.63 | 1.68 | 0.48 | 1.37 | 2.29 | high |
| 62-A472 | 0.58 | 0.65 | 1.32 | 0.02 | 3.33 | 0.00 | 2.38 | 1.41 | 0.42 | 0.67 | low |
| 44-8120 | 0.09 | 0.59 | 1.05 | 0.00 | 1.13 | 0.00 | 3.03 | 1.03 | 1.19 | 1.25 | high |
| O1-A52J | 0.56 | 1.41 | 1.27 | 0.00 | 3.31 | 0.00 | 1.41 | 2.59 | 0.48 | 0.20 | low |
| 67-6217 | 0.07 | 0.94 | 1.67 | 0.09 | 2.92 | 0.00 | 1.04 | 1.12 | 0.23 | 0.30 | low |
| 38-A44F | 0.17 | 0.87 | 0.79 | 0.12 | 0.43 | 0.08 | 1.56 | 1.84 | 2.01 | 0.92 | low |
| 55-7281 | 0.12 | 0.77 | 1.41 | 0.00 | 2.03 | 0.16 | 1.33 | 0.41 | 1.63 | 1.02 | high |
| 64-1678 | 0.27 | 0.68 | 0.52 | 0.02 | 1.38 | 0.27 | 1.68 | 1.41 | 0.53 | 0.73 | low |
| 05-4395 | 0.40 | 0.21 | 1.51 | 0.70 | 2.21 | 0.08 | 4.51 | 0.79 | 0.30 | 2.90 | high |
| 97-7553 | 0.00 | 0.72 | 1.02 | 0.06 | 1.18 | 0.00 | 2.12 | 1.47 | 0.50 | 0.56 | low |
| 69-7763 | 0.07 | 0.48 | 1.02 | 0.61 | 1.64 | 0.09 | 1.46 | 0.62 | 1.14 | 1.07 | high |
| 55-8089 | 0.54 | 0.51 | 2.05 | 2.90 | 0.00 | 0.25 | 2.47 | 1.15 | 0.19 | 6.79 | high |
| 49-4501 | 0.55 | 0.62 | 0.63 | 0.04 | 1.76 | 0.04 | 1.94 | 0.52 | 0.48 | 1.29 | high |
| J2-A4AD | 0.32 | 0.50 | 1.57 | 0.07 | 3.39 | 0.19 | 4.00 | 2.03 | 1.22 | 0.92 | low |
| 50-5051 | 0.21 | 1.55 | 1.00 | 0.01 | 2.75 | 0.09 | 3.55 | 1.69 | 0.61 | 0.36 | low |
| 97-7552 | 0.00 | 0.55 | 1.57 | 0.12 | 0.27 | 0.13 | 1.14 | 0.98 | 0.32 | 1.01 | high |
| 44-2657 | 0.06 | 0.55 | 0.30 | 0.11 | 0.66 | 0.08 | 1.41 | 1.12 | 0.71 | 0.72 | low |
| 91-6840 | 0.48 | 0.39 | 0.78 | 0.00 | 0.29 | 0.79 | 1.81 | 0.92 | 0.74 | 3.40 | high |
| 49-4510 | 0.06 | 0.66 | 0.40 | 0.02 | 1.03 | 0.00 | 0.95 | 1.29 | 0.33 | 0.43 | low |
| 35-3615 | 0.23 | 0.71 | 1.21 | 0.13 | 4.80 | 0.00 | 2.44 | 1.34 | 0.17 | 0.25 | low |
| 55-A491 | 0.10 | 0.07 | 1.22 | 0.07 | 0.39 | 0.00 | 1.27 | 1.33 | 0.95 | 1.26 | high |
| L9-A444 | 0.12 | 0.36 | 0.60 | 0.04 | 2.81 | 0.00 | 1.29 | 1.16 | 1.31 | 0.48 | low |
| 55-6970 | 0.36 | 0.50 | 0.84 | 0.42 | 1.09 | 0.06 | 2.55 | 0.61 | 0.27 | 1.70 | high |
| 49-6745 | 0.00 | 0.54 | 1.72 | 0.04 | 0.56 | 0.38 | 1.95 | 0.51 | 0.97 | 1.99 | high |
| MP-A4TH | 0.06 | 0.59 | 0.49 | 0.31 | 2.74 | 0.00 | 1.63 | 1.65 | 0.18 | 0.26 | low |
| 55-8508 | 0.07 | 0.30 | 0.51 | 0.24 | 1.90 | 0.00 | 1.89 | 0.90 | 0.84 | 0.73 | low |
| 49-6742 | 0.51 | 0.35 | 1.67 | 2.44 | 1.11 | 0.21 | 3.35 | 1.00 | 0.00 | 4.86 | high |
| 55-7913 | 0.11 | 0.08 | 1.72 | 0.01 | 0.45 | 0.31 | 2.45 | 1.22 | 0.34 | 2.09 | high |
| 95-7944 | 0.12 | 0.15 | 1.48 | 2.92 | 0.76 | 0.24 | 2.02 | 0.98 | 1.61 | 4.63 | high |
| 55-6981 | 0.00 | 0.41 | 0.79 | 0.02 | 1.42 | 0.00 | 2.01 | 1.09 | 1.18 | 0.80 | low |
| 62-8402 | 0.07 | 0.15 | 0.80 | 0.01 | 0.78 | 0.18 | 1.04 | 2.54 | 0.23 | 0.47 | low |
| 44-3918 | 0.65 | 0.78 | 2.34 | 0.46 | 5.10 | 0.21 | 1.73 | 1.16 | 0.54 | 0.62 | low |
| 38-6178 | 0.13 | 0.35 | 1.29 | 0.02 | 1.37 | 0.00 | 2.31 | 0.61 | 0.71 | 1.32 | high |
| 78-7535 | 0.25 | 0.07 | 1.42 | 0.00 | 1.81 | 0.08 | 2.99 | 1.29 | 0.48 | 1.41 | high |
| 55-A493 | 0.14 | 0.71 | 1.47 | 0.03 | 1.16 | 0.60 | 1.90 | 1.25 | 1.62 | 1.62 | high |
| 55-A48X | 0.36 | 0.58 | 1.38 | 0.07 | 0.34 | 0.00 | 1.83 | 2.31 | 0.78 | 0.99 | high |
| 95-7567 | 0.36 | 0.10 | 1.09 | 0.06 | 2.23 | 0.05 | 1.55 | 0.65 | 0.33 | 1.11 | high |
| 55-7910 | 0.00 | 0.22 | 1.69 | 0.12 | 1.05 | 0.00 | 2.68 | 1.82 | 0.72 | 1.02 | high |
| 55-8302 | 0.27 | 0.29 | 1.74 | 0.02 | 1.19 | 0.18 | 1.86 | 1.23 | 0.97 | 1.64 | high |
| 95-8494 | 0.00 | 0.20 | 0.65 | 0.03 | 1.63 | 0.53 | 2.07 | 1.83 | 0.85 | 0.80 | low |
| 49-4505 | 0.08 | 0.57 | 1.21 | 0.00 | 1.04 | 0.07 | 1.88 | 0.25 | 0.71 | 1.32 | high |
| 55-1592 | 0.11 | 0.91 | 1.96 | 0.02 | 0.93 | 0.05 | 2.61 | 2.43 | 0.23 | 0.57 | low |
| 05-4397 | 0.20 | 3.12 | 0.08 | 0.01 | 1.88 | 0.97 | 2.97 | 2.27 | 0.24 | 0.17 | low |
| 55-A48Z | 0.39 | 0.69 | 1.27 | 0.15 | 1.05 | 0.00 | 1.53 | 1.37 | 1.71 | 1.33 | high |
| 97-8547 | 0.27 | 1.11 | 1.07 | 0.06 | 1.96 | 0.00 | 2.41 | 1.55 | 2.64 | 0.90 | low |
| 75-6212 | 0.00 | 1.38 | 0.48 | 0.12 | 2.33 | 0.11 | 0.87 | 1.34 | 0.15 | 0.17 | low |
| 86-8281 | 0.32 | 0.73 | 1.15 | 0.47 | 4.33 | 0.00 | 2.04 | 1.17 | 0.62 | 0.38 | low |
| 62-8397 | 0.00 | 0.53 | 0.67 | 1.21 | 0.90 | 0.14 | 0.39 | 1.11 | 0.34 | 0.67 | low |
| 78-7153 | 0.25 | 0.04 | 0.48 | 0.16 | 0.51 | 0.26 | 1.53 | 0.58 | 0.54 | 1.98 | high |
| MP-A4T8 | 0.16 | 0.12 | 1.27 | 0.01 | 3.04 | 0.00 | 2.91 | 1.09 | 1.46 | 1.03 | high |
| 44-6778 | 0.08 | 0.65 | 0.99 | 0.08 | 0.40 | 0.20 | 1.47 | 1.60 | 0.25 | 0.73 | low |
| 44-7670 | 0.00 | 0.20 | 1.20 | 0.07 | 0.37 | 0.00 | 2.05 | 2.89 | 1.65 | 0.77 | low |
| 55-8512 | 0.07 | 0.37 | 0.87 | 0.21 | 1.45 | 0.00 | 1.76 | 1.44 | 0.00 | 0.55 | low |
| 44-A47B | 0.25 | 0.79 | 1.33 | 0.06 | 2.06 | 0.39 | 0.89 | 2.15 | 0.97 | 0.48 | low |
| 50-5946 | 0.18 | 0.21 | 1.36 | 0.01 | 0.98 | 0.12 | 2.00 | 1.77 | 0.96 | 1.16 | high |
| 86-7954 | 0.05 | 0.34 | 0.74 | 0.05 | 2.30 | 0.17 | 1.73 | 1.32 | 0.72 | 0.55 | low |
| 73-7499 | 0.74 | 0.43 | 0.93 | 0.02 | 1.87 | 0.34 | 2.05 | 2.46 | 0.48 | 1.05 | high |
| 49-AAR0 | 0.21 | 0.63 | 0.76 | 0.16 | 1.99 | 0.14 | 2.56 | 2.84 | 0.66 | 0.40 | low |
| 67-3774 | 0.15 | 0.85 | 0.71 | 0.53 | 1.24 | 0.00 | 2.09 | 0.86 | 0.00 | 0.72 | low |
| 69-7760 | 0.21 | 0.15 | 0.32 | 0.02 | 2.62 | 0.21 | 1.90 | 0.67 | 0.49 | 0.79 | low |
| 50-5935 | 0.39 | 0.32 | 0.93 | 0.01 | 1.63 | 0.00 | 1.63 | 1.19 | 0.88 | 1.07 | high |
| 95-7043 | 0.00 | 0.09 | 0.70 | 0.04 | 2.06 | 0.00 | 2.71 | 2.05 | 0.93 | 0.56 | low |
| MP-A4T7 | 0.25 | 0.34 | 1.10 | 0.44 | 2.08 | 0.00 | 3.00 | 1.85 | 0.17 | 0.77 | low |
| 44-2659 | 0.00 | 0.56 | 1.09 | 0.05 | 1.85 | 0.12 | 1.40 | 0.47 | 1.37 | 0.89 | low |
| 86-8076 | 0.12 | 0.15 | 1.21 | 0.06 | 1.75 | 0.05 | 1.98 | 1.32 | 0.36 | 0.81 | low |
| L4-A4E5 | 0.53 | 0.31 | 1.88 | 0.05 | 1.61 | 0.19 | 2.13 | 1.02 | 0.46 | 2.09 | high |
| 97-8171 | 0.62 | 0.36 | 0.50 | 0.00 | 1.11 | 0.13 | 1.64 | 2.27 | 0.21 | 0.84 | low |
| S2-AA1A | 0.06 | 0.75 | 0.70 | 0.13 | 0.99 | 0.00 | 1.72 | 1.69 | 0.68 | 0.51 | low |
| 64-5781 | 0.13 | 0.21 | 1.22 | 1.00 | 2.36 | 0.53 | 4.56 | 0.78 | 1.43 | 3.69 | high |
| 78-7159 | 0.45 | 0.18 | 1.47 | 0.09 | 0.75 | 0.15 | 1.94 | 1.10 | 0.75 | 2.40 | high |
| 69-8254 | 0.06 | 0.58 | 0.45 | 2.72 | 1.02 | 0.04 | 1.63 | 1.16 | 0.05 | 1.05 | high |
| 49-4512 | 0.07 | 0.74 | 0.55 | 0.13 | 1.14 | 0.00 | 1.34 | 1.53 | 2.47 | 0.75 | low |
| 55-1596 | 0.50 | 0.33 | 0.92 | 0.00 | 3.60 | 0.39 | 5.18 | 1.28 | 0.48 | 1.70 | high |
| 49-6743 | 0.15 | 0.16 | 1.91 | 0.07 | 5.03 | 0.20 | 3.41 | 1.23 | 0.64 | 0.59 | low |
| 67-6215 | 0.08 | 0.62 | 1.26 | 0.03 | 2.36 | 0.11 | 1.74 | 1.06 | 0.00 | 0.48 | low |
| 75-6214 | 0.26 | 0.08 | 2.10 | 0.99 | 0.57 | 0.22 | 1.46 | 0.55 | 0.74 | 3.72 | high |
| 97-7554 | 0.20 | 0.32 | 1.47 | 0.05 | 0.85 | 0.07 | 1.16 | 0.35 | 0.89 | 1.71 | high |
| 50-6594 | 1.02 | 0.42 | 0.86 | 0.00 | 1.28 | 0.25 | 2.07 | 0.50 | 1.63 | 5.33 | high |
| 62-A46V | 0.53 | 1.12 | 0.92 | 0.02 | 3.82 | 0.33 | 2.11 | 2.49 | 2.06 | 0.42 | low |
| 69-8453 | 0.12 | 0.83 | 0.84 | 0.23 | 0.16 | 0.29 | 1.31 | 1.37 | 0.19 | 0.82 | low |

Supplementary Table 5 Identification of DEGs between low- and high-risk groups

| gene | lowMean | highMean | logFC | pValue | fdr |
| --- | --- | --- | --- | --- | --- |
| TMEM14EP | 2.375339382 | 0.477787702 | -2.313692033 | 0.020415855 | 0.037514598 |
| FSCN1 | 73.2070166 | 149.7844672 | 1.032834186 | 4.12E-10 | 1.24E-08 |
| PLA2G4F | 11.86370695 | 4.872020161 | -1.283962858 | 5.16E-13 | 5.09E-11 |
| BTBD16 | 1.01041583 | 2.026674395 | 1.004165177 | 0.000372036 | 0.001232635 |
| SFTPA2 | 5743.152198 | 2800.980929 | -1.035910634 | 4.57E-08 | 6.21E-07 |
| AC110741.1 | 5.902556242 | 0.950088844 | -2.63520555 | 0.008883291 | 0.018570561 |
| SNRPGP4 | 2.388887516 | 1.108154099 | -1.108180408 | 0.000533005 | 0.001677629 |
| DNER | 4.322712484 | 9.462683737 | 1.130312527 | 0.000209623 | 0.000753058 |
| RNY4P25 | 1.766219048 | 0.666798656 | -1.405341177 | 7.90E-05 | 0.00032842 |
| LAMC2 | 82.29273372 | 241.8493714 | 1.555271834 | 2.82E-15 | 9.33E-13 |
| MIR126 | 5.053699356 | 2.463880645 | -1.03640747 | 6.88E-07 | 6.05E-06 |
| CFAP73 | 4.606684299 | 2.097539651 | -1.135030649 | 0.001503119 | 0.004085771 |
| KCNH6 | 1.490868082 | 0.608998253 | -1.291642614 | 0.001074016 | 0.003065888 |
| SLC16A11 | 5.314571042 | 2.590341062 | -1.036811186 | 1.12E-09 | 2.88E-08 |
| HOXA10 | 1.293159202 | 3.049767675 | 1.237799448 | 3.24E-06 | 2.22E-05 |
| ARNTL2 | 10.06503874 | 23.952142 | 1.250801955 | 2.73E-14 | 5.46E-12 |
| SERPINB5 | 6.454900515 | 24.01907379 | 1.895713754 | 1.95E-08 | 3.01E-07 |
| F11 | 1.62293269 | 0.638026546 | -1.346914811 | 3.94E-09 | 7.86E-08 |
| FURIN | 191.9408176 | 387.6280047 | 1.01401126 | 3.28E-07 | 3.25E-06 |
| RNU1-38P | 1.769698198 | 0.744820363 | -1.248538925 | 4.53E-09 | 8.82E-08 |
| MIR6774 | 1.747499099 | 0.872830914 | -1.001517607 | 8.20E-07 | 6.97E-06 |
| CYP4Z2P | 2.847562677 | 0.834018481 | -1.771576339 | 5.73E-15 | 1.57E-12 |
| SLC2A1 | 60.07216332 | 131.8925342 | 1.134594379 | 2.27E-14 | 4.76E-12 |
| KRT81 | 15.3595305 | 72.71824812 | 2.243183326 | 1.10E-07 | 1.30E-06 |
| SLC25A47P1 | 1.704848649 | 0.641781922 | -1.40948861 | 0.002351681 | 0.005983342 |
| RAP1AP | 2.206648649 | 1.047168145 | -1.07536382 | 2.10E-11 | 1.06E-09 |
| ANLN | 17.66986718 | 38.04331835 | 1.106351896 | 6.31E-19 | 2.71E-15 |
| FLNC | 5.459142214 | 16.83262849 | 1.624514292 | 2.98E-10 | 9.47E-09 |
| LAMA3 | 13.98489202 | 34.36712272 | 1.297159957 | 0.000206386 | 0.000742826 |
| PI3 | 25.11979807 | 156.6255476 | 2.640422781 | 3.35E-05 | 0.000159601 |
| CDHR2 | 1.410458687 | 4.651594355 | 1.721560882 | 3.07E-06 | 2.13E-05 |
| FAM83A | 45.31258867 | 111.3987472 | 1.297749189 | 2.03E-15 | 7.57E-13 |
| AC018629.1 | 1.832461133 | 4.883472245 | 1.414124699 | 4.15E-06 | 2.75E-05 |
| KRT14 | 4.925285199 | 29.05086949 | 2.560302172 | 0.005297549 | 0.01192747 |
| CHGB | 20.79102278 | 55.65803488 | 1.420629241 | 0.013975938 | 0.027215216 |
| MYEOV | 12.87546551 | 26.21516599 | 1.025777088 | 3.19E-06 | 2.19E-05 |
| AP000357.2 | 7.180281338 | 2.016732796 | -1.832020424 | 1.89E-09 | 4.36E-08 |
| LRRC66 | 0.801018018 | 1.836151142 | 1.196778219 | 0.00326605 | 0.007912491 |
| CYP2F1 | 1.987759331 | 0.812637231 | -1.290459724 | 0.010487798 | 0.021372466 |
| COL22A1 | 1.723417375 | 3.791528293 | 1.137507355 | 4.53E-06 | 2.97E-05 |
| DNAH9 | 1.940203089 | 0.800395094 | -1.277423443 | 0.002155297 | 0.005556248 |
| SNORA47 | 3.585769884 | 1.513047849 | -1.244825294 | 8.51E-05 | 0.000349373 |
| FCER2 | 2.152044273 | 1.070289785 | -1.007706293 | 4.37E-05 | 0.000199384 |
| TUBAL3 | 0.785266795 | 2.119064583 | 1.432172758 | 2.75E-05 | 0.000135106 |
| SFTPA1 | 4723.736019 | 2238.437691 | -1.077436184 | 5.73E-09 | 1.06E-07 |
| MFAP5 | 4.684573874 | 11.28956478 | 1.269000145 | 8.38E-05 | 0.00034489 |
| MMP10 | 8.215379022 | 26.34946196 | 1.681374462 | 0.006597256 | 0.014371578 |
| LINC01765 | 2.695114672 | 1.320942137 | -1.028779386 | 0.010848858 | 0.021980485 |
| LYPD3 | 14.02962793 | 40.56471653 | 1.531748658 | 4.33E-09 | 8.50E-08 |
| OPN3 | 4.579289447 | 9.456079167 | 1.046118356 | 3.36E-13 | 3.69E-11 |
| WNT7A | 1.306491248 | 4.968297648 | 1.927054146 | 0.010923527 | 0.022108303 |
| SUSD2 | 177.4970609 | 76.72034751 | -1.210113975 | 3.11E-10 | 9.78E-09 |
| AC090001.1 | 1.585236293 | 0.655153495 | -1.274793045 | 0.000138022 | 0.000526906 |
| SFTPD | 199.7253945 | 88.26404335 | -1.178120035 | 1.49E-10 | 5.40E-09 |
| MIR3189 | 26.60822728 | 11.70110753 | -1.185227308 | 5.82E-12 | 3.61E-10 |
| LINC01559 | 1.029690347 | 3.678264046 | 1.836814498 | 4.12E-05 | 0.000189436 |
| LRRK2 | 36.48103681 | 15.84414194 | -1.203197202 | 2.08E-12 | 1.63E-10 |
| AL022098.1 | 1.540288932 | 0.60960578 | -1.337252515 | 9.18E-12 | 5.22E-10 |
| BANCR | 6.748788417 | 1.832690188 | -1.880665602 | 1.94E-05 | 0.000100281 |
| PTHLH | 2.901642728 | 7.306453226 | 1.332301353 | 2.50E-10 | 8.21E-09 |
| LINC01116 | 2.309257529 | 4.94021418 | 1.097144519 | 6.80E-09 | 1.23E-07 |
| RNA5SP217 | 2.713941055 | 1.334552285 | -1.024033559 | 6.77E-05 | 0.000288184 |
| GDF10 | 5.62968314 | 2.77170746 | -1.022278728 | 8.08E-06 | 4.83E-05 |
| ADH1B | 20.2766287 | 8.951546841 | -1.179608893 | 2.13E-11 | 1.07E-09 |
| DNASE1L3 | 3.833863192 | 1.807081116 | -1.08513759 | 9.02E-10 | 2.38E-08 |
| PKP2 | 6.577350579 | 13.39564462 | 1.026185534 | 3.35E-09 | 6.92E-08 |
| DCDC2B | 1.931981338 | 0.89466129 | -1.110667658 | 2.43E-05 | 0.000121468 |
| PRDM16-DT | 2.735511326 | 1.355260148 | -1.013240719 | 2.40E-09 | 5.23E-08 |
| TMEM213 | 6.234859846 | 2.339076478 | -1.414418097 | 5.43E-05 | 0.000238879 |
| CLC | 3.788007207 | 0.872987433 | -2.117406285 | 0.005115871 | 0.011582315 |
| AC108215.1 | 4.495772201 | 1.618002823 | -1.474354812 | 9.24E-12 | 5.24E-10 |
| GATA6-AS1 | 1.507322523 | 0.69314879 | -1.120751167 | 5.02E-09 | 9.54E-08 |
| MGAT5B | 0.560369498 | 1.867211425 | 1.736434959 | 2.00E-06 | 1.48E-05 |
| LRRK2-DT | 15.46298893 | 7.637777151 | -1.017594481 | 1.75E-15 | 6.68E-13 |
| PTGES | 54.10393925 | 108.4718997 | 1.003515808 | 8.41E-08 | 1.04E-06 |
| AC236972.3 | 2.586401544 | 1.142206384 | -1.17912292 | 4.91E-10 | 1.44E-08 |
| PRR20G | 1.08179305 | 2.469627957 | 1.190869186 | 0.000442612 | 0.001430329 |
| HTR1D | 2.757804376 | 5.596709409 | 1.021058719 | 1.17E-08 | 1.94E-07 |
| LHFPL3 | 2.531975676 | 1.079362097 | -1.230084614 | 9.18E-13 | 8.39E-11 |
| DNAI2 | 3.276561647 | 1.279557191 | -1.356538045 | 1.03E-05 | 5.90E-05 |
| AC073648.6 | 7.079214672 | 3.090098723 | -1.195936394 | 0.000533941 | 0.001679958 |
| AC083809.1 | 2.523871686 | 5.081079503 | 1.009496473 | 0.005493087 | 0.012301622 |
| AL928596.1 | 5.980767954 | 2.646874798 | -1.176040789 | 6.19E-06 | 3.87E-05 |
| AC105118.1 | 4.402829086 | 1.912796371 | -1.202747543 | 0.018226091 | 0.034051769 |
| HOXA1 | 1.634027156 | 3.895679032 | 1.253442858 | 2.89E-08 | 4.19E-07 |
| CRABP1 | 4.509753153 | 14.72824079 | 1.707464746 | 0.006847092 | 0.014821848 |
| CACNA2D2 | 36.62537735 | 17.39736593 | -1.073974734 | 3.33E-12 | 2.35E-10 |
| AC003092.1 | 0.729957143 | 2.950971237 | 2.01530619 | 0.001353567 | 0.00373361 |
| CFLAR-AS1 | 1.983958945 | 0.887597984 | -1.160403876 | 0.000452665 | 0.001455974 |
| AC113349.1 | 1.616260618 | 0.564576949 | -1.517417716 | 0.002544517 | 0.006386013 |
| PDE10A | 1.143092793 | 3.046910551 | 1.414404625 | 4.65E-07 | 4.37E-06 |
| S100P | 1108.516707 | 2283.82395 | 1.04282093 | 0.000131053 | 0.000504902 |
| AL353804.2 | 1.754014929 | 0.717867137 | -1.288872268 | 0.000808888 | 0.002395016 |
| DNAAF6 | 1.533638481 | 0.761974933 | -1.009143 | 0.012316805 | 0.024441537 |
| SNORD89 | 10.5276816 | 4.740987836 | -1.150928165 | 0.004749098 | 0.010875067 |
| CYP24A1 | 51.2848417 | 123.9298935 | 1.272919851 | 6.62E-06 | 4.08E-05 |
| FGB | 73.76934762 | 313.7479873 | 2.088512822 | 0.007836193 | 0.016656516 |
| NR4A1AS | 5.06749408 | 2.44970672 | -1.048663459 | 9.97E-06 | 5.76E-05 |
| PCSK1 | 15.9858112 | 51.56934805 | 1.689721852 | 2.12E-05 | 0.000108453 |
| AC245041.1 | 1.314583912 | 4.868157796 | 1.888769697 | 0.01530527 | 0.02939399 |
| RANBP20P | 1.785024839 | 0.220531183 | -3.01688958 | 0.000846097 | 0.002489739 |
| PADI3 | 3.173719048 | 7.339056586 | 1.209420201 | 0.002240359 | 0.005737497 |
| SNORD9 | 3.339848906 | 1.386584745 | -1.268247044 | 0.000112076 | 0.000442094 |
| EXO1 | 5.430344788 | 11.24923481 | 1.050711164 | 3.51E-18 | 1.01E-14 |
| AL357093.2 | 2.090548391 | 0.903510148 | -1.210268729 | 1.44E-05 | 7.78E-05 |
| KCNF1 | 0.908292278 | 3.297590995 | 1.86018395 | 5.63E-11 | 2.42E-09 |
| AC112721.2 | 0.693251223 | 1.767992339 | 1.350661862 | 5.90E-07 | 5.33E-06 |
| KRT83 | 0.742225097 | 1.961173992 | 1.401788846 | 0.000175768 | 0.000647965 |
| CFAP65 | 1.620965122 | 0.718975269 | -1.172838998 | 0.001092106 | 0.003109777 |
| VEGFD | 10.997526 | 3.918923185 | -1.488649812 | 4.63E-13 | 4.65E-11 |
| ECT2L | 2.000280051 | 0.959172581 | -1.060339677 | 0.021044993 | 0.038497786 |
| LGALS7B | 2.918348777 | 8.37602789 | 1.521113933 | 0.005296164 | 0.01192747 |
| ERICH3 | 2.53925251 | 1.164116263 | -1.125168716 | 0.005756296 | 0.01278937 |
| RPL13AP17 | 2.516919048 | 0.711829704 | -1.822054773 | 8.84E-13 | 8.17E-11 |
| CASP14 | 0.387048263 | 3.974682661 | 3.360254302 | 4.99E-05 | 0.000222751 |
| SLC10A2 | 3.290037066 | 1.268358333 | -1.375141448 | 7.63E-05 | 0.000319304 |
| AC068587.2 | 4.879133462 | 2.041929032 | -1.256692221 | 7.08E-09 | 1.27E-07 |
| TCN1 | 36.58795238 | 95.31858481 | 1.381388854 | 0.00055361 | 0.001732929 |
| AL138760.1 | 1.598816216 | 3.33947957 | 1.062619178 | 0.003534908 | 0.008451801 |
| SCGB2A1 | 30.10407259 | 10.73134227 | -1.488128134 | 0.002942174 | 0.007236022 |
| PGC | 3392.262822 | 1647.882858 | -1.041634259 | 4.74E-09 | 9.15E-08 |
| AL445493.3 | 2.683005019 | 1.242469825 | -1.110638939 | 2.54E-09 | 5.50E-08 |
| RNU6-853P | 2.076970656 | 0.956373656 | -1.118834538 | 0.016028076 | 0.030553297 |
| AL133304.2 | 2.761460103 | 0.929476277 | -1.570941335 | 1.14E-05 | 6.43E-05 |
| HMGA2 | 1.533527027 | 7.315486358 | 2.25410019 | 1.20E-05 | 6.70E-05 |
| C1orf87 | 1.815685457 | 0.831057796 | -1.127493579 | 0.002434049 | 0.006155531 |
| TENM3 | 1.412997941 | 2.907223723 | 1.040882734 | 6.60E-06 | 4.07E-05 |
| GSTA2 | 27.05060283 | 9.349197715 | -1.532746272 | 4.14E-05 | 0.000190557 |
| IL20RB | 8.230527027 | 16.79354039 | 1.02884969 | 0.000120657 | 0.000469483 |
| LRRC38 | 0.145510811 | 7.695501747 | 5.724817145 | 0.002346129 | 0.00597187 |
| AC120498.2 | 4.3945426 | 1.664477016 | -1.400644063 | 3.76E-11 | 1.72E-09 |
| DNAH12 | 1.437934234 | 0.551863911 | -1.381613244 | 1.16E-05 | 6.53E-05 |
| AC106045.1 | 2.770277735 | 5.899256653 | 1.090502555 | 9.38E-06 | 5.47E-05 |
| SFRP5 | 3.494262162 | 1.539719422 | -1.182320375 | 9.50E-05 | 0.000383517 |
| AC094019.2 | 1.674488288 | 0.830454704 | -1.011746899 | 8.65E-11 | 3.47E-09 |
| SOX15 | 3.646133848 | 8.242335148 | 1.176685604 | 0.000849036 | 0.002496253 |
| TRIM31 | 5.196474775 | 13.57940269 | 1.385814868 | 0.003586674 | 0.008557704 |
| INMT | 33.26500553 | 16.22244718 | -1.036013805 | 5.76E-10 | 1.64E-08 |
| SERPINB7 | 1.189529987 | 3.128417876 | 1.395041591 | 2.35E-05 | 0.000118243 |
| ALKAL2 | 1.863467696 | 0.879264516 | -1.083620657 | 2.82E-07 | 2.87E-06 |
| FGF19 | 3.179472072 | 6.948530847 | 1.127920739 | 0.000235868 | 0.000833828 |
| MALAT1 | 441.5828645 | 145.8219145 | -1.598476642 | 1.43E-06 | 1.12E-05 |
| MPHOSPH6P1 | 1.571782754 | 0.603297446 | -1.381460447 | 0.000300031 | 0.001026006 |
| AFF2 | 1.892386486 | 0.871853898 | -1.118048465 | 0.003137476 | 0.007647375 |
| RHCG | 1.266586486 | 6.27512789 | 2.30869927 | 0.001194568 | 0.003364738 |
| SH3PXD2A-AS1 | 0.933734749 | 2.241129234 | 1.263141164 | 1.91E-08 | 2.96E-07 |
| LHFPL3-AS2 | 12.09961815 | 4.55756875 | -1.408625194 | 2.89E-12 | 2.13E-10 |
| AC112722.1 | 1.685935521 | 0.725428696 | -1.21664364 | 7.24E-15 | 1.87E-12 |
| IL11 | 1.473822136 | 3.19115457 | 1.114516062 | 8.83E-09 | 1.53E-07 |
| AC007684.1 | 2.556702188 | 1.064734879 | -1.26378988 | 0.001302287 | 0.003617137 |
| AL162511.1 | 6.482729601 | 2.544203024 | -1.349387598 | 6.65E-16 | 3.57E-13 |
| LINC02009 | 5.958068597 | 2.757124261 | -1.111680444 | 0.002233146 | 0.005725853 |
| ERVMER34-1 | 2.857013642 | 6.635242473 | 1.215641262 | 3.00E-06 | 2.08E-05 |
| IL22RA1 | 3.33391094 | 7.20979953 | 1.11274358 | 1.96E-10 | 6.72E-09 |
| LOXL2 | 19.86661725 | 43.01850739 | 1.114611228 | 2.33E-12 | 1.79E-10 |
| RNVU1-15 | 2.163178636 | 0.995636156 | -1.119462281 | 4.79E-05 | 0.000214965 |
| MECOM-AS1 | 1.90149305 | 0.799716667 | -1.249571806 | 3.49E-05 | 0.000164956 |
| SRGAP3-AS2 | 7.402158172 | 3.328372849 | -1.153128909 | 0.00135947 | 0.003748087 |
| ARL14 | 4.637236293 | 13.83034516 | 1.576500015 | 7.77E-05 | 0.000324146 |
| TCTE1 | 1.483971686 | 0.667602554 | -1.152402187 | 0.002538193 | 0.006374797 |
| IGFBP1 | 3.925783012 | 12.00808387 | 1.61295362 | 1.97E-06 | 1.47E-05 |
| PAX8 | 1.688416602 | 4.095825067 | 1.278483179 | 0.011306768 | 0.022765947 |
| DMBT1 | 111.1982925 | 36.56755363 | -1.604498617 | 4.11E-06 | 2.73E-05 |
| PLA2G3 | 4.164392664 | 1.727157124 | -1.269706774 | 3.99E-08 | 5.50E-07 |
| COL7A1 | 5.49092574 | 12.23614341 | 1.156027616 | 8.95E-06 | 5.25E-05 |
| C22orf15 | 2.30064157 | 0.954133737 | -1.269772834 | 0.001756434 | 0.00466653 |
| SLC46A2 | 9.41622471 | 4.120044086 | -1.192488975 | 1.64E-09 | 3.87E-08 |
| CCDC17 | 8.845741699 | 3.775182997 | -1.228436536 | 3.08E-05 | 0.000148344 |
| C11orf97 | 2.799568597 | 1.275024395 | -1.13467968 | 0.01787467 | 0.033468006 |
| INHA | 13.07135187 | 35.97310544 | 1.460510353 | 0.000543767 | 0.001707546 |
| CLDN8 | 11.1807444 | 5.069641734 | -1.141060542 | 0.00053093 | 0.001671402 |
| RSPO3 | 3.211595495 | 8.962822782 | 1.480662976 | 0.000596232 | 0.001841878 |
| RNU5F-1 | 4.470748649 | 1.911782527 | -1.225598018 | 7.49E-05 | 0.000314425 |
| AP003555.1 | 0.69778121 | 1.839800672 | 1.398702817 | 1.63E-06 | 1.25E-05 |
| TREML2 | 1.67126139 | 0.707236895 | -1.240671948 | 0.001613509 | 0.004338472 |
| EFNA2 | 1.24376834 | 2.81177379 | 1.176762734 | 5.10E-09 | 9.66E-08 |
| AC026355.2 | 5.092762548 | 1.649104704 | -1.626765452 | 9.19E-22 | 1.58E-17 |
| TSPAN19 | 2.008977864 | 0.919669624 | -1.127274073 | 0.021356756 | 0.039014005 |
| MSTN | 8.077607336 | 0.207137097 | -5.285270158 | 1.18E-05 | 6.63E-05 |
| RNF186 | 1.053267568 | 2.207273522 | 1.067393438 | 0.000214497 | 0.000767036 |
| FCER1A | 24.38835431 | 11.56208098 | -1.076791328 | 3.56E-09 | 7.23E-08 |
| C1QL2 | 8.361865122 | 1.660223253 | -2.332447517 | 3.81E-07 | 3.70E-06 |
| BCL2L1-AS1 | 2.599946075 | 1.093813038 | -1.249115537 | 0.000859597 | 0.002520828 |
| MIR31HG | 0.752578636 | 2.063547312 | 1.455212276 | 3.15E-06 | 2.17E-05 |
| S100A7 | 5.527897169 | 44.17228925 | 2.998338919 | 0.008883854 | 0.018570561 |
| ACTL8 | 0.541205277 | 2.626282392 | 2.278774241 | 4.80E-07 | 4.48E-06 |
| DEFB1 | 39.66378945 | 84.53023569 | 1.091644954 | 0.007286261 | 0.015630705 |
| SNORA53 | 23.14032986 | 9.192378427 | -1.331899333 | 0.008008189 | 0.01697171 |
| TNFSF11 | 2.086243758 | 4.689358871 | 1.168482958 | 1.55E-05 | 8.32E-05 |
| PLA2G1B | 32.09872355 | 15.43461075 | -1.056346828 | 3.21E-07 | 3.19E-06 |
| AC090181.3 | 2.796020849 | 1.095688239 | -1.351537758 | 0.000649682 | 0.001982397 |
| CELF3 | 2.902312741 | 0.723158266 | -2.00481966 | 0.001099122 | 0.003126646 |
| IGF2 | 0.581099356 | 8.579528427 | 3.88404159 | 1.23E-05 | 6.83E-05 |
| TESMIN | 2.211258816 | 4.453123454 | 1.009949714 | 3.89E-12 | 2.65E-10 |
| MIR29B2CHG | 2.876030373 | 1.233624261 | -1.221175869 | 4.60E-13 | 4.65E-11 |
| SNORA22 | 10.03030798 | 4.364138777 | -1.20059702 | 0.014519508 | 0.028127044 |
| SNORA79B | 22.61566139 | 9.423001344 | -1.263063633 | 0.023156468 | 0.041838973 |
| FOSL1 | 12.87226152 | 36.49255168 | 1.503336491 | 2.75E-09 | 5.89E-08 |
| LINC00540 | 0.681513127 | 1.466596304 | 1.105658457 | 0.017829323 | 0.033390379 |
| HAPLN1 | 0.957517632 | 2.14319711 | 1.162393583 | 0.001489043 | 0.004055345 |
| KLK6 | 4.237916988 | 12.53532944 | 1.564572678 | 2.16E-07 | 2.29E-06 |
| HOXB13 | 2.113941828 | 4.826737366 | 1.191112652 | 0.000471417 | 0.001508378 |
| ARHGEF2-AS1 | 3.827740927 | 1.221927957 | -1.647333958 | 1.80E-08 | 2.81E-07 |
| SFTA1P | 29.62429073 | 13.85663031 | -1.096204151 | 1.76E-14 | 3.79E-12 |
| DYNLRB2 | 4.154861519 | 2.040431317 | -1.025926244 | 0.000794227 | 0.002357703 |
| CDA | 41.75925779 | 99.8704039 | 1.257961136 | 2.38E-06 | 1.72E-05 |
| AL355796.1 | 8.83229112 | 4.319414987 | -1.031951796 | 0.000246679 | 0.000867135 |
| AC239859.5 | 0.540262162 | 2.130992272 | 1.979793811 | 3.56E-08 | 5.00E-07 |
| MIR3677 | 7.426936937 | 3.703280242 | -1.0039636 | 1.11E-08 | 1.85E-07 |
| GJB3 | 8.533114801 | 28.53659913 | 1.741669048 | 2.30E-11 | 1.12E-09 |
| IL1R2 | 3.63832677 | 9.346337366 | 1.361125992 | 1.17E-07 | 1.38E-06 |
| SLC25A21 | 1.066237194 | 2.258116331 | 1.082591396 | 0.000911251 | 0.002651037 |
| IGF2BP1 | 1.003471429 | 6.590101815 | 2.715301214 | 1.67E-10 | 5.94E-09 |
| AC243967.2 | 3.833025354 | 1.885806384 | -1.023301979 | 9.85E-08 | 1.19E-06 |
| CPS1 | 43.98797928 | 107.5599794 | 1.289960151 | 2.60E-05 | 0.000128941 |
| POPDC3 | 1.618256885 | 4.850666734 | 1.58374242 | 3.10E-05 | 0.000149324 |
| LRRC18 | 1.654369627 | 0.797379637 | -1.052942936 | 0.002917048 | 0.007187604 |
| L1CAM | 1.430588546 | 4.378985148 | 1.613987761 | 0.009663267 | 0.019959753 |
| AL161431.1 | 1.222031789 | 4.545912634 | 1.895288143 | 0.00904933 | 0.018870895 |
| MS4A2 | 4.699709395 | 2.292768145 | -1.03548108 | 6.86E-10 | 1.89E-08 |
| AKAP14 | 2.766811068 | 1.319911828 | -1.067782574 | 0.002930158 | 0.007214285 |
| FAM83B | 1.036479537 | 2.461256855 | 1.247703591 | 0.000235554 | 0.000832974 |
| MIR4483 | 4.885525097 | 2.203258266 | -1.148875014 | 1.35E-05 | 7.38E-05 |
| CTSG | 4.62045843 | 2.047170228 | -1.174404927 | 3.11E-07 | 3.11E-06 |
| ANXA13 | 0.897175032 | 2.629817137 | 1.551501109 | 6.59E-05 | 0.000282017 |
| RAB3B | 1.71196242 | 3.467290121 | 1.018157525 | 5.42E-13 | 5.29E-11 |
| HOATZ | 2.182865251 | 1.025652688 | -1.089680794 | 0.011168745 | 0.022532926 |
| AL445205.1 | 1.946553797 | 0.734348185 | -1.406386044 | 0.005840896 | 0.012935398 |
| CGA | 14.16493411 | 33.82208508 | 1.255641712 | 0.004056381 | 0.009517148 |
| LINC02253 | 0.72503758 | 1.517105914 | 1.065194128 | 0.001556664 | 0.00421 |
| COLCA1 | 9.337394981 | 3.919191331 | -1.252464106 | 7.14E-14 | 1.16E-11 |
| RIIAD1 | 1.644460232 | 0.747294691 | -1.137864942 | 5.55E-06 | 3.53E-05 |
| SNORA73B | 399.8854817 | 130.1798617 | -1.619080617 | 0.002188389 | 0.005631267 |
| AC092071.1 | 12.09586615 | 3.61018246 | -1.744370422 | 1.23E-16 | 9.19E-14 |
| BHMT | 2.756145689 | 0.378002755 | -2.866183494 | 0.000550188 | 0.001724443 |
| RPS27AP10 | 1.672318018 | 0.669187298 | -1.321367258 | 0.003948395 | 0.009287891 |
| AC006059.1 | 2.171895238 | 1.04449711 | -1.056146015 | 0.001296171 | 0.003605988 |
| TOGARAM2 | 1.687058945 | 0.833880847 | -1.016597224 | 0.000121555 | 0.00047255 |
| SBK3 | 0.616909781 | 1.45610961 | 1.238987534 | 9.39E-07 | 7.81E-06 |
| VEGFC | 7.382335521 | 18.73533703 | 1.343612717 | 4.41E-09 | 8.62E-08 |
| AL136452.1 | 3.260701673 | 1.449654099 | -1.169473751 | 1.70E-11 | 8.82E-10 |
| SLC16A1 | 11.47044633 | 25.93620625 | 1.177045939 | 1.63E-09 | 3.85E-08 |
| MAFA-AS1 | 3.559607851 | 1.670148387 | -1.091742026 | 0.011374333 | 0.022874686 |
| TRIM29 | 7.932093694 | 19.57171801 | 1.302996778 | 0.002942844 | 0.007236634 |
| SLC15A1 | 1.911198198 | 5.065119489 | 1.406118905 | 1.38E-05 | 7.55E-05 |
| DNAAF1 | 3.874780952 | 1.537711828 | -1.333329592 | 8.76E-05 | 0.000357703 |
| MLXP1 | 1.380117375 | 4.122596102 | 1.578762156 | 0.000165241 | 0.000614831 |
| CLEC4F | 1.354747233 | 0.670340255 | -1.015058222 | 1.14E-09 | 2.93E-08 |
| SLC6A4 | 2.00485547 | 0.940334073 | -1.092252937 | 9.28E-08 | 1.13E-06 |
| IRX6 | 7.760104247 | 3.502296169 | -1.147774944 | 8.35E-10 | 2.22E-08 |
| RAET1G | 0.890004762 | 1.968283871 | 1.145053345 | 0.015664424 | 0.029980264 |
| DLEC1 | 3.470209138 | 1.45759207 | -1.251435597 | 1.98E-09 | 4.52E-08 |
| HHATL | 2.454979151 | 0.357775202 | -2.778585473 | 3.65E-07 | 3.56E-06 |
| PSCA | 31.4285462 | 102.1889136 | 1.701091245 | 0.002588823 | 0.006476393 |
| LINC00973 | 3.373672201 | 8.155742675 | 1.273496454 | 0.001582532 | 0.00427055 |
| TNS4 | 13.83492664 | 39.11757366 | 1.499501893 | 2.67E-08 | 3.90E-07 |
| DKK1 | 11.17917079 | 38.47759765 | 1.783205547 | 6.56E-08 | 8.39E-07 |
| C20orf85 | 47.18237915 | 21.2581994 | -1.150228764 | 0.00351554 | 0.008416387 |
| EYA4 | 0.827536165 | 1.794944086 | 1.117044637 | 0.013442601 | 0.026304869 |
| KYNU | 4.672009781 | 12.06227547 | 1.368386888 | 0.022885673 | 0.041401956 |
| BNC1 | 0.44059601 | 1.87603414 | 2.090157746 | 0.000204352 | 0.000736326 |
| F2RL2 | 1.826057014 | 4.151707997 | 1.184973167 | 0.003353597 | 0.008102874 |
| AC022973.4 | 1.486906821 | 0.733390659 | -1.019660447 | 0.022859375 | 0.041363092 |
| AC108471.1 | 1.514759459 | 0.686107527 | -1.142582116 | 0.026108023 | 0.046300168 |
| LRRC71 | 2.534931145 | 1.006150403 | -1.33310058 | 0.002676743 | 0.006673018 |
| AL513548.4 | 3.22335148 | 1.093411694 | -1.559724801 | 0.000826623 | 0.002439959 |
| AC022497.1 | 2.618772201 | 1.27540578 | -1.037934245 | 3.51E-14 | 6.55E-12 |
| C9orf135 | 10.11562458 | 5.040862836 | -1.004842797 | 7.30E-05 | 0.00030766 |
| LINC02159 | 4.267687387 | 1.670080981 | -1.353536441 | 3.73E-06 | 2.50E-05 |
| SFTPC | 1724.345821 | 368.4896548 | -2.226353117 | 1.07E-11 | 5.84E-10 |
| MARCHF4 | 0.431900386 | 1.975370094 | 2.193352462 | 0.00026333 | 0.000918339 |
| ATP13A4-AS1 | 7.738349678 | 3.093436761 | -1.322815381 | 3.93E-11 | 1.77E-09 |
| CXCL5 | 12.20925457 | 33.00032614 | 1.434505163 | 0.000551217 | 0.001726726 |
| CYP2A6 | 15.01337503 | 1.157176277 | -3.697567776 | 1.69E-08 | 2.66E-07 |
| AL663070.1 | 6.882003475 | 3.245667473 | -1.084313421 | 1.28E-09 | 3.19E-08 |
| CRYBG2 | 2.585690476 | 5.783684005 | 1.161439146 | 7.58E-10 | 2.05E-08 |
| NWD1 | 3.044208752 | 1.494669691 | -1.026240596 | 0.001674886 | 0.004476177 |
| APCDD1L | 0.882539125 | 3.057924933 | 1.792820849 | 8.55E-15 | 2.07E-12 |
| LINC02313 | 0.936050965 | 2.450579032 | 1.388463687 | 0.001012457 | 0.002913888 |
| DPYD-AS1 | 3.65596332 | 1.178745833 | -1.632998925 | 0.000169364 | 0.000628031 |
| CALB2 | 3.304354955 | 14.10649751 | 2.093919252 | 0.023753877 | 0.042734128 |
| SERPINB4 | 2.188711583 | 6.212367944 | 1.505061423 | 3.63E-06 | 2.44E-05 |
| CA4 | 4.578268468 | 1.837393078 | -1.317141766 | 1.34E-07 | 1.55E-06 |
| PPP2R2C | 2.721034492 | 6.614652352 | 1.28151009 | 2.18E-05 | 0.000110882 |
| FAM216B | 8.791664479 | 4.191427755 | -1.068694566 | 0.000378476 | 0.001251534 |
| C9orf24 | 13.18960553 | 5.918716532 | -1.15604515 | 0.000580425 | 0.0018018 |
| INSL4 | 5.442689318 | 12.34206028 | 1.181191655 | 0.000232541 | 0.000823335 |
| CD36 | 11.53187838 | 5.717773858 | -1.01210206 | 1.07E-07 | 1.27E-06 |
| AGTR2 | 13.02270824 | 4.487869288 | -1.536926944 | 0.000440326 | 0.001423478 |
| CAPN3 | 2.250677735 | 0.969103091 | -1.215637448 | 4.51E-17 | 6.46E-14 |
| RN7SL648P | 3.405484427 | 1.273376075 | -1.419201471 | 0.023528974 | 0.042379338 |
| SPINK5 | 29.68175521 | 13.69906841 | -1.115498619 | 0.000912241 | 0.00265347 |
| KRT6A | 52.12945457 | 179.7926149 | 1.786163092 | 2.42E-06 | 1.74E-05 |
| NTRK2 | 2.098525225 | 0.664251747 | -1.659573782 | 6.97E-05 | 0.000295519 |
| SNORA63C | 3.197539125 | 0.767525134 | -2.058676111 | 0.002511383 | 0.006320401 |
| CCDC60 | 1.681619305 | 0.765965255 | -1.134500279 | 1.79E-05 | 9.36E-05 |
| RASGRF1 | 8.078519434 | 2.962525672 | -1.447263256 | 5.40E-10 | 1.56E-08 |
| TEKT1 | 5.884690476 | 2.735287702 | -1.105273948 | 0.004329738 | 0.010045997 |
| GJB5 | 4.26260592 | 9.454041935 | 1.14919558 | 0.000526235 | 0.001659055 |
| PCDHB8 | 1.810636808 | 3.696349194 | 1.029603866 | 1.15E-06 | 9.24E-06 |
| RHOV | 37.5623619 | 94.82286983 | 1.335947275 | 9.98E-06 | 5.76E-05 |
| SNAI2 | 8.02348314 | 16.59578817 | 1.04851657 | 1.73E-07 | 1.90E-06 |
| SPAG8 | 3.329406178 | 1.58444086 | -1.071291074 | 2.90E-10 | 9.29E-09 |
| IGF2BP3 | 3.048223552 | 7.761206116 | 1.348312157 | 3.62E-11 | 1.67E-09 |
| GLB1L3 | 12.39722252 | 5.348826008 | -1.212722755 | 4.74E-07 | 4.43E-06 |
| LGI3 | 6.025802317 | 1.358543078 | -2.149093032 | 3.87E-08 | 5.36E-07 |
| AC079949.2 | 0.983698713 | 2.619882325 | 1.413213593 | 0.005742248 | 0.012769718 |
| NCCRP1 | 7.386886486 | 21.44152151 | 1.537368971 | 0.004722557 | 0.010825835 |
| DNAI1 | 3.661663835 | 1.637351949 | -1.161134886 | 0.016751638 | 0.031682967 |
| CGB5 | 0.475035135 | 10.29868138 | 4.438281596 | 0.003498233 | 0.008385119 |
| ZBBX | 2.231976062 | 1.075542876 | -1.053256515 | 0.011777398 | 0.023539722 |
| LINC02086 | 0.617603475 | 1.451899328 | 1.233188648 | 4.28E-05 | 0.000195849 |
| GDA | 0.987166924 | 2.289117608 | 1.213425624 | 0.018641402 | 0.034733258 |
| Z93930.3 | 1.985072716 | 0.902929167 | -1.136507136 | 1.93E-10 | 6.61E-09 |
| ITGA6 | 28.47189781 | 58.20971573 | 1.031721312 | 2.75E-06 | 1.93E-05 |
| AZU1 | 2.452133848 | 1.018360685 | -1.267789101 | 5.01E-08 | 6.70E-07 |
| AL138963.1 | 1.397122651 | 0.677027016 | -1.045173369 | 0.026055858 | 0.046239997 |
| NKX2-3 | 0.313119048 | 2.008236761 | 2.681146188 | 0.002110171 | 0.005461076 |
| AC026785.3 | 1.963596782 | 4.969170901 | 1.339506452 | 0.000173041 | 0.000639283 |
| RPSAP52 | 0.510735135 | 1.535289516 | 1.587863521 | 2.39E-05 | 0.000119655 |
| AP000695.2 | 0.980942342 | 1.981227285 | 1.014154149 | 1.08E-12 | 9.48E-11 |
| GAS2L2 | 3.839412227 | 1.827507191 | -1.071008384 | 5.84E-08 | 7.61E-07 |
| ABBA01000935.2 | 2.833978636 | 1.353338441 | -1.066306211 | 3.45E-06 | 2.34E-05 |
| NTSR1 | 0.33731583 | 4.083602285 | 3.597670434 | 0.000191017 | 0.000694572 |
| AP000695.1 | 1.454958945 | 2.991277151 | 1.039783142 | 1.39E-15 | 5.98E-13 |
| PVALB | 2.470085714 | 1.001388038 | -1.302559979 | 5.14E-06 | 3.30E-05 |
| RPL35AP2 | 2.165729601 | 0.548896841 | -1.980246186 | 0.00235857 | 0.005998204 |
| B4GALNT2 | 2.173125869 | 4.821347917 | 1.149664802 | 0.013941681 | 0.027154665 |
| CYP4B1 | 136.4733172 | 64.91962198 | -1.071892404 | 4.47E-13 | 4.57E-11 |
| PSMD10P2 | 0.742077477 | 1.852299798 | 1.319675894 | 6.39E-12 | 3.88E-10 |
| ELF5 | 11.15794093 | 5.155052755 | -1.114011722 | 1.09E-08 | 1.84E-07 |
| CIDEC | 0.527629472 | 2.236542406 | 2.083673055 | 6.02E-05 | 0.000260417 |
| STC1 | 18.31245341 | 40.98153804 | 1.162149041 | 8.99E-09 | 1.56E-07 |
| MIR548V | 3.454712613 | 1.10666922 | -1.642341632 | 0.016725678 | 0.031637393 |
| FOXI1 | 1.488097941 | 0.65968918 | -1.173611134 | 0.012235001 | 0.024318533 |
| APOBEC1 | 1.939765766 | 4.345790591 | 1.163736206 | 3.37E-06 | 2.29E-05 |
| C6 | 3.553358044 | 1.613616062 | -1.138885715 | 6.43E-06 | 3.99E-05 |
| RGS4 | 2.683489189 | 5.701394489 | 1.087204751 | 1.48E-05 | 7.99E-05 |
| CTSV | 4.832094337 | 10.07479899 | 1.060030529 | 5.03E-12 | 3.23E-10 |
| CRYM | 36.05073745 | 17.37240363 | -1.053231394 | 5.00E-07 | 4.62E-06 |
| CDHR4 | 5.151648777 | 2.235999866 | -1.204114137 | 0.012395379 | 0.024567301 |
| TRIM15 | 1.230747233 | 3.388231653 | 1.460998019 | 1.92E-08 | 2.97E-07 |
| MIR3671 | 4.903399871 | 2.27755457 | -1.106296797 | 2.78E-09 | 5.93E-08 |
| BCL2L10 | 1.299446976 | 2.929162298 | 1.172590367 | 2.84E-05 | 0.000138883 |
| GAS6-AS1 | 3.510856885 | 1.613055309 | -1.122027281 | 5.87E-12 | 3.63E-10 |
| PICSAR | 1.081037194 | 3.008542406 | 1.47664853 | 0.003130207 | 0.007631823 |
| ADGRF4 | 3.657595882 | 9.410346371 | 1.363352142 | 9.84E-14 | 1.47E-11 |
| HOXC12 | 1.281865637 | 3.517508065 | 1.456308682 | 0.014228328 | 0.02763777 |
| AC005291.2 | 0.535611712 | 1.760285618 | 1.716550123 | 0.000126258 | 0.000488842 |
| GNMT | 3.268332947 | 1.61561297 | -1.016473326 | 1.35E-18 | 4.64E-15 |
| FBN2 | 0.626129215 | 3.920197312 | 2.646393947 | 0.001613506 | 0.004338472 |
| MBL1P | 3.8138713 | 1.83810672 | -1.053035628 | 1.77E-12 | 1.44E-10 |
| LINC01605 | 0.73299408 | 1.509688575 | 1.042377524 | 0.000734089 | 0.002203935 |
| MUC2 | 0.204385714 | 2.128877218 | 3.380726479 | 0.00033368 | 0.00112442 |
| AL513008.1 | 2.207973359 | 0.904978562 | -1.286767243 | 3.84E-05 | 0.00017842 |
| ANKRD44-AS1 | 5.921630373 | 2.754311761 | -1.104302573 | 1.97E-05 | 0.000101578 |
| AC011944.1 | 1.457085071 | 0.62470961 | -1.221827482 | 8.91E-07 | 7.47E-06 |
| AC092868.1 | 4.002550064 | 10.41560638 | 1.37975548 | 0.024464579 | 0.043806233 |
| RNU4-1 | 120.5651456 | 45.72875081 | -1.398639482 | 0.000747287 | 0.002236958 |
| FGA | 89.87851918 | 584.9459188 | 2.702254986 | 0.018473217 | 0.034472235 |
| PRSS3 | 2.250416731 | 9.943793011 | 2.143604082 | 0.000302058 | 0.001032033 |
| MMP2-AS1 | 3.223010296 | 1.486074731 | -1.11690213 | 1.23E-09 | 3.10E-08 |
| AFP | 0.301620463 | 9.766225202 | 5.01699483 | 0.00127683 | 0.003560243 |
| CLDN6 | 29.01618958 | 63.21514859 | 1.123412244 | 0.026572891 | 0.046999453 |
| RNU6-247P | 5.061450708 | 1.649193145 | -1.617790578 | 0.002208235 | 0.005672989 |
| PGM5P4 | 1.672286615 | 0.835444691 | -1.001205908 | 4.59E-08 | 6.22E-07 |
| AC006270.1 | 2.748314414 | 1.131844556 | -1.279871225 | 0.009227454 | 0.019193417 |
| REG4 | 29.1671574 | 87.35690249 | 1.582576912 | 0.025256244 | 0.045024914 |
| AC010998.3 | 2.455923552 | 1.026297984 | -1.258815977 | 2.07E-07 | 2.22E-06 |
| CHIAP2 | 2.88268018 | 0.784082325 | -1.878333741 | 9.11E-12 | 5.20E-10 |
| RNU4-2 | 438.5169081 | 118.4386534 | -1.888492478 | 0.003049269 | 0.007465213 |
| KRT17 | 67.40102188 | 225.5960618 | 1.742899513 | 0.000139697 | 0.000532354 |
| UCA1 | 1.192899743 | 3.685079637 | 1.627223001 | 0.003377788 | 0.008152152 |
| LINC01711 | 0.689926126 | 1.515198992 | 1.134993477 | 2.07E-11 | 1.05E-09 |
| SERPIND1 | 36.52272291 | 9.915275538 | -1.881069558 | 2.60E-06 | 1.84E-05 |
| GSTA1 | 89.27388816 | 43.60533817 | -1.0337335 | 0.000429016 | 0.001392943 |
| AKAP12 | 11.56636615 | 40.32904005 | 1.801883387 | 3.16E-07 | 3.15E-06 |
| VWA3A | 3.234021364 | 1.260462298 | -1.359376243 | 6.65E-06 | 4.09E-05 |
| GNG4 | 4.629754698 | 10.85511129 | 1.229366856 | 6.28E-08 | 8.11E-07 |
| PDX1 | 1.049335393 | 2.745897513 | 1.387801909 | 8.12E-06 | 4.85E-05 |
| AC007849.1 | 1.867971557 | 0.560639247 | -1.73632784 | 0.003652852 | 0.008693868 |
| ARMC2-AS1 | 1.737930888 | 0.739175538 | -1.233381794 | 5.20E-05 | 0.000230197 |
| RN7SL8P | 2.678017117 | 0.945760349 | -1.501618618 | 7.17E-12 | 4.26E-10 |
| C16orf89 | 529.1843931 | 244.4388485 | -1.114296924 | 1.09E-13 | 1.60E-11 |
| SCGB1A1 | 388.1231003 | 130.8475571 | -1.568627312 | 0.000317068 | 0.001075404 |
| C6orf118 | 2.940915959 | 1.312587567 | -1.163851884 | 0.002561375 | 0.006424567 |
| BTNL9 | 5.625698842 | 2.157385148 | -1.382748566 | 4.12E-14 | 7.38E-12 |
| AC244258.1 | 5.729213256 | 1.487085753 | -1.945849197 | 0.019304795 | 0.035771447 |
| SERPINB3 | 6.089119949 | 17.20316694 | 1.498368538 | 0.000302621 | 0.001033544 |
| BMX | 3.274444788 | 1.491158535 | -1.134816657 | 0.005307146 | 0.011945961 |
| MACROD2 | 9.483949807 | 4.079999126 | -1.216919184 | 1.73E-06 | 1.31E-05 |
| ADAMTS7P3 | 1.784730116 | 0.41512164 | -2.104099884 | 3.49E-09 | 7.10E-08 |
| CAMP | 3.223970528 | 1.608870228 | -1.002790592 | 4.32E-05 | 0.0001973 |
| IRX1 | 4.109471042 | 2.050203696 | -1.003185452 | 2.79E-06 | 1.96E-05 |
| MMP3 | 2.650716474 | 5.491394624 | 1.050790226 | 0.001108576 | 0.003151117 |
| AL139041.1 | 3.308707207 | 1.484903495 | -1.155898459 | 3.19E-15 | 1.01E-12 |
| BNIP3P40 | 1.535449292 | 0.763183132 | -1.008559679 | 0.001596598 | 0.00429838 |
| RNA5SP282 | 2.111066538 | 0.807372513 | -1.386665674 | 0.026687288 | 0.047152208 |
| TTLL10 | 1.833355856 | 0.739882594 | -1.309118577 | 0.000207624 | 0.000746349 |
| RN7SKP51 | 4.479745431 | 2.203825336 | -1.023406863 | 2.77E-13 | 3.15E-11 |
| SPOCK1 | 5.662916474 | 12.86402675 | 1.183725156 | 3.27E-10 | 1.02E-08 |
| GGTLC1 | 63.21792085 | 29.09332601 | -1.11964535 | 3.07E-10 | 9.68E-09 |
| GSTA3 | 1.57126834 | 0.758436559 | -1.05082917 | 6.05E-08 | 7.86E-07 |
| E2F7 | 1.696577091 | 3.636236559 | 1.099819072 | 9.43E-11 | 3.72E-09 |
| GPR1 | 0.60483964 | 1.442909946 | 1.254356664 | 3.78E-08 | 5.27E-07 |
| AC019117.2 | 1.679853153 | 0.763969825 | -1.136747561 | 3.35E-05 | 0.000159546 |
| HHIP | 5.825553539 | 2.630442137 | -1.147089825 | 5.42E-06 | 3.45E-05 |
| CNMD | 58.48088327 | 7.539979435 | -2.955332607 | 0.000991837 | 0.002861735 |
| Z99496.1 | 3.961495238 | 0.504370228 | -2.973490043 | 0.001030348 | 0.002959614 |
| NPTX2 | 4.835312098 | 15.02916794 | 1.636084223 | 0.000728983 | 0.00219052 |
| DTHD1 | 2.650624453 | 1.319152487 | -1.006720939 | 0.002928334 | 0.007211274 |
| RNU4ATAC | 7.060192021 | 1.782920363 | -1.985465158 | 0.000278626 | 0.000963468 |
| CFAP100 | 2.025604633 | 0.890654234 | -1.185415241 | 2.59E-05 | 0.000128201 |
| AC141273.2 | 2.753727156 | 1.36131129 | -1.016388617 | 4.72E-07 | 4.41E-06 |
| LINC00578 | 8.154035393 | 3.851160417 | -1.082221 | 5.03E-11 | 2.19E-09 |
| ARHGEF4 | 2.159516731 | 4.371434073 | 1.017398147 | 6.72E-10 | 1.86E-08 |
| ADH1C | 65.80877091 | 23.95989375 | -1.457658367 | 0.022488797 | 0.040821538 |
| ZDHHC11B | 8.458405405 | 4.186269422 | -1.014720545 | 1.53E-11 | 7.99E-10 |
| AC114760.2 | 1.803030631 | 0.790970968 | -1.188727259 | 0.004913632 | 0.011187858 |
| CIBAR2 | 8.805601673 | 3.98754375 | -1.142921237 | 0.00145672 | 0.003977271 |
| LRRC36 | 4.94393758 | 1.501819556 | -1.718949045 | 8.36E-16 | 3.99E-13 |
| SCGB3A1 | 2955.684393 | 425.2143869 | -2.797229912 | 2.84E-11 | 1.36E-09 |
| TBXT | 2.027671815 | 0.68126297 | -1.573540469 | 0.018687667 | 0.03481191 |
| SYNDIG1L | 1.55621686 | 0.772184073 | -1.011026413 | 3.47E-11 | 1.62E-09 |
| ADH7 | 5.306119305 | 1.634975806 | -1.698387826 | 0.000145352 | 0.000550242 |
| CFAP52 | 4.197411969 | 1.863560081 | -1.171438736 | 0.000267425 | 0.000930546 |
| RTN4RL1 | 6.008265894 | 2.752093817 | -1.126419009 | 3.01E-09 | 6.33E-08 |
| AC115099.1 | 1.560405405 | 0.642491398 | -1.280171857 | 4.77E-09 | 9.19E-08 |
| ADAMTS8 | 5.672709524 | 2.516062836 | -1.172870039 | 4.23E-10 | 1.27E-08 |
| TCTEX1D1 | 2.026262291 | 0.916124328 | -1.145205631 | 5.92E-05 | 0.000257339 |
| MIR663AHG | 2.098104891 | 0.943793481 | -1.152543693 | 2.72E-12 | 2.02E-10 |
| GJB4 | 0.837915444 | 2.010240659 | 1.262491655 | 4.42E-05 | 0.000201078 |
| MAGEC1 | 1.404307079 | 3.076545632 | 1.131452948 | 0.007996913 | 0.016949903 |
| AC108451.2 | 0.733325483 | 2.184894758 | 1.575038212 | 0.014910767 | 0.02877567 |
| SPINK13 | 6.974859331 | 1.66511203 | -2.066544875 | 7.85E-05 | 0.000326818 |
| SCGB3A2 | 1562.933243 | 755.365963 | -1.049008476 | 2.16E-08 | 3.27E-07 |
| FENDRR | 2.799996525 | 1.367265121 | -1.034132019 | 1.56E-06 | 1.20E-05 |
| SNORA74B | 12.98932523 | 3.282277083 | -1.984557548 | 0.023035356 | 0.041663969 |
| TEPP | 3.11811287 | 1.551988306 | -1.006555464 | 1.59E-10 | 5.69E-09 |
| TUBA4B | 5.350789833 | 2.623490591 | -1.028264248 | 0.019083825 | 0.035417814 |
| RPL26P30 | 7.629514157 | 3.031773118 | -1.331429396 | 2.37E-07 | 2.48E-06 |
| LINC02323 | 0.797642857 | 2.010294153 | 1.333591784 | 9.93E-10 | 2.59E-08 |
| KIF26B-AS1 | 1.940619949 | 0.950877083 | -1.029186842 | 0.002074806 | 0.005380088 |
| AGER | 168.1051552 | 71.1241541 | -1.240952474 | 5.25E-10 | 1.52E-08 |
| LCAL1 | 31.15675187 | 14.28154315 | -1.125392965 | 0.000735439 | 0.002206831 |
| SNORA80E | 3.87614556 | 1.751692204 | -1.145873452 | 0.000595662 | 0.001840776 |
| AL121938.1 | 2.735767053 | 0.773997984 | -1.821543678 | 0.021265287 | 0.038867577 |
